# Functional Genomic Complexity Defines Intratumor Heterogeneity and Tumor Aggressiveness in Liver Cancer

**DOI:** 10.1038/s41598-019-52578-8

**Published:** 2019-11-15

**Authors:** So Mee Kwon, Anuradha Budhu, Hyun Goo Woo, Jittiporn Chaisaingmongkol, Hien Dang, Marshonna Forgues, Curtis C. Harris, Gao Zhang, Noam Auslander, Eytan Ruppin, Chulabhorn Mahidol, Mathuros Ruchirawat, Xin Wei Wang

**Affiliations:** 1 Laboratory of Human Carcinogenesis and Liver Cancer Program, Center for Cancer Research, National Cancer Institute, Bethesda, Maryland 20892 USA; 2 Department of Physiology, Ajou University School of Medicine, Suwon, 16499 Republic of Korea; 3 Department of Biomedical Science, Graduate School, Ajou University, Suwon, 16499 Republic of Korea; 4 Laboratory of Chemical Carcinogenesis, Chulabhorn Research Institute, Bangkok, 10210 Thailand; 5 Center of Excellence on Environmental Health and Toxicology, Office of the Higher Education Commission, Ministry of Education, Bangkok, 10400 Thailand; 6 Molecular and Cellular Oncogenesis Program and Melanoma Research Center, The Wistar Institute, Philadelphia, PA 19104 USA; 7 Cancer Data Science Lab, National Cancer Institute, National Institute of health, MD, 20892 USA

**Keywords:** Cancer genomics, Tumour heterogeneity, Data integration

## Abstract

Chronic inflammation and chromosome aneuploidy are major traits of primary liver cancer (PLC), which represent the second most common cause of cancer-related death worldwide. Increased cancer fitness and aggressiveness of PLC may be achieved by enhancing tumoral genomic complexity that alters tumor biology. Here, we developed a scoring method, namely functional genomic complexity (FGC), to determine the degree of molecular heterogeneity among 580 liver tumors with diverse ethnicities and etiologies by assessing integrated genomic and transcriptomic data. We found that tumors with higher FGC scores are associated with chromosome instability and *TP53* mutations, and a worse prognosis, while tumors with lower FGC scores have elevated infiltrating lymphocytes and a better prognosis. These results indicate that FGC scores may serve as a surrogate to define genomic heterogeneity of PLC linked to chromosomal instability and evasion of immune surveillance. Our findings demonstrate an ability to define genomic heterogeneity and corresponding tumor biology of liver cancer based only on bulk genomic and transcriptomic data. Our data also provide a rationale for applying this approach to survey liver tumor immunity and to stratify patients for immune-based therapy.

## Introduction

PLC is the second leading cause of cancer-related mortality in the world^1,2^. Hepatocellular carcinoma (HCC) and intrahepatic cholangiocarcinoma (iCCA) are two main histological subtypes of PLC, with molecular subtypes that differ in tumor biology and prognosis^3–8^. Like other solid malignant tumors, HCC and iCCA are genomically, molecularly and biologically heterogeneous among individual tumors (inter-tumor) or within tumor lesions (intra-tumor)^3,4,6,9–13^. Genomic instability and chromosome aneuploidy, two main cancer hallmarks found in human solid tumors^14^, may be primary sources of cancer genomic diversity, which enable cancer cells to acquire mutations required for tumor fitness during carcinogenesis. Consequently, diverse tumor cell subpopulations are generated, resulting in both inter-tumor and intra-tumor heterogeneities (ITH)^15^. We hypothesized that the ability of a premalignant cell to acquire genomic instability and chromosomal aneuploidy, giving rise to a more advanced tumor lesion, may determine the extent of ITH. Thus, a solid tumor mass with increased genomic complexity enriched with known hallmarks of cancer may determine its aggressiveness. Furthermore, recent genomic analysis at the single-cell resolution indicates that aneuploidy occurs early in tumor evolution and may lead to extensive clonal diversity^16^. However, a majority of genes in the loci with aneuploidy may be passengers^17^. In contrast, chromosomal instability signature, inferred from the transcriptome, has been shown to associate with tumor metastasis and poor patient prognosis in diverse cancer types, indicating that its associated genes are more likely to be functional^18^. These acquired traits collectively derived from the altered genome and functional networks, which we refer to as cancer functional genomic complexity (FGC), analogous to proposed functional variomics^17^, may reflect the degree of ITH linked to tumor aggressiveness. In this study, we derived FGC in individual tumors based on the newly defined method and determined whether it is correlated with the degree of tumor aggressiveness in HCC and iCCA.

## Results

### The patient correlation coefficient (PCC) defines functional genomic complexity (FGC)

**Figure 1:**
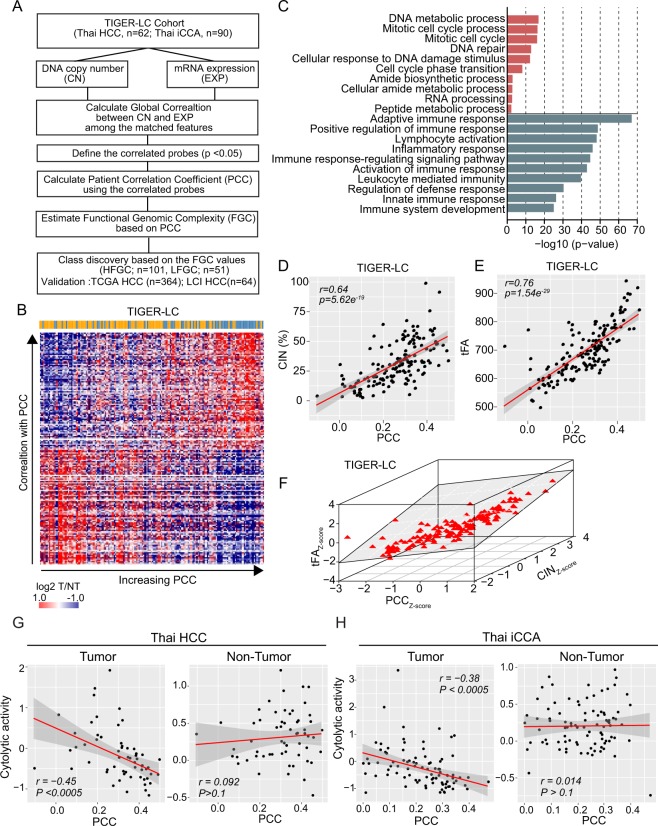
The patient correlation coefficient (PCC) defines functional genomic complexity (FGC). (**A**) Schematic overview of the study design. (**B**) Heatmap shows the expression level of positively or negatively PCC associated genes (>95% or <5% of correlation estimate and p-value <0.01) in TIGER-LC. Samples are represented according to the PCC increasing order in columns and genes were represented according to the decreasing of correlation order in coefficient in the row. The color bar indicates HCC and iCCA patients as in blue and orange color, respectively. (**C**) GO Enrichment Analysis of selected genes. Top10 ranked GO based on the precision rank were shown. Orange and green color indicates positively and negatively correlated gene sets, respectively. (**D,E**) PCC shows strong correlation with CIN (**D**) and tFA (**E**). Coefficient estimates and p-value based on Pearson’s correlation were depicted. (**F**) The collective association among PCC (x-axis), CIN (y-axis), and tFA (z-axis) are shown. (**G,H**) The association between PCC and Immune Cytolytic activity (ICYT), defined as log-average of GZMA and PRF1 expression, derived from tumor (left panels of each) or non-tumor (right panels of each) tissue of Thai HCC and Thai iCCA are shown, respectively.

Since it is generally accepted that somatic copy number alteration (SCNA)-dependent transcriptomic deregulation plays functional driver roles in cancer progression, we hypothesized that the estimate of the SCNA-dependency of transcripts for each patient may reflect genomic complexity with functional influence. We postulated that the SCNA-dependency of transcripts in the individual sample could be estimated based on the correlation between SCNAs and their corresponding transcriptome using the globally correlated features. To demonstrate this, first, we determined the SCNA-dependency in the tumor (T) and non-tumor (NT) tissues by performing a global correlation analysis between transcriptome and DNA copy number in the TIGER-LC cohort^4^ (Methods and Fig. 1A). This analysis revealed that the overall distribution of global correlation estimates was shifted towards the positive correlation of T (shaded curves) samples of Thai HCC and iCCA, compared to their corresponding NT specimens (dotted areas) (Fig. S1A,B, left panels). These results solidified our assumption of SCNA-dependent transcriptomic deregulation was tumor-specific and may play functional driver roles in cancer progression.

After selecting the significantly correlated features using the conservative approach (permutated p-value < 0.05), we compared the correlation estimates of selected features between T and NT. This analysis revealed that a positive correlation was more apparently enriched in T samples than NT samples (Methods and Fig. S1A,B, right panels), indicating that SCNA-dependent molecular features are tumor-specific. Using these features, we computed PCC per sample (Methods and Table S1a–c). Next, to examine the biological relevance of the PCC with genomic complexity (Fig. 1B), we selected positively or negatively PCC associated genes based on the correlation test between PCC and transcriptome (top 5% or bottom 5% of correlation estimates and p-value < 0.05, respectively). Gene Ontology (GO) analysis revealed that biological processes regarding DNA repair and cell cycle were enriched among the positively associated genes, while immune response pathways were enriched among the negatively associated genes (Fig. 1C). Wel also observed consistent results in the Thai HCC and iCCA separately as well as TCGA HCC (Fig. S2A–F). Since, among positively associated genes, most of the over-represented processes such as cell cycle and DNA damage response (DDR) are closely related with aneuploidy and chromosomal instability, we asked whether PCC is associated with chromosomal instability. For the estimates of chromosomal instability, we calculated the chromosomal instability score (CIN) and genomic instability score (GIN) using the copy number data, indicating the frequency and length of SCNA segments for each sample, respectively (Supplementary Materials and Methods and Table S1a,b). Consistent with the GO analysis, we observed that PCC was positively correlated with both CIN and GIN (Figs 1D and S3A–E), suggesting a strong association of PCC with chromosomal instability. To survey the preferential allelic gain or loss linked to PCC, we compared the association of PCC with the amplified (CIN_ampl_) and deleted (CIN_del_) scores for individual samples (Supplementary Materials and Methods and Fig. S4A,B). However, we found no evidence indicating the preponderance of PCC toward copy number gain or loss. Interestingly, a strong association between CIN_ampl_ and CIN_del_ was observed in both Thai HCC and iCCA (Fig. S4C,D). Also, the broad chromosomal arm-level SCNA analyses based on the GISTIC 2.0 algorithm showed consistent trends in HCC and iCCA (Fig. S4E,F). These results suggest that gains or losses of specific chromosomal regions may be a consequence of tumor cells acquiring genomic instability and then being selected during tumor evolution. However, it is likely that a majority of genes in the loci with aneuploidy may be passengers. Therefore, we determined whether the PCC also reflected the aneuploidy of the genes with a functional role. For this, we inferred the total functional aneuploidy (tFA) (Table S1a,b) from transcriptome data using the adapted method in Carter’s paper^18^. Briefly, we calculated tFA in each sample based on the coordinated aberrations in the expression of genes, which were localized to each chromosomal region (Supplementary Materials and Methods). Since tFA was the estimate of the aneuploidy reflected transcriptome level and more likely to be functional^18^, we examined the functional relevance between PCC and tFA. When we implemented GO analysis with significantly tFA associated genes (top 5% or bottom 5% of correlation estimates and p-value < 0.05, respectively), we found the similar GO terms found in PCC, including mitotic cell cycle, DNA repair, and RNA processing, and immune response, were enriched in tFA (Figs 1C and S5A–F). Likewise, we found a strong association of PCC with tFA in the TIGER-LC cohort (Fig. 1E). We reaffirmed a strong association between PCC and tFA when we examined Thai HCC and iCCA separately as well as TCGA HCC (Fig. S5G–I). Moreover, we found a high concordance among CIN, tFA, and PCC scores (Figs 1F and S6). These results suggest that PCC strongly represents the tumor aneuploidy with functional relevance.

Interestingly, GO analysis revealed that genes relevant to immune response pathways were enriched among the PCC negatively associated genes (Figs 1C and S2A–C), implying that PCC may negatively affect cancer immunity. Since anti-tumor activities of effector T cells at the tumor sites are associated with patient prognosis in solid tumor^19,20^, we focused on the immune effector activity of the local immune infiltrates. For this, we calculated immune cytolytic activity based on the transcript levels of two key cytolytic effectors, granzyme A (*GZMA*) and perforin (*PRF1*)^21^. Consistent with the GO analysis, we found a strong inverse correlation between cytolytic activity and PCC in the tumor tissues. However, there was no association in adjacent non-tumor tissues in Thai HCC and iCCA (Fig. 1G,H), indicating that these associations were only linked to local immune infiltrates in tumor and PCC may reflect the immune surveillance in tumor tissue. Furthermore, we examined whether the inverse correlation could result from the sampling of predominantly immune vs predominantly cancer cells. For this, we estimated the tumor purity based on classical image analysis (IHC) and computational methods (ESTIMATE^22^ and ABSOLUTE^23^) (Table S1a,b**)**. Using these methods, we excluded the samples with low tumor purity to inspect whether the effect of PCC on immune cytolytic activity was affected by tumor purity. In this analysis, we observed that PCC was negatively correlated with immune cytolytic activity even after removing the samples with low tumor purity (Fig. S11A,B). Given these results, we assert that the inverse correlation of PCC with immune cells was mainly derived from the cancer cell itself. Taken together, these results indicate that PCC represents functional genomic complexity (FGC) conversantly reflecting both chromosome level and transcriptome level and selection of drivers during tumorigenesis. Therefore, we named PCC as “FGC” to refer to the newly defined genomic complexity estimate in an individual sample.

### Stratification PLC patients based on the FGC shows the clinical significance of FGC

**Figure 2:**
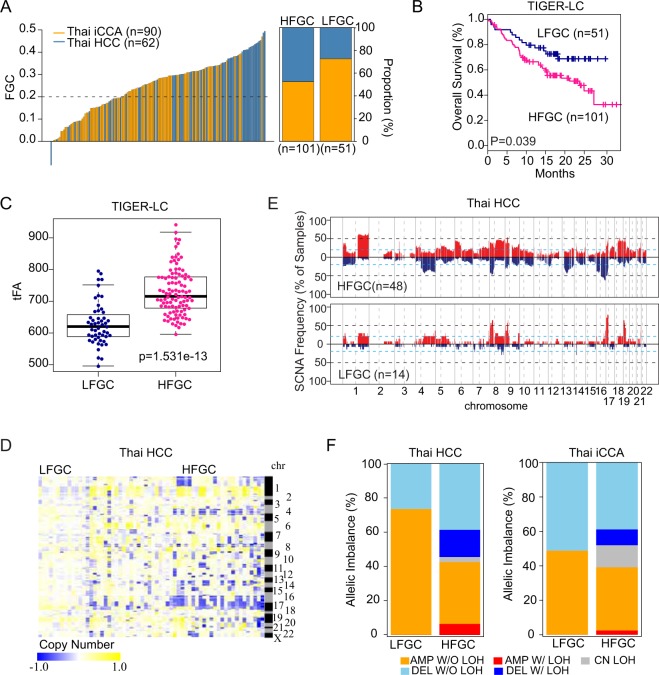
Stratification of PLC patients based on the FGC (**A**) FGC values among the TIGER-LC cohort (n = 152) are plotted in rank order (left). By applying cut-off (dotted line, FGC = 0.2), TIGER-LC cohort was separated into an FGC high (HFGC; n = 101) and FGC low (LFGC; n = 51) group. The relative proportion of HCC and iCCA among HFGC and LFGC are shown (right). HCC and iCCA patients are shown in blue and orange colors, respectively. (**B**) Kaplan-Meier (KM) survival analysis based on the HFGC and LFGC subtype show significant difference in overall survival time. The statistical P-value by the Cox-Mantel log-rank test was depicted. (**C**) HFGC shows higher tFA values compared to the LFGC. P-value based on Welch’s two-sample t-test was depicted. (**D**) Heatmap shows copy number value of individual sample of Thai HCC corresponding to the correlated segments regions, respectively. Samples are grouped by the HFGC and LFGC in columns and segment regions are represented in rows according to the chromosomal location. (**E**) The frequency of SCNA among HFGC and LFGC subtype of Thai HCC are plotted corresponding to the correlated segmented region, respectively. The sample frequencies with copy number gain and loss (log2 (copy number) >0.2 or log2 (copy number) < −0.2) are shown in red and blue, respectively. Chromosome boundaries and centromere positions are indicated by vertical solid and dashed lines, respectively. Horizontal dashed blue lines indicate frequency of 50%. Horizontal dotted black lines indicate frequency of 20%. (**F**) Comparison of allelic imbalance frequency between HFGC and LFGC. The proportion of allelic imbalance of HFGC and LFGC of Thai HCC and Thai iCCA are plotted.

To evaluate the clinical relevance of FGC, we divided 152 cases into a high (HFGC) or low group (LFGC) based on the FGC value (≥0.2) (left panel of Fig. 2A and Table S1a) and found a significant difference in overall survival between HFGC and LFGC, with HFGC being more aggressive than LFGC (Fig. 2B). It was noted that more HCC cases were found in HFGC whereas more iCCA cases were found in LFGC (Fig. 2A, right panel) and the difference was statistically significant (p-value of 2.42e^−6^, Wilcoxon rank-sum test). We observed similar results when Thai HCC and iCCA cohorts were analyzed separately (Fig. S7A–D and Table S1a) and in the Cancer Genome Atlas (TCGA) HCC cohort and LCI Chinese HCC cohort (Fig. S7E–H and Table S1b,c), indicating that FGC scores are robust indicators of PLC prognosis. Furthermore, HFGC had a significantly higher level of tFA than LFGC (Figs 2C and S8A–C). Evidence for the difference in genomic instability between HFGC and LFGC was also found when we compared the frequency and magnitude of SCNA the among individual samples, showing that HFGC had a higher magnitude and frequency of SCNA than LFGC (Figs 2D,E and S9A–D). In addition, HFGC had much higher GIN scores, showing a significant difference in both gain and loss compared to LFGC (Fig. S9E,F). Moreover, upon examining allelic imbalance, only HFGC samples contained SCNA regions with loss of heterozygosity (LOH) in Thai HCC and iCCA (Fig. 2F). Interestingly, the total number of peak segments was noticeably higher in HCC HFGC than iCCA HFGC, accounting for more deleted segments with LOH (DEL W/LOH) than amplified segments with LOH (AMP W/LOH) (Fig. S9G). Furthermore, the region for copy neutral LOH (CN LOH; segments with LOH without SCNA) was only found in the HFGC subtype in HCC and iCCA (Fig. S9H, upper panel). Interestingly, CN LOH regions in iCCA HFGC were found throughout all genomic regions, while those in HCC HFGC were focally enriched in the specific chromosomal arms (Fig. S9H, lower panel). Considering the likelihood of CN LOH to activate oncogenes and to unmask tumor suppressers^24^, the high incidence of CN LOH in the HFGC subtype implies a strong contribution of genomic instability to carcinogenesis.

### Determination of candidate drivers based on the differentially expressed genes (DEGs) between HFGC and LFGC

**Figure 3:**
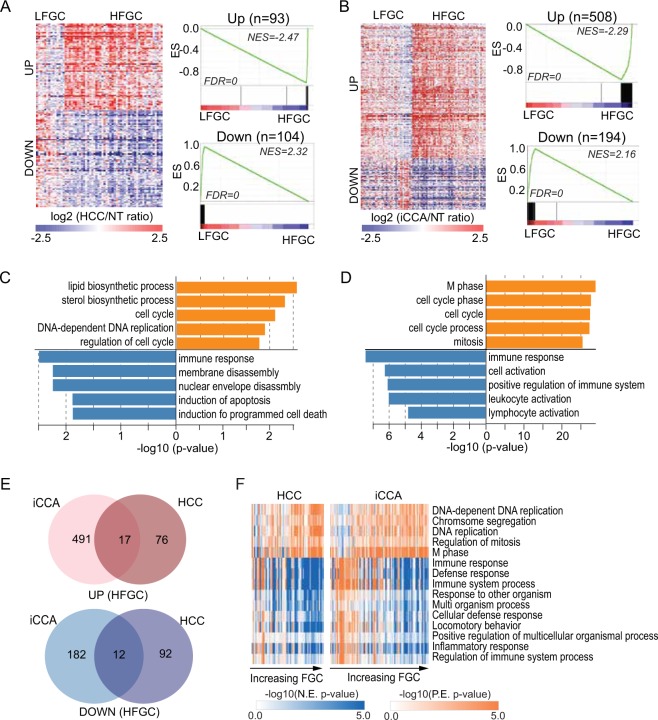
Differentially expressed genes (DEG) between HFGC and LFGC. (**A,B**) (Left panel of each) A heatmap shows the expression of DEG between HFGC and LFGC of Thai HCC (**A**) and Thai iCCA (**B**) Samples are represented in columns, grouped by HFGC and LFGC, and up- or down-regulated genes are represented in rows. (Right panel of each) Enrichment plots based on the 93 upregulated genes and 104 down-regulated genes are shown in the upper and lower panel, respectively. Normalized enrichment scores (NES) and FDR for each gene set were noted. (**C,D**) GO analysis performed based on the DEGs of Thai HCC and Thai iCCA, respectively. The −log10 (p-value) is shown in orange and blue bars for up- and down-regulated genes, respectively. (**E**) Venn diagrams show the overlapping genes between DEG of Thai HCC and of Thai iCCA. Up- and down-regulated genes are analyzed separately. (**F**) Heatmaps show the ssGSEA of Thai HCC (left) and Thai iCCA (right) based on the gene sets derived from the biological process (BP) gene sets in Molecular Signatures Database (MSigDB database v5.2). The overlapping gene sets significantly enriched in both Thai HCC and Thai iCCA are shown. The P-value from the Kolmogorov-Smirnov (ks) test was transformed in -log scale and used in the plot. Samples were in columns according to the increasing order of FGC value and the log-transformed p-value for each gene set is represented in rows.

To determine additional candidate drivers of HCC and iCCA, we selected DEGs between HFGC and LFGC (permutation t-test p < 0.005 and fold change >0.5) among the globally correlated genes (Fig. 3A,B). We found that the upregulated genes in HFGC of both Thai HCC and iCCA were significantly enriched with cell cycle and DNA replication process-related genes, while immune response-related genes were down-regulated (Fig. 3C–E and Table S2), with similar results found in TCGA HCC (Fig. S10A,B and Table S3). In addition, the gene-set enrichment analysis (GSEA), based on biological process, KEGG pathway, and oncogenic pathway, revealed that a considerable number of pathways were overlapped between Thai iCCA and HCC among the top-ranked 40 pathways (15, 29, and 27 pathways, respectively) (Figs 3F, S12, and Table S4,5). Also, consistent results were found in TCGA HCC (Fig. S10C,D and Table S6). These results suggest that, regardless of PLC tumor type, shared common molecular features may contribute to the tumorigenesis of HFGC and LFGC, with distinct tumor biology according to the subtypes (Table S7).

### TP53 mutations are associated with increased FGC in PLC

**Figure 4:**
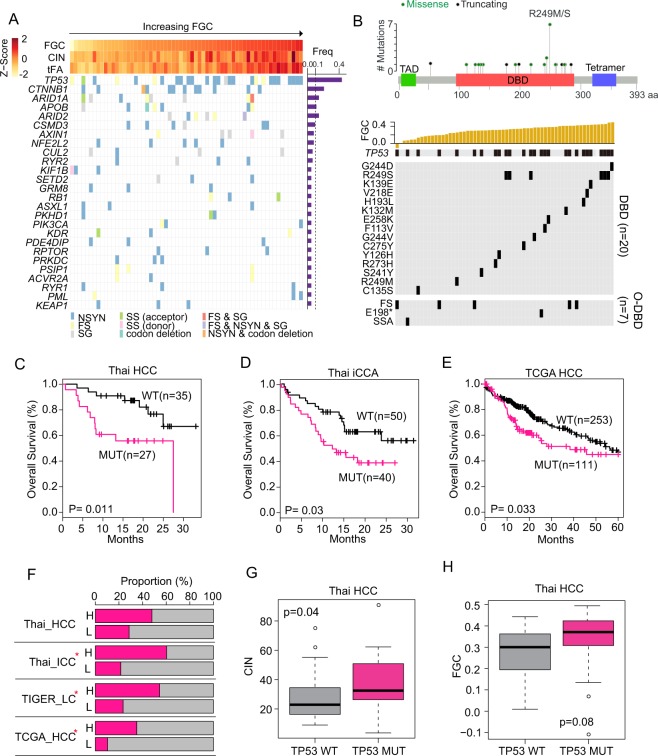
*TP53* functions as a cancer functional genomic complexity (FGC) driver. (**A**) (Top panel) Z-scores for FGC, CIN, and tFA in each HCC sample are plotted in each barplot in the FGC ranked order. (Bottom panel) The mutation frequency for 26 genes, mutated in more than 3 samples of Thai HCC, was shown (right panel). The occurrence of mutation of regarding gene in each sample and mutation types were indicated in different colors. Samples were represented in columns in the same order of top panel. (**B**) (Top panel) *TP53* mutations sites among Thai HCC were shown. Transactivation motif (TAD; 6–29), DNA binding motif (DBD; 95–288), and tetramerization motif (Tetramer; 318–358) were depicted in a different colored box; green, orange, and navy, respectively. Green or black dots indicate missense or truncating mutation, respectively. (Bottom panel) The top plot indicates the FGC score of each sample in the rank order. *TP53* mutation incidence in each sample was plotted in black according to the mutation sites. Mutation sites of *TP53* depending on DNA binding domain (DBD) and out of DBD (O-DBD) were plotted separately. (**C**–**E**) KM survival analysis based on *TP53* mutation status in the Thai HCC (**C**), Thai iCCA (**D**), and TCGA HCC (**E**) patients. (**F**) The proportion of occurrence of *TP53* mutation in HFGC and LFGC of TIGER-LC, Thai HCC, iCCA, TCGA HCC were shown. H or L indicates HFGC and LFGC, respectively. Significant enrichment of *TP53* mutation was marked with red star based on the Fisher’s exact test (p-value < 0.05). (**G**,**H**) The CIN (**G**) and FGC (**H**) level between *TP53* WT and *TP53* mutation among Thai HCC. P-values based on the Welch two-sample t-test were depicted. NSYN, non-synonymous mutation; FS, frameshift mutation; SS, splice site mutation; NS, nonsense mutation; SSA, splice site acceptor.

Next, we examined whether specific gene mutations are associated with FGC. A total of 100 mutated genes were commonly found in HCC and iCCA (Table S8–10). Noticeably, patients with higher FGC scores exhibited more frequent *TP53* mutations, particularly in nonsynonymous mutations (Figs 4A and S13A). Most *TP53* mutations were missense, located within its DNA binding domain (DBD) region (74.1%), while other mutations outside of the DBD were frameshift (18.52%) or nonsense mutations (Figs 4B, S13B, and Table S11). Interestingly, *TP53* mutations at the DBD regions, including the most frequent R249S mutation in HCC, were more frequent in HFGC than LFGC tumors (Figs 4B, S13B,E). Moreover, Thai HCC and iCCA cases with *TP53* mutations had a much worse survival than those with wild type p53 (Fig. 4C,D) with comparable results found in TCGA HCC cases (Fig. 4E). Since *TP53* DBD mutations are considered as a gain-of-function mutant p53 protein (mutp53) involved in the maintenance of genome integrity^25–28^, it is plausible that the *TP53* mutations may result in increased genomic instability, leading to elevated FGC scores. Indeed, cases with *TP53* mutation were significantly enriched in HFGC (Fig. 4F and Table S12). Also, patients with *TP53* mutation showed much higher CIN and FGC than wild type p53 (Figs 4G,H, S13C,D,F,G). Moreover, a comprehensive assessment of *TP53* pathway across multiple cancer types demonstrated the influence of *TP53* mutation on genomic stability^29^ and the contribution of genomic instability caused by *TP53* mutation during ovarian cancer chemoresistance and recurrence has been reported^30^. Taken together, we suggest that tumors with higher FGC scores may originate from genomic instability due to p53 mutations, leading to carcinogenesis.

### Association between FGC and Tumor-infiltrating lymphocytes (TILs) in PLC

**Figure 5:**
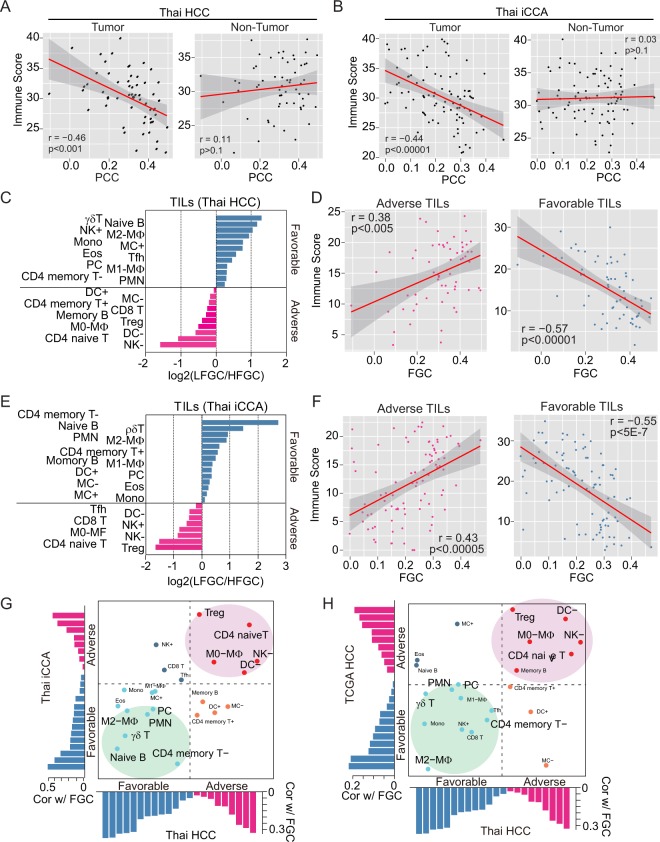
Tumor-infiltrating lymphocytes (TILs) are related to FGC. (**A,B**) The association between PCC and immune score derived from a tumor (left panel of each) or non-tumor (right panel of each) tissue of Thai HCC and Thai iCCA are shown, respectively. (**C–E**) Log2 ratios of the mean of 22 types of TILs in LFGC to HFGC of Thai HCC (**C**) and Thai iCCA (**E**) are shown, respectively. TILs enriched in the LFGC or HFGC group are assigned as favorable or adverse and plotted as either blue or pink bar, respectively. (**D–F**) The association between FGC and the immune score of adverse (left panel) or favorable TILs (right panel) among Thai HCC (**D**) and Thai iCCA (**F**), derived from Fig. 5C,E, are shown, respectively. (**G,H**) Concordance and difference of the associations of FGC with 22 types of TILs between Thai HCC and Thai iCCA (**G**) or Thai HCC and TCGA HCC (**H**) are shown, respectively. The x-axis and y-axis indicate the coefficient estimates from Pearson’s correlation between FGC and the relative proportion of TIL subpopulations in Thai HCC and Y-axis represents for coefficient estimates from Pearson’s correlation between FGC and the relative proportion of TIL subpopulations in Thai iCCA or TCGA HCC. Red and blue bars indicate adverse and favorable associations, predicted by the association with FGC, respectively. Pink or green circled areas indicate TILs commonly associated with FGC among Thai PLC and TCGA HCC.

To analyze the extent of tumor-infiltrating lymphocytes (TILs) in the TIGER-LC cohort, we applied the CIBERSORT (Supplementary Materials and Methods)^31^ and calculated the immune score as the summation of the transformed value of 22 TILs based on the CIBERSORT output (Supplementary Materials and Methods). Consistent with the close association between FGC and immune cytolytic activity, we found a strong inverse correlation between immune scores and FGC in the tumor tissues. However, there was no association in adjacent non-tumor tissues in Thai HCC and iCCA (Fig. 5A,B) and consistent results were found when we analyzed only with samples with high tumor purity (Fig. S14A,B), indicating that the increase in TILs is tumor-specific. In addition, we found that TILs in LFCG were more actively associated with each other compared to HFGC in both iCCA and HCC (Fig. S13C,D). To determine FGC-associated TILs in either HCC or iCCA, we divided TILs into favorable or adverse TILs regarding the enrichment of LFCG or HFGC. Specifically, a subset of favorable TILs is linked to LFGC, while a subset of adverse TILs is linked to HFGC cases (Supplementary Materials and Methods and Fig. 5C,E). Interestingly, we found that immune scores with adverse TILs were positively associated with FGC, while immune score with favorable TILs showed an inverse association with FGC (Fig. 5D,F). This relationship is robust regardless of PLC subtype, indicating that increases in specific TILs may be a consequence of tumor cell-related FGC. Among adverse TILs, Tregs, NK cells and DC cells were consistently and significantly elevated in HFGC from TIGER-LC and TCGA HCC, suggesting that these immune cells may functionally contribute to tumors with an increased FGC (Figs 5G,H and S14E). Currently, a number of immunomodulatory molecules are under investigation for immunotherapy in various cancer^32^. To search for promising candidates of immunotherapy for PLC, we examined the expression profiles of the 54 genes with immunostimulatory or immunoinhibitory function. Most genes showed remarkable features, which were highly upregulated in LFGC but down-regulated in HFGC (Fig. S15A,B, top panels), and showed a negative association with FGC (Fig. S15A,B, bottom panels) in TIGER-LC. Furthermore, several genes expressed much higher in LFGC than those in HFGC (Fig. S15C,D) have been reported to be involved in the development and progression of liver cancer^33–36^. Also, we found that *PDCD1LG2* (PD-L2), a ligand for PD-1, was up-regulated in LFGC compared to HFGC indicative of the sensitivity to the immune-checkpoint inhibitor (ICI)^37^.

### FGC may be useful to predict patients’ responses to ICI-based immunotherapy

Also, to examine whether increased FGC may be associated with ICI response, we analyzed the TCGA melanoma dataset^38^ as liver cancer-related ICI dataset is not available. To infer the FGC level in each patient, we calculated tFA as a surrogate for FGC. Consistently, we found that high tFA was correlated with poor survival in the melanoma cohort (Fig. S15E and Table S13a). Since patients who were treated with anti-CTLA-4 therapy were included in the TCGA melanoma dataset, we performed the Kaplan-Meier (KM) survival analysis by excluding them separately (Supplementary Materials and Methods) and found similar result (Fig. S15F). Besides, the tFA level of the non-responders to the anti-CTLA-4 therapy was much higher compared to responders (Fig. S15G), suggesting that the tFA level was closely associated with the efficacy of anti-CLTA-4 therapy. Similar trends were found in the analysis performed in the cohort of patients with metastatic melanoma who were treated with anti-PD-1 immunotherapy^39^ (Fig. S15H and Table S13b). Overall, these results indicate that FGC could be useful to predict patients’ responses to ICI-based immunotherapy.

## Discussion

PLC is clinically and molecularly heterogeneous and is highly refractory to treatment. The extensive heterogeneity of PLC may be due to the presence of complex etiological factors that causes chronic liver diseases. A common denominator at its origin is a perpetual wound-healing response triggered by parenchymal cell death, with an ensuing inflammatory cascade and concomitant fibrosis progression^40^. In addition, inflammation-induced replication stress promotes DNA damage, which subsequently induces DDR, genomic instability and finally tumorigenesis^41^. Thus, we hypothesized that chronic inflammation may be the main initiator of hepatocarcinogenesis through the induction of genomic instability and aneuploidy. Consistently, *TP53* mutations may be a key driver for inflammatory-mediated hepatocarcinogenesis since it is the most frequently mutated gene in PLC^42^. We found PLC cases with increased FGC enrich for TP53 mutations, consistent with the hypothesis that p53 inactivation may be a trigger to drive chromosome instability and consequently increased ITH. Interestingly, we found that tumors with different FGC scores have different immune cell infiltrates. It is conceivable that the immune surveillance program may help to remove cells with aneuploidy, perhaps by recognizing specific cell surface antigens such as calreticulin, to suppress the growth of cancer cells^43^. However, increased ITH due to extended aneuploidy could overwhelm the immune system, possibly leading to T cell exhaustion^44^. Chronic inflammation may activate several pathways to evade immune surveillance, providing an environment that is inhibitory to productive anti-tumor immune responses. Several types of immunosuppressive cells, such as Tregs, associated with higher grade and poorly differentiated HCC with unfavorable outcomes, may significantly undermine sustained cytotoxicity mediated by T and NK cells, allowing tumor cells to escape immune surveillance^45,46^. Our results indicate that a unique landscape of immune cells is associated with FGC-high tumors, consistent with the central role of immune surveillance in hepatocarcinogenesis.

A recent study showed that aneuploidy of tumor cells adversely affected the immune cell reaction against tumors, suggesting an association of SCNA with immune evasion and the relevance of immunotherapy^47^. In addition, checkpoint inhibitor-based immunotherapies targeting the regulatory pathways of T cells, cytotoxic T-lymphocyte associated antigen 4 (CTLA-4) (ipilimumab) and programmed cell death protein 1 (PD-1) (e.g., nivolumab or pembrolizumab), have enhanced anti-tumor activity with significant clinical benefit in patients with various cancers^48^ including HCC^49^. While it is unclear why most HCC patients do not benefit from immunotherapy, an ineffective immune surveillance program due to immunosuppressive mechanisms that are functional in tumor cells or the tumor-educated liver microenvironment has been suggested. Moreover, it has been difficult to identify predictive biomarkers of response to these agents. Our results show that the FGC score can distinguish TIL subpopulations associated with genomic instability. Therefore, we suggest that an FGC score may be a simple and reliable predictive indicator to stratify patients for immune therapy using only bulk genomic and transcriptomic analysis.

## Methods

### Data sets

A previously described cohort of a set of 398 surgical paired tumor and non-tumor specimens derived from 199 patients of the TIGER-LC cohort (130 iCCA patients and 69 HCC patients) with publicly available Affymetrix Human Transcriptome Array 2.0 data and Affymetrix Genome-Wide Human SNP Nsp/Sty 6.0 data (NCBI GEO accession number GEO: GSE76297 and GSE76213, respectively) were used in this study^4^. Somatic single nucleotide variants and small insertions and deletions among the TIGER-LC cohort were identified based on NCI OncoVar V4, an Agilent SureSelect Custom DNA kit (Agilent Technologies) targeting 2.93 Mb of sequence in 562 genes and publicly accessible (dbGAP (https://www.ncbi.nlm.nih.gov/gap) Accession No. phs001199.v1.p1). For the validation cohort, HCC cohort of 247 Chinese patients from LCI^50^ and TCGA LIHC cohort with 377 HCC patients were used. To validate the association between FGC and immunotherapy with immune checkpoint blockade (ICB), we used transcriptome data from skin cutaneous melanoma datasets derived from TCGA_SKCM^38^ study (n = 472) and metastatic melanoma from Hugo^39^ study (n = 28). Data preprocessing of validation sets were described in the Supplementary Material and Methods.

### Calculation patient correlation coefficient (PCC)

We calculated the patient correlation coefficient based on the pre-selected significantly associated features. To select significantly correlated global features, we calculated the global correlation coefficients and global correlation p-value based on the total transcriptome probes and corresponding genomic segments. Also, MAD of copy number values among the 64,597 transcript probes was calculated. Based on the global correlation p-value and MAD, significantly correlated features of transcriptome probes and corresponding segmented regions were selected by the following cutoff by optimizing stratify patients. For Thai HCC and iCCA, p-value < 0.05 & MAD > 20% of overall distribution; for LCI HCC, p-value < 0.0005 & MAD > 10% for LCI cohort; for TCGA HCC, p-value < 0.01 for TCGA HCC were applied to select for further analysis. Using only features that met p-level and MAD cutoffs, a patient-level correlation was calculated.

$$\mathop{(\begin{array}{c}{c}_{11}{e}_{11}\\ \vdots \\ {c}_{1k}{e}_{1k}\\ \vdots \\ {c}_{1m}{e}_{1m}\end{array})}\limits^{{{\rm{S}}}_{1}}\cdots \mathop{(\begin{array}{c}{c}_{i1}{e}_{{\rm{i}}1}\\ \vdots \\ {c}_{{\rm{i}}k}{e}_{{\rm{i}}k}\\ \vdots \\ {c}_{{\rm{i}}m}{e}_{{\rm{i}}m}\end{array})}\limits^{{{\rm{S}}}_{{\rm{i}}}}\cdots \mathop{(\begin{array}{c}{c}_{{\rm{n}}1}{e}_{{\rm{n}}1}\\ \vdots \\ {c}_{{\rm{n}}k}{e}_{{\rm{n}}k}\\ \vdots \\ {c}_{{\rm{nm}}}{e}_{{\rm{nm}}}\end{array})}\limits^{{{\rm{S}}}_{{\rm{n}}}}$$

Where c is SCNA and e is the expression for feature and n is the n^th^ sample and m is m^th^ feature. The patient-level correlation coefficient (PCC) was calculated based on the selected paired transcriptome probe and the copy-number value of segments for an individual patient.

$$\mathrm{PCC}=\frac{{\sum }_{k=1}^{m}(ck-\bar{c})(ek-\bar{e})}{\sqrt{{\sum }_{k=1}^{m}{(ck-\bar{c})}^{2}{\sum }_{k=1}^{m}{(ek-\bar{e})}^{2}}}$$

Where m is the number of selected features and c is the SCNA value and e is expression value for selected feature and $\bar{{\rm{c}}}$ is the mean of c and $\bar{{\rm{e}}}$ is the mean of e. PCC is the measure of enrichment of the numbers and types of molecular features from genomic instability in an individual sample.

Full methods and any associated references are provided in the Supplementary Information.

## Supplementary information

Supplementary Figures

Supplementary Tables
