# Supplementary material for: Functional Genomic Complexity Defines Intratumor Heterogeneity and Tumor Aggressiveness in Liver Cancer
Source: Sci Rep. 2019 Nov 15;9:16930. doi: 10.1038/s41598-019-52578-8 (PMC6858353; doi:10.1038/s41598-019-52578-8)
Supplement: Supplementary file 2 — Supplementary Tables [file 41598_2019_52578_MOESM2_ESM.pdf]

Supplementary Information for

Functional Genomic Complexity Defines Intratumor Heterogeneity and Tumor Aggressiveness in Liver Cancer

So Mee Kwon[1,2], Anuradha Budhu[1], Hyun Goo Woo[1,2,3], Jittiporn Chaisaingmongkol[1,4,5], Hien Dang[1], Marshonna Forgues[1], Curtis C. Harris[1], Gao Zhang[6], Noam Auslander[7], Eytan Ruppin[7], Chulabhorn Mahidol[4], Mathuros Ruchirawat[4,5], Xin Wei Wang[1,*]

[1]Laboratory of Human Carcinogenesis and Liver Cancer Program, Center for Cancer Research, National Cancer Institute, Bethesda, Maryland 20892, USA; [2]Department of Physiology, Ajou University School of Medicine, Suwon, 16499, Republic of Korea; [3]Department of Biomedical Science, Graduate School, Ajou University, Suwon, 16499, Republic of Korea; [4]Laboratory of Chemical Carcinogenesis, Chulabhorn Research Institute, Bangkok 10210, Thailand; [5]Center of Excellence on Environmental Health and Toxicology, Office of the Higher Education Commission, Ministry of Education, Bangkok 10400, Thailand; [6]Molecular and Cellular Oncogenesis Program and Melanoma Research Center, The Wistar Institute, Philadelphia, PA 19104, USA; [7]Cancer Data Science Lab, National Cancer Institute, National Institute of health, MD 20892, USA

*Corresponding author: xw3u@nih.gov

This PDF file includes:

Tables S1 to S14
- Table S1a. Table for tFA, CIN, GIN and PCC scores among TIGER_LC cohort
- Table S1b. Table for tFA, CIN, and PCC scores for TCGA HCC cohort
- Table S1c. Table for PCC of LCI cohort

Table S2. Overlapped Differentially Expressed Genes among correlated genes between Thai HCC and iCCA
Table S3. Comparison table of overlapped differentially expressed genes among correlated genes.
Table S4. GSEA result compared HFGC with LFGC of Thai iCCA
Table S5. GSEA result compared HFGC with LFGC of Thai HCC
Table S6. GSEA result compared HFGC with LFGC of TCGA HCC
Table S7. Overlapped GSEA result HFGC and LFCG of Thai HCC and Thai iCCA
Table S8. Overlapped recurrent mutation genes between Thai HCC and Thai iCCA
Table S9. Recurrently mutated genes in Thai HCC
Table S10. Recurrently mutated genes in Thai iCCA
Table S11. Information of TP53 mutations mapping.
Table S12. Comparison table of TP53 mutation between HFGC and LFGC among PLC cohorts
Table S13a. Table for tFA of TCGA SKCM cohort
Table S13b. Table for tFA of Hugo Melanoma cohort

Table S1a. Table for tFA, CIN and GIN and PCC scores among TIGER_LC cohort.

Thai HCC								
SampleID	tFA	CIN	GIN	PCC	Group	IHC	ESTIMATE	ABSOULTE
LCS_504A	640.681	16.979	403352065	0.197	Low	1	0.794118	0.48
LCS_505A	778.102	52.261	1451100755	0.380	High	0.9	0.915456	0.24
LCS_506A	802.326	22.908	535560422	0.382	High	0.45	0.751665	0.28
LCS_501A	825.739	31.992	811223027	0.375	High	0.3	0.859835	0.41
LCS_502A	658.129	10.109	229883293	0.342	High	0.4	0.446531	0.48
LCS_503A	713.620	24.450	549670574	0.313	High	1	0.671346	0.93
LCS_557A	769.821	18.055	399433899	0.192	Low	0.97	0.836811	0.19
LCS_565A	598.844	11.442	148924362	0.065	Low	0.35	0.86244	0.18
LCS_541A	785.926	53.129	1356213222	0.353	High	0.95	0.86325	1
LCS_515A	820.240	33.127	758569330	0.372	High	0.9	0.827777	0.23
LCS_530A	918.184	41.706	1135051952	0.487	High	0.95	0.974023	0.81
LCS_550A	757.557	62.330	1758495590	0.332	High	1	0.956787	0.21
LCS_551A	676.774	17.951	344898829	0.183	Low	0.9	0.294253	0.2
LCS_564A	733.027	33.328	811696396	0.403	High	1	0.989249	0.23
LCS_567A	735.945	20.485	534640684	0.243	High	0.9	0.661839	0.19
LCS_507A	839.909	33.276	931279816	0.494	High	0.85	0.856777	0.37
LCS_534A	897.275	47.556	1241731482	0.443	High	0.75	0.999017	0.29
LCS_542A	832.472	58.284	1664585758	0.420	High	1	0.990641	0.2
LCS_517A	703.686	35.660	933585686	0.296	High	1	0.863774	0.79
LCS_518A	624.489	19.425	450936880	0.213	High	0.9	0.813822	0.27
LCS_536A	789.915	13.971	366575577	0.297	High	0.3	0.509022	0.17
LCS_546A	803.054	55.191	1537001449	0.424	High	0.7	0.932417	0.27
LCS_528A	644.484	27.393	699437363	0.255	High	0.95	0.470774	0.18
LCS_512A	714.272	32.523	903640530	0.324	High	0.9	0.944549	0.27
LCS_514A	669.954	24.241	657197541	0.272	High	1	0.991137	0.2
LCS_537A	796.132	14.443	296708063	0.179	Low	0.85	0.47806	0.19
LCS_524A	942.299	49.457	1490817165	0.435	High	0.85	0.896501	0.22
LCS_540A	840.796	60.515	1602868607	0.430	High	1	0.999057	0.27
LCS_511A	724.569	13.700	266709237	0.301	High	0.8	0.741866	0.2
LCS_543A	572.680	9.152	116779742	0.092	Low	1	0.869176	0.2
LCS_547A	873.516	47.273	1141172624	0.440	High	0.85	0.910615	0.27
LCS_533A	620.124	43.539	1078017235	0.135	Low	1	0.455957	NA
LCS_549A	798.784	18.315	431256657	0.302	High	0.85	0.910526	0.24
LCS_510A	664.320	25.297	549797295	0.356	High	1	0.999427	0.24
LCS_519A	772.474	29.342	746753261	0.450	High	1	0.930828	0.37
LCS_529A	646.271	15.923	352397399	0.338	High	1	0.992679	0.44
LCS_568A	776.757	46.241	1113366161	0.371	High	0.75	0.984963	0.19
LCS_508A	895.199	91.113	2545608919	0.453	High	0.9	0.999783	0.25
LCS_513A	716.319	28.509	775289522	0.193	Low	0.5	0.888318	0.25
LCS_523A	645.455	23.448	537544633	0.296	High	0.5	0.979801	0.16

LCS_552A	849.871	42.182	1243360705	0.412	High	0.8	0.732968	0.28
LCS_558A	670.991	15.813	313996006	0.155	Low	1	0.946235	0.22
LCS_538A	574.235	14.355	248098140	0.071	Low	1	0.974766	0.18
LCS_539A	628.682	21.140	481469591	0.320	High	1	0.986288	0.31
LCS_559A	738.192	21.879	452435749	0.377	High	0.98	0.874402	0.22
LCS_516A	696.648	31.652	702750256	0.277	High	1	0.993988	0.18
LCS_520A	769.062	37.655	1367333803	0.340	High	1	0.896512	1
LCS_531A	735.662	62.277	1567398907	0.353	High	0.5	0.997542	0.22
LCS_553A	732.752	38.133	993437434	0.300	High	1	0.958233	0.21
LCS_527A	700.571	16.713	364576441	0.310	High	0.95	0.69855	0.25
LCS_545A	788.601	28.515	699036404	0.386	High	0.98	0.949972	0.26
LCS_532A	780.401	59.329	1436002560	0.411	High	1	0.992218	0.23
LCS_562A	748.455	28.754	653973273	0.314	High	0.9	0.600955	0.24
LCS_566A	618.078	4.784	73026335	0.316	High	0.85	0.347356	0.18
LCS_584A	882.992	43.545	1180203796	0.427	High	0.9	0.990952	0.28
LCS_548A	633.274	13.631	313739762	0.167	Low	1	0.426673	0.19
LCS_544A	839.814	75.377	2057271225	0.409	High	1	0.990868	0.33
LCS_560A	639.351	9.042	203191464	0.087	Low	0.9	0.392243	0.17
LCS_563A	829.761	31.478	825245468	0.410	High	0.98	0.965275	0.71
LCS_509A	711.933	3.624	66725496	-0.107	Low	0.9	0.595722	0.23
LCS_535A	668.365	13.556	415535629	0.009	Low	0.3	0.153153	0.19
LCS_525A	687.470	26.698	615907314	0.241	High	1	0.94833	0.22
**Thai iCCA**								
**SampleID**	**tFA**	**CIN**	**GIN**	**PCC**	**Group**	**IHC**	**ESTIMATE**	**ABSOULTE**
LCS_597A	678.519	11.454	207581893	0.220	High	0.3	0.471148	0.17
LCS_623A	773.185	32.819	728917626	0.342	High	0.9	0.577374	0.21
LCS_639A	609.062	22.902	751880436	0.092	Low	0.6	0.520354	NA
LCS_657A	713.145	58.278	1549290066	0.295	High	0.75	0.791191	0.22
LCS_658A	703.025	54.643	1672422572	0.309	High	0.8	0.986366	0.25
LCS_580A	696.278	35.215	914233942	0.262	High	0.6	0.707433	0.21
LCS_600A	684.673	33.155	942935434	0.265	High	0.2	0.666849	0.2
LCS_616A	667.434	37.352	1116986930	0.314	High	0.8	0.983009	0.2
LCS_637A	605.441	5.480	56828896	0.089	Low	0.4	0.572446	0.41
LCS_650A	587.171	2.256	46431713	0.102	Low	0.7	0.349039	0.2
LCS_651A	630.054	46.268	1211621507	0.225	High	0.8	0.287086	0.24
LCS_586A	647.809	34.884	1088688021	0.273	High	0.8	0.854036	0.34
LCS_587A	761.435	58.814	1589823122	0.336	High	0.6	0.948613	0.42
LCS_595A	773.857	46.443	1340567279	0.470	High	0.75	0.944374	0.24
LCS_645A	697.373	56.373	1545821617	0.348	High	0.9	0.89388	0.21
LCS_570A	628.549	54.049	1446895584	0.153	Low	0.85	0.471128	NA
LCS_592A	757.727	24.828	623836669	0.288	High	0.36	0.315534	0.22
LCS_603A	776.387	49.256	1542412037	0.319	High	1	0.98112	NA
LCS_684A	626.820	20.176	488344233	0.150	Low	0.6	0.568633	0.18
LCS_643A	577.480	6.113	137429367	0.065	Low	0.4	0.020751	0.23

LCS_601A	580.850	6.497	109161290	0.086	Low	0.4	0.052547	0.2
LCS_612A	635.219	29.895	744391961	0.282	High	0.7	0.606239	0.19
LCS_653A	615.784	30.508	962264877	0.148	Low	0.1	0.267743	0.17
LCS_578A	659.671	38.443	992595901	0.294	High	0.5	0.483882	0.23
LCS_618A	649.149	26.224	600305945	0.251	High	0.3	0.603252	0.23
LCS_573A	769.858	30.096	829564824	-0.002	Low	0.3	0.826921	0.21
LCS_681A	682.591	41.728	1100954844	0.298	High	0.65	0.834593	0.24
LCS_575A	590.652	13.258	228833363	0.196	Low	0.9	0.963525	0.24
LCS_583A	687.135	53.495	1495049221	0.295	High	0.8	0.903164	0.25
LCS_614A	495.974	10.878	281157656	0.053	Low	0.6	0.579085	0.2
LCS_634A	674.090	15.298	409466797	0.240	High	0.6	0.428643	0.18
LCS_664A	732.907	47.914	1409675866	0.314	High	0.5	0.931449	0.28
LCS_596A	705.811	67.105	1823564599	0.303	High	1	0.926517	0.43
LCS_598A	722.202	52.126	1417752222	0.362	High	0.9	0.998949	0.24
LCS_644A	703.386	44.953	1103603216	0.340	High	0.8	0.879404	0.21
LCS_646A	784.935	54.046	1530370900	0.336	High	0.66	0.782799	0.2
LCS_674A	594.768	44.826	1064451077	0.188	Low	0.7	0.626914	0.28
LCS_581A	718.540	19.636	385201627	0.154	Low	0.35	0.749788	0.16
LCS_626A	751.591	50.648	1327498421	0.335	High	0.7	0.575841	0.28
LCS_668A	566.749	17.829	407810843	0.111	Low	0.7	0.4864	0.25
LCS_585A	601.376	3.536	55822698	0.091	Low	0.2	0.295896	0.19
LCS_602A	631.518	16.423	379376446	0.081	Low	0.15	0.177136	0.17
LCS_606A	518.923	11.716	325891482	0.019	Low	0.95	0.93018	0.17
LCS_621A	649.324	19.102	434878013	0.124	Low	0.45	0.487482	0.19
LCS_615A	653.261	30.296	697463598	0.246	High	1	0.5153	0.25
LCS_631A	629.553	49.979	1333334600	0.257	High	0.74	0.774045	0.22
LCS_675A	638.788	13.624	264312428	0.166	Low	0.2	0.55685	0.18
LCS_683A	594.087	19.823	604693582	0.164	Low	0.5	0.560874	0.23
LCS_687A	799.126	35.146	1015587242	0.297	High	0.78	0.898884	0.2
LCS_636A	577.275	11.827	438127775	0.128	Low	0.2	0.582407	0.19
LCS_574A	728.821	43.278	1198571020	0.394	High	0.85	0.947743	0.24
LCS_624A	666.230	3.808	89675379	0.149	Low	0.8	0.366859	0.17
LCS_688A	684.929	31.601	823879909	0.255	High	0.6	0.743568	0.18
LCS_608A	775.442	98.621	2712928645	0.390	High	1	0.997976	0.76
LCS_610A	707.752	47.889	1269993472	0.336	High	0.9	0.998666	0.18
LCS_625A	715.527	51.786	1464373282	0.285	High	0.85	0.877041	0.22
LCS_590A	658.820	32.303	716888550	0.309	High	0.96	0.980892	0.22
LCS_638A	704.271	31.870	796184399	0.315	High	0.8	0.949938	0.48
LCS_642A	594.571	10.872	161118644	0.045	Low	0.5	0.569433	0.22
LCS_670A	522.376	17.844	536832767	0.013	Low	0.1	0.244029	0.17
LCS_572A	657.343	51.279	1299208549	0.330	High	0.7	0.664094	0.21
LCS_577A	688.471	30.125	619840636	0.172	Low	0.3	0.908091	0.25
LCS_671A	685.355	16.495	343322234	0.234	High	0.8	0.463769	0.16
LCS_571A	686.204	38.836	1159268010	0.233	High	0.7	0.992153	0.28
LCS_599A	640.682	23.899	615559995	0.260	High	0.5	0.976876	0.19

LCS_604A	707.234	19.668	560808338	0.237	High	1	0.856809	0.16
LCS_613A	548.419	4.191	88028559	0.077	Low	0.1	NA	0.19
LCS_667A	630.378	3.064	61966037	0.007	Low	0.3	0.964846	0.18
LCS_678A	564.741	11.787	224174294	0.045	Low	0.25	NA	0.17
LCS_635A	634.352	17.341	385399633	0.192	Low	0.95	0.4821	0.19
LCS_641A	593.070	7.808	96131018	0.063	Low	0.35	0.378206	0.2
LCS_677A	778.054	31.913	853621266	0.382	High	0.55	0.903748	0.21
LCS_579A	589.743	16.307	341832625	0.129	Low	0.85	0.309957	0.17
LCS_620A	712.323	55.379	1723498332	0.286	High	0.5	0.968865	0.2
LCS_628A	751.489	7.155	215624381	0.198	Low	0.4	0.661882	0.16
LCS_630A	759.285	30.049	818562120	0.225	High	0.5	0.151957	0.27
LCS_680A	787.884	63.929	1691733143	0.195	Low	0.75	0.60259	NA
LCS_633A	611.311	12.287	170765978	0.150	Low	0.45	0.26487	0.16
LCS_640A	732.058	28.958	754347362	0.269	High	1	0.774163	0.2
LCS_649A	725.227	30.076	848795702	0.323	High	0.5	0.681162	0.19
LCS_685A	596.239	22.752	578102989	0.202	High	0.8	0.786366	0.21
LCS_594A	668.579	34.094	931371596	0.288	High	0.4	0.89562	0.21
LCS_627A	643.123	30.926	729128138	0.283	High	0.8	0.936532	0.18
LCS_648A	638.022	24.899	634005244	0.209	High	0.65	0.9869	0.22
LCS_654A	563.573	20.772	462634683	0.006	Low	0.35	0.426095	0.18
LCS_679A	681.193	20.499	592245521	0.231	High	0.57	0.362788	0.2
LCS_609A	646.746	26.012	649514826	0.172	Low	0.85	0.599898	0.19
LCS_652A	731.838	51.120	1344823155	0.288	High	0.9	0.953952	0.24
LCS_669A	647.614	20.800	506482564	0.158	Low	0.4	0.949991	0.19
LCS_682A	695.584	34.073	981227061	0.304	High	0.6	0.991582	0.28

Table S1b. Table for tFA, CIN and PCC for TCGA HCC cohort

SampleID	tFA	CIN	PCC	Group	IHC	ESTIMATE	ABSOULTE
TCGA-DD-AACO-01A-11R-A41C-07	260.416	31.593	0.188	Low	0.8	0.84868	0.2
TCGA-CC-5263-01A-01R-A131-07	231.347	32.143	0.278	High	0.8	0.8827185	0.69
TCGA-CC-5261-01A-01R-A131-07	270.961	45.330	0.314	High	0.77	0.7769084	0.29
TCGA-CC-A5UC-01A-11R-A28V-07	236.177	22.802	0.231	High	0.75	0.8717824	0.25
TCGA-RC-A6M5-01A-11R-A32O-07	233.243	1.648	0.153	Low	0.69	0.8203688	0.27
TCGA-DD-A1EH-01A-11R-A131-07	287.457	23.901	0.314	High	0.85	0.9061454	0.46
TCGA-ED-A8O5-01A-11R-A36F-07	314.650	25.824	0.347	High	1	0.9275228	0.83
TCGA-CC-5264-01A-01R-A131-07	266.629	69.780	0.335	High	0.9	0.9246758	0.56
TCGA-DD-AACS-01A-11R-A41C-07	273.232	41.484	0.322	High	0.9	0.9529532	0.88
TCGA-G3-A5SL-01A-11R-A27V-07	275.664	17.582	0.330	High	0.9	0.9118005	0.35
TCGA-DD-AACE-01A-11R-A41C-07	291.702	56.044	0.327	High	0.93	0.9110346	0.26
TCGA-BC-4072-01B-11R-A155-07	235.645	27.198	0.235	High	0.95	0.7930974	0.44
TCGA-CC-A7II-01A-11R-A33J-07	298.547	44.780	0.259	High	1	0.9441255	0.26
TCGA-DD-AAEK-01A-11R-A41C-07	256.899	37.363	0.322	High	1	0.7594022	0.25
TCGA-DD-AACB-01A-11R-A41C-07	267.812	48.901	0.281	High	0.85	0.8191516	0.2
TCGA-EP-A3JL-01A-11R-A213-07	257.045	13.462	0.273	High	0.85	0.7694098	0.2
TCGA-EP-A2KB-01A-11R-A180-07	304.423	56.319	0.303	High	0.97	0.939486	0.22
TCGA-ED-A4XI-01A-11R-A266-07	226.076	5.769	0.117	Low	0.8	0.7678172	0.3
TCGA-G3-A7M7-01A-12R-A352-07	246.379	3.297	0.175	Low	0.7	0.9253445	0.54
TCGA-CC-A7IG-01A-11R-A33J-07	262.417	55.769	0.267	High	1	0.9028167	0.26
TCGA-DD-AACT-01A-11R-A41C-07	289.843	28.022	0.331	High	1	0.8300936	0.21
TCGA-UB-A7MF-01A-11R-A33J-07	273.117	22.802	0.237	High	0.85	0.771617	0.24
TCGA-HP-A5N0-01A-11R-A28V-07	261.943	17.582	0.341	High	1	0.8128798	0.45
TCGA-CC-5260-01A-01R-A131-07	260.224	27.747	0.203	High	0.8	0.8176256	0.47
TCGA-G3-A25X-01A-11R-A16W-07	266.378	37.912	0.279	High	0.65	0.7645695	1
TCGA-DD-AAE9-01A-11R-A41C-07	264.033	34.615	0.270	High	0.97	0.9485952	0.29
TCGA-RC-A7SF-01A-11R-A352-07	236.807	18.681	0.330	High	0.87	0.9134668	0.58
TCGA-NI-A8LF-01A-11R-A36F-07	262.422	47.253	0.345	High	0.9	0.8897808	0.19
TCGA-2Y-A9H6-01A-11R-	267.611	21.429	0.280	High	0.8	0.78893	0.49

A39D-07						5	
TCGA-DD-AAD6-01A-11R-A41C-07	272.579	27.198	0.326	High	0.92	0.9512281	0.87
TCGA-ZP-A9D2-01A-11R-A38B-07	256.995	31.044	0.314	High	1	0.8894523	0.25
TCGA-DD-A4NQ-01A-21R-A28V-07	232.242	32.143	0.304	High	0.8	0.8960213	0.39
TCGA-NI-A4U2-01A-11R-A28V-07	253.876	16.484	0.194	Low	1	0.8890737	0.67
TCGA-ED-A97K-01A-21R-A38B-07	255.413	34.066	0.224	High	0.5	0.8580838	0.28
TCGA-DD-A4NB-01A-12R-A266-07	213.755	8.516	0.153	Low	0.9	0.707266	0.29
TCGA-2Y-A9H7-01A-11R-A39D-07	255.019	15.934	0.273	High	0.85	0.9293143	0.79
TCGA-CC-A3MC-01A-11R-A22L-07	247.075	18.407	0.322	High	0.75	0.8824604	0.24
TCGA-BC-A10Q-01A-11R-A131-07	268.860	70.055	0.193	Low	0.97	0.9000269	0.46
TCGA-MI-A75E-01A-11R-A32O-07	224.774	13.736	0.177	Low	0.73	0.8196875	0.34
TCGA-WX-AA46-01A-11R-A39D-07	253.677	6.593	0.151	Low	0.85	0.8614018	0.19
TCGA-FV-A3I1-01A-11R-A22L-07	263.288	25.824	0.287	High	1	0.8273036	0.3
TCGA-DD-A4NJ-01A-11R-A27V-07	260.172	27.473	0.317	High	0.5	0.8128995	0.25
TCGA-DD-AAVY-01A-11R-A41C-07	287.682	17.857	0.283	High	0.85	0.952509	0.64
TCGA-DD-AACK-01A-11R-A41C-07	228.015	3.022	0.122	Low	0.8	0.9274937	0.57
TCGA-DD-AAE2-01A-11R-A41C-07	232.749	13.187	0.249	High	1	0.8116323	0.38
TCGA-XR-A8TF-01A-11R-A36F-07	297.783	53.297	0.279	High	0.8	0.9401696	0.41
TCGA-KR-A7K8-01A-11R-A33J-07	249.926	18.681	0.252	High	1	0.7513417	1
TCGA-DD-A118-01A-11R-A131-07	255.236	47.253	0.246	High	0.95	0.8821516	0.52
TCGA-BD-A3EP-01A-11R-A22L-07	231.000	34.341	0.278	High	1	0.7375086	0.21
TCGA-KR-A7K2-01A-12R-A33R-07	276.767	58.791	0.267	High	1	0.8597256	0.28
TCGA-ED-A7XO-01A-11R-A352-07	305.880	20.330	0.309	High	0.3	0.8325818	0.18
TCGA-DD-A4NF-01A-11R-A27V-07	259.792	4.945	0.130	Low	0.9	0.9381397	0.76
TCGA-ZS-A9CG-01A-11R-A37K-07	232.120	9.890	0.214	High	0.85	0.9095129	0.2
TCGA-EP-A3RK-01A-11R-A22L-07	241.578	18.956	0.283	High	0.75	0.7230537	0.32
TCGA-DD-A1EK-01A-11R-A213-07	248.430	11.538	0.244	High	0.75	0.8377221	0.3
TCGA-WJ-A86L-01A-12R-A39D-07	255.149	20.879	0.241	High	0.8	0.9524147	0.44
TCGA-DD-A1ED-01A-11R-A155-07	226.657	0.275	0.016	Low	1	0.7923279	0.24
TCGA-G3-AAV3-01A-11R-A37K-07	236.328	9.615	0.226	High	0.75	0.7929168	0.44
TCGA-2Y-A9H5-01A-11R-	284.378	25.824	0.311	High	0.75	0.87138	0.23

A38B-07						15	
TCGA-G3-A7M8-01A-11R-A33R-07	224.294	0.000	-0.013	Low	0.8	0.8696565	0.48
TCGA-DD-A4NK-01A-11R-A28V-07	282.209	20.604	0.283	High	0.9	0.9147014	0.21
TCGA-DD-A39X-01A-11R-A213-07	256.587	12.637	0.321	High	0.9	0.8668455	0.2
TCGA-DD-A39Z-01A-11R-A213-07	284.736	29.670	0.233	High	1	0.9595869	0.61
TCGA-DD-AAD2-01A-11R-A41C-07	241.354	9.341	0.269	High	0.42	0.7697482	0.35
TCGA-DD-AAE7-01A-11R-A41C-07	213.623	5.220	0.173	Low	1	0.8294046	0.6
TCGA-BC-A216-01A-11R-A155-07	255.448	23.352	0.263	High	0.94	0.8823803	0.36
TCGA-DD-A11A-01A-11R-A131-07	261.165	57.967	0.334	High	0.85	0.9419613	0.2
TCGA-CC-A8HV-01A-11R-A36F-07	301.973	69.505	0.337	High	1	0.9560854	0.24
TCGA-G3-AAV4-01A-11R-A38B-07	305.411	31.868	0.247	High	1	0.9266231	0.21
TCGA-DD-A4NP-01A-11R-A28V-07	255.375	7.143	0.140	Low	1	0.9033545	0.48
TCGA-ED-A459-01A-11R-A266-07	270.033	39.286	0.357	High	1	0.887356	0.22
TCGA-DD-AADV-01A-11R-A39D-07	287.640	31.319	0.300	High	1	0.885392	0.21
TCGA-DD-AADF-01A-11R-A41C-07	295.856	45.604	0.253	High	0.8	0.9231742	0.35
TCGA-2Y-A9H8-01A-11R-A39D-07	275.156	55.769	0.298	High	0.65	0.8682814	0.43
TCGA-DD-A113-01A-11R-A131-07	240.990	64.286	0.314	High	0.98	0.8858781	0.2
TCGA-G3-AAV7-01A-11R-A38B-07	264.372	48.626	0.318	High	0.88	0.8600343	0.32
TCGA-CC-A8HT-01A-11R-A36F-07	246.263	25.000	0.275	High	1	0.896248	0.32
TCGA-G3-A3CK-01A-11R-A213-07	255.166	10.989	0.180	Low	1	0.8725092	0.23
TCGA-DD-A73D-01A-12R-A32O-07	286.004	35.440	0.301	High	0.98	0.9747933	0.26
TCGA-RC-A7SK-01A-11R-A352-07	281.033	26.374	0.292	High	1	0.9533785	0.21
TCGA-DD-AAEB-01A-11R-A41C-07	270.490	15.110	0.147	Low	1	0.9035715	0.91
TCGA-ZS-A9CD-01A-11R-A37K-07	245.998	10.989	0.213	High	0.8	0.8452839	0.22
TCGA-DD-A4NN-01A-11R-A28V-07	301.580	39.011	0.347	High	1	0.9415511	0.65
TCGA-T1-A6J8-01A-11R-A32O-07	246.107	23.901	0.330	High	1	0.8912187	0.26
TCGA-FV-A4ZP-01A-12R-A266-07	269.774	50.549	0.226	High	0.8	0.833488	0.21
TCGA-DD-AADM-01A-11R-A41C-07	258.412	31.593	0.285	High	0.85	0.8821861	0.43
TCGA-BC-A10Z-01A-11R-A131-07	276.224	25.549	0.238	High	0.9	0.9771814	0.41
TCGA-BW-A5NO-01A-11R-A27V-07	282.413	54.396	0.321	High	1	0.8893121	0.22
TCGA-DD-AADA-01A-11R-	279.322	26.648	0.289	High	0.8	0.81477	0.69

A41C-07						77	
TCGA-DD-A3A4-01A-11R-A22L-07	272.748	7.692	0.120	Low	1	0.94309 23	0.51
TCGA-DD-AAW2-01A-11R-A41C-07	280.589	23.901	0.318	High	0.85	0.93089 34	0.19
TCGA-DD-AAC8-01A-11R-A41C-07	278.117	45.330	0.291	High	0.8	0.90386 69	0.57
TCGA-DD-A119-01A-11R-A131-07	261.286	29.945	0.294	High	0.9	0.87695 98	0.29
TCGA-DD-A3A3-01A-11R-A22L-07	287.410	19.231	0.247	High	1	0.94008	0.2
TCGA-DD-AACQ-01A-11R-A41C-07	260.112	19.505	0.279	High	1	0.97014 22	0.89
TCGA-BC-A110-01A-11R-A131-07	238.159	0.000	0.021	Low	0.55	0.67043 94	0.59
TCGA-DD-AACU-01A-11R-A41C-07	262.727	77.473	0.334	High	1	0.89201 76	0.24
TCGA-PD-A5DF-01A-11R-A27V-07	218.277	18.407	0.264	High	1	0.78544 98	0.32
TCGA-DD-A3A8-01A-11R-A22L-07	267.811	13.187	0.237	High	0.9	0.94297 22	0.38
TCGA-K7-A6G5-01A-11R-A311-07	253.425	7.967	0.195	Low	1	0.83918 36	0.59
TCGA-LG-A9QD-01A-11R-A38B-07	246.293	9.890	0.200	Low	0.96	0.89014 23	0.3
TCGA-EP-A2KC-01A-11R-A213-07	267.252	39.011	0.330	High	1	0.91269 7	0.23
TCGA-DD-AADD-01A-11R-A41C-07	294.868	41.209	0.267	High	0.9	0.95746 51	0.43
TCGA-FV-A4ZQ-01A-11R-A266-07	244.964	26.374	0.248	High	0.75	0.80832 15	0.16
TCGA-BC-A5W4-01A-11R-A28V-07	278.064	79.670	0.308	High	0.9	0.97482 84	0.38
TCGA-ED-A82E-01A-11R-A352-07	266.591	62.637	0.215	High	0.4	0.87454 27	0.24
TCGA-BC-A10U-01A-11R-A131-07	264.661	28.022	0.332	High	0.96	0.94331 86	0.8
TCGA-BD-A3ER-01A-11R-A213-07	234.864	7.692	0.180	Low	1	0.81354 02	0.18
TCGA-DD-AAVV-01A-11R-A41C-07	249.730	35.440	0.335	High	0.75	0.74623 39	0.21
TCGA-BC-A3KG-01A-11R-A213-07	307.723	35.165	0.307	High	1	0.95004 71	0.22
TCGA-FV-A2QR-01A-11R-A213-07	235.086	26.099	0.293	High	0.7	0.86784 49	0.79
TCGA-DD-AAC9-01A-11R-A41C-07	247.083	9.615	0.197	Low	0.8	0.77435 96	0.42
TCGA-DD-A4NI-01A-11R-A27V-07	251.822	17.857	0.264	High	0.95	0.80047 6	0.17
TCGA-5C-AAPD-01A-21R-A39D-07	297.216	46.703	0.317	High	0.53	0.75875 71	0.28
TCGA-MI-A75I-01A-11R-A32O-07	282.767	27.747	0.359	High	0.89	0.93422 02	0.57
TCGA-FV-A3R2-01A-11R-A22L-07	284.317	37.912	0.376	High	1	0.92558 92	0.77
TCGA-DD-A1EL-01A-11R-A155-07	256.835	41.758	0.312	High	0.9	0.93918 24	0.19
TCGA-DD-A1EA-01A-11R-A131-07	255.273	15.385	0.220	High	1	0.87922 71	0.28
TCGA-DD-A73B-01A-12R-	285.105	67.033	0.342	High	0.96	0.95000	0.5

A32O-07						4	
TCGA-G3-A25S-01A-11R-A16W-07	271.510	53.297	0.360	High	1	0.8985935	0.2
TCGA-UB-AA0U-01A-11R-A38B-07	256.133	35.165	0.332	High	1	0.8057393	0.33
TCGA-FV-A3I0-01A-11R-A22L-07	274.356	32.692	0.249	High	1	0.8768731	0.23
TCGA-FV-A23B-01A-11R-A16W-07	290.434	26.099	0.315	High	0.9	0.8799833	0.74
TCGA-2V-A95S-01A-11R-A37K-07	272.212	19.780	0.208	High	0.5	0.7218246	0.51
TCGA-DD-AADL-01A-11R-A41C-07	291.173	38.462	0.351	High	0.9	0.9565283	0.21
TCGA-CC-5258-01A-01R-A131-07	262.818	28.571	0.295	High	0.9	0.8662359	0.22
TCGA-EP-A12J-01A-11R-A131-07	275.494	12.912	0.238	High	0.93	0.9194492	0.79
TCGA-5R-AA1C-01A-11R-A41C-07	272.862	30.220	0.267	High	0.94	0.9049292	0.26
TCGA-DD-A3A6-01A-11R-A22L-07	262.330	0.000	0.017	Low	1	0.6903289	0.2
TCGA-DD-AADC-01A-11R-A41C-07	259.797	40.110	0.367	High	0.75	0.9445051	0.2
TCGA-DD-A3A1-01A-11R-A213-07	247.331	27.473	0.284	High	1	0.8897118	0.71
TCGA-DD-AACA-01A-11R-A41C-07	279.834	22.802	0.231	High	0.8	0.9403974	0.85
TCGA-DD-A4NA-01A-11R-A266-07	277.137	25.275	0.170	Low	1	0.8490168	0.27
TCGA-MI-A75H-01A-11R-A32O-07	249.304	10.989	0.257	High	0.83	0.8339857	0.61
TCGA-CC-5262-01A-01R-A131-07	231.546	16.484	0.206	High	0.8	0.7638443	0.34
TCGA-DD-AAE3-01A-11R-A41C-07	257.778	34.341	0.253	High	1	0.8554586	0.28
TCGA-2Y-A9H4-01A-11R-A38B-07	252.345	20.604	0.261	High	0.85	0.8880508	0.19
TCGA-DD-A3A7-01A-11R-A22L-07	271.264	50.000	0.267	High	1	0.9275157	0.3
TCGA-DD-AAEH-01A-11R-A41C-07	247.020	17.033	0.288	High	1	0.7626037	0.71
TCGA-FV-A496-01A-11R-A266-07	293.555	44.231	0.306	High	0.85	0.9655473	0.32
TCGA-G3-AAV2-01A-11R-A37K-07	278.249	16.484	0.223	High	0.85	0.9151682	0.38
TCGA-MI-A75G-01A-11R-A32O-07	298.360	38.462	0.269	High	0.87	0.9488657	0.25
TCGA-DD-A1EF-01A-11R-A131-07	286.839	33.242	0.231	High	0.85	0.8751279	0.29
TCGA-GJ-A3OU-01A-31R-A38B-07	217.999	12.912	0.225	High	1	0.4823377	0.25
TCGA-GJ-A9DB-01A-11R-A37K-07	230.459	8.242	0.231	High	0.65	0.8583934	0.32
TCGA-DD-A11D-01A-11R-A131-07	254.002	18.956	0.206	High	0.75	0.9042465	0.38
TCGA-CC-A1HT-01A-11R-A131-07	256.159	19.231	0.229	High	0.6	0.6681142	0.21
TCGA-DD-AADW-01A-11R-A39D-07	285.979	36.538	0.316	High	1	0.9196176	0.25
TCGA-CC-A9FU-01A-11R-	301.514	63.187	0.301	High	0.84	0.96679	0.21

A37K-07						99	
TCGA-ED-A5KG-01A-11R-A27V-07	257.727	0.275	0.185	Low	1	0.447413	0.36
TCGA-DD-AAEE-01A-11R-A41C-07	263.540	31.868	0.340	High	1	0.939832	0.24
TCGA-WX-AA47-01A-11R-A39D-07	257.448	13.736	0.143	Low	0.95	0.9635843	0.22
TCGA-BC-A10S-01A-22R-A131-07	272.285	9.890	0.229	High	0.9	0.7793755	0.62
TCGA-G3-A3CJ-01A-11R-A213-07	261.481	16.484	0.198	Low	1	0.9106893	0.58
TCGA-DD-A39Y-01A-11R-A213-07	257.197	45.879	0.298	High	0.9	0.9425404	0.3
TCGA-FV-A495-01A-11R-A266-07	281.040	26.099	0.161	Low	0.7	0.7588298	0.49
TCGA-DD-AACP-01A-11R-A41C-07	265.545	52.473	0.276	High	0.7	0.9728134	0.21
TCGA-DD-A4NR-01A-11R-A311-07	229.735	4.670	0.221	High	0.7	0.6352681	0.42
TCGA-DD-AADP-01A-11R-A39D-07	241.814	26.923	0.384	High	1	0.8866511	0.23
TCGA-DD-AAD5-01A-11R-A41C-07	249.788	52.473	0.232	High	0.94	0.8779055	0.21
TCGA-RC-A7SH-01A-11R-A38B-07	318.990	35.165	0.281	High	0.65	0.955381	0.33
TCGA-DD-AAD8-01A-11R-A41C-07	282.463	76.374	0.324	High	0.83	0.9248925	0.24
TCGA-DD-AAVP-01A-11R-A41C-07	268.066	18.132	0.250	High	0.8	0.9055765	0.43
TCGA-DD-AACI-01A-11R-A41C-07	274.140	36.264	0.283	High	0.85	0.7255949	0.25
TCGA-DD-A114-01A-11R-A131-07	268.878	9.341	0.238	High	0.75	0.6847981	0.22
TCGA-2Y-A9GW-01A-11R-A38B-07	254.905	9.066	0.307	High	1	0.7481811	0.2
TCGA-DD-A116-01A-11R-A131-07	278.716	21.978	0.262	High	0.9	0.8873743	0.22
TCGA-DD-AACW-01A-11R-A41C-07	283.124	23.352	0.335	High	1	0.9320209	0.22
TCGA-DD-A39W-01A-11R-A213-07	254.580	9.890	0.137	Low	0.75	0.9316022	0.18
TCGA-2Y-A9GU-01A-11R-A38B-07	259.268	32.143	0.235	High	1	0.9361984	0.74
TCGA-CC-A9FV-01A-11R-A37K-07	307.515	0.000	-0.009	Low	0.94	0.523932	0.37
TCGA-DD-AAEG-01A-11R-A39D-07	260.499	22.527	0.340	High	1	0.9393854	0.39
TCGA-DD-AAW0-01A-11R-A41C-07	254.335	29.121	0.376	High	0.85	0.7869982	0.48
TCGA-2Y-A9H0-01A-11R-A38B-07	255.371	22.527	0.314	High	1	0.8947486	0.83
TCGA-DD-AACG-01A-11R-A41C-07	269.025	39.560	0.333	High	0.85	0.9083648	0.23
TCGA-RG-A7D4-01A-12R-A33R-07	279.351	47.253	0.333	High	0.65	0.8439542	0.23
TCGA-ZP-A9D0-01A-11R-A37K-07	266.361	16.484	0.281	High	0.95	0.9233924	0.68
TCGA-DD-AAVX-01A-11R-A41C-07	243.246	10.989	0.170	Low	0.75	0.8377109	0.69
TCGA-2Y-A9GS-01A-12R-	257.149	27.473	0.320	High	0.65	0.86388	0.3

A38B-07						68	
TCGA-XR-A8TG-01A-11R-A36F-07	250.933	21.703	0.266	High	0.7	0.8813241	0.26
TCGA-ZP-A9CZ-01A-11R-A38B-07	225.622	22.253	0.290	High	0.8	0.8077429	0.66
TCGA-DD-A1EC-01A-21R-A131-07	241.721	21.429	0.140	Low	1	0.6560445	0.63
TCGA-ED-A8O6-01A-11R-A36F-07	269.299	52.747	0.280	High	1	0.9315024	0.31
TCGA-G3-A3CH-01A-11R-A22L-07	250.513	14.011	0.231	High	1	0.8559659	0.32
TCGA-EP-A26S-01A-11R-A16W-07	277.035	11.813	0.162	Low	0.95	0.9397402	0.19
TCGA-BC-A69I-01A-11R-A311-07	264.234	12.088	0.220	High	0.85	0.8558551	0.2
TCGA-RC-A7S9-01A-11R-A33R-07	253.401	19.231	0.249	High	0.9	0.9150451	0.4
TCGA-BW-A5NQ-01A-11R-A27V-07	256.292	35.165	0.249	High	1	0.8345288	0.29
TCGA-CC-A8HU-01A-11R-A36F-07	310.050	48.077	0.362	High	0.9	0.9516019	0.35
TCGA-BC-A3KF-01A-11R-A213-07	274.568	35.714	0.391	High	1	0.9196009	0.25
TCGA-DD-A4NO-01A-11R-A28V-07	268.805	65.110	0.344	High	1	0.8810145	0.23
TCGA-ED-A7PZ-01A-11R-A33R-07	298.805	60.989	0.330	High	1	0.9753989	0.26
TCGA-DD-AAE0-01A-11R-A41C-07	294.683	53.297	0.327	High	0.37	0.9147257	0.35
TCGA-LG-A9QC-01A-11R-A37K-07	273.416	30.495	0.280	High	0.95	0.9435021	0.22
TCGA-DD-AACD-01A-11R-A41C-07	259.914	16.209	0.230	High	0.83	0.8277659	0.76
TCGA-BC-A217-01A-11R-A155-07	293.566	51.923	0.340	High	0.96	0.9040577	0.19
TCGA-5C-A9VH-01A-11R-A37K-07	247.693	18.681	0.289	High	1	0.8836631	0.68
TCGA-G3-A25Z-01A-11R-A16W-07	239.946	38.462	0.282	High	1	0.8709873	0.21
TCGA-CC-A3MA-01A-11R-A213-07	260.424	37.637	0.264	High	0.85	0.9046929	0.2
TCGA-UB-A7MC-01A-11R-A33R-07	290.010	45.055	0.325	High	0.9	0.9630525	0.23
TCGA-ZP-A9CV-01A-11R-A38B-07	279.904	10.165	0.248	High	1	0.8093159	0.67
TCGA-BD-A2L6-01A-11R-A213-07	273.637	18.407	0.237	High	0.9	0.8966922	0.7
TCGA-GJ-A6C0-01A-12R-A311-07	260.885	23.626	0.278	High	0.8	0.8270396	0.34
TCGA-DD-AAW1-01A-11R-A41C-07	246.586	10.714	0.185	Low	0.85	0.9310221	0.25
TCGA-DD-AADB-01A-11R-A41C-07	259.629	31.319	0.333	High	0.9	0.8511496	0.22
TCGA-UB-A7MD-01A-12R-A352-07	257.889	18.132	0.242	High	0.8	0.8214418	0.42
TCGA-2Y-A9HA-01A-11R-A39D-07	260.797	21.154	0.322	High	0.89	0.9344875	0.22
TCGA-ZP-A9D1-01A-11R-A38B-07	248.282	49.725	0.283	High	0.75	0.8382772	0.26
TCGA-BC-4073-01B-02R-	224.987	8.516	0.146	Low	0.7	0.70859	0.51

A131-07						51	
TCGA-G3-A7M9-01A-23R-A352-07	253.649	48.077	0.242	High	0.6	0.9703139	0.3
TCGA-RC-A6M6-01A-11R-A32O-07	235.672	23.077	0.261	High	0.62	0.952681	0.29
TCGA-CC-A7IH-01A-11R-A33J-07	278.554	17.582	0.251	High	1	0.9441917	0.24
TCGA-DD-A4NH-01A-11R-A27V-07	260.406	35.440	0.356	High	0.75	0.8471844	0.31
TCGA-DD-AAE6-01A-11R-A41C-07	300.530	57.418	0.303	High	0.94	0.9831507	0.34
TCGA-WX-AA44-01A-11R-A39D-07	250.887	23.077	0.284	High	0.9	0.9068724	0.42
TCGA-YA-A8S7-01A-11R-A37K-07	234.805	28.846	0.216	High	0.9	0.6746976	0.21
TCGA-DD-AADG-01A-11R-A41C-07	290.507	24.451	0.238	High	0.8	0.9505917	0.48
TCGA-DD-A4NL-01A-11R-A28V-07	218.028	0.000	0.028	Low	1	0.8529637	0.24
TCGA-G3-A6UC-01A-21R-A33J-07	285.104	20.879	0.289	High	1	0.9477547	0.66
TCGA-ED-A66Y-01A-11R-A311-07	283.584	17.857	0.166	Low	0.9	0.9706146	0.57
TCGA-DD-A11B-01A-11R-A131-07	287.640	23.626	0.342	High	0.9	0.936335	0.21
TCGA-BC-A10X-01A-11R-A131-07	259.259	0.000	0.047	Low	0.5	0.7713966	0.3
TCGA-DD-AAVW-01A-11R-A41C-07	223.770	30.769	0.342	High	0.8	0.7831669	0.39
TCGA-ED-A7XP-01A-11R-A352-07	274.684	28.571	0.371	High	0.88	0.8622892	0.69
TCGA-2Y-A9H9-01A-21R-A39D-07	243.947	25.549	0.248	High	0.95	0.8901443	0.27
TCGA-2Y-A9H2-01A-12R-A38B-07	255.452	68.956	0.309	High	0.7	0.8984382	0.52
TCGA-DD-A1EI-01A-11R-A131-07	238.839	25.549	0.356	High	0.7	0.8346695	0.17
TCGA-CC-A7IJ-01A-11R-A33R-07	252.727	25.824	0.176	Low	1	0.6049408	0.23
TCGA-DD-AADN-01A-11R-A41C-07	261.727	41.484	0.194	Low	0.9	0.7965695	0.3
TCGA-CC-A3MB-01A-11R-A213-07	249.434	48.077	0.309	High	0.85	0.9244493	0.31
TCGA-ED-A7PY-01A-11R-A33R-07	284.925	39.286	0.307	High	1	0.9585381	0.41
TCGA-DD-AA3A-01A-11R-A37K-07	284.291	34.066	0.273	High	0.95	0.9555192	0.75
TCGA-DD-AAD3-01A-11R-A41C-07	244.863	23.626	0.342	High	0.9	0.8118655	0.26
TCGA-FV-A2QQ-01A-11R-A22L-07	244.218	11.538	0.251	High	0.95	0.8476645	0.21
TCGA-5R-AAAM-01A-12R-A41C-07	203.303	7.967	0.199	Low	0.5	0.7345693	0.24
TCGA-ED-A66X-01A-11R-A311-07	266.578	27.198	0.305	High	0.35	0.765152	0.2
TCGA-G3-A25U-01A-11R-A16W-07	267.606	22.527	0.306	High	1	0.9430074	0.41
TCGA-DD-AACJ-01A-11R-A41C-07	277.233	26.374	0.288	High	0.8	0.9441975	0.19
TCGA-UB-AA0V-01A-11R-	220.563	0.000	0.008	Low	1	0.81200	0.57

A38B-07						6	
TCGA-G3-A5SJ-01A-11R-A27V-07	255.690	23.901	0.311	High	0.9	0.8370602	0.74
TCGA-UB-A7MB-01A-11R-A33R-07	263.841	19.231	0.282	High	0.9	0.9580805	0.23
TCGA-DD-A3A5-01A-11R-A22L-07	281.575	22.253	0.234	High	0.9	0.903391	0.82
TCGA-UB-A7MA-01A-11R-A33R-07	280.343	53.022	0.342	High	0.9	0.9214138	0.27
TCGA-WQ-A9G7-01A-11R-A37K-07	279.912	43.681	0.243	High	1	0.9546152	0.24
TCGA-WQ-AB4B-01A-11R-A41C-07	273.634	31.593	0.231	High	1	0.8503743	0.27
TCGA-G3-A7M5-01A-11R-A33R-07	261.025	18.681	0.263	High	0.85	0.9135946	0.29
TCGA-BC-A10T-01A-11R-A131-07	250.639	28.297	0.301	High	0.9	0.8455599	0.61
TCGA-O8-A75V-01A-11R-A32O-07	249.770	31.868	0.344	High	1	0.8007047	0.3
TCGA-DD-AADY-01A-11R-A41C-07	308.982	95.055	0.333	High	1	0.929291	0.38
TCGA-K7-A5RG-01A-11R-A28V-07	248.873	0.000	0.283	High	0.85	0.6415521	0.22
TCGA-2Y-A9H3-01A-11R-A38B-07	274.932	20.330	0.193	Low	0.75	0.7345554	0.24
TCGA-DD-AAEI-01A-11R-A41C-07	239.036	18.681	0.218	High	1	0.8629174	0.77
TCGA-3K-AAZ8-01A-12R-A39D-07	303.124	11.813	0.287	High	0.96	0.9069565	0.21
TCGA-DD-AAVZ-01A-11R-A41C-07	279.609	24.725	0.374	High	0.85	0.9117619	0.68
TCGA-CC-A7IK-01A-12R-A33R-07	302.725	59.615	0.303	High	1	0.9673148	0.34
TCGA-K7-A5RF-01A-11R-A28V-07	229.443	0.000	0.086	Low	0.85	0.7334438	0.59
TCGA-BC-A10W-01A-11R-A131-07	261.296	29.945	0.358	High	0.9	0.8736764	0.7
TCGA-DD-AADS-01A-11R-A41C-07	267.493	10.989	0.143	Low	0.85	0.8217606	0.25
TCGA-G3-A3CI-01A-11R-A213-07	208.805	0.549	0.059	Low	1	0.8300952	1
TCGA-G3-A7M6-01A-11R-A33R-07	281.204	27.198	0.301	High	0.8	0.9027321	0.33
TCGA-G3-A3CG-01A-11R-A213-07	240.411	18.407	0.217	High	0.98	0.8690638	0.3
TCGA-DD-A73A-01A-12R-A32O-07	229.537	8.791	0.230	High	0.9	0.8801171	0.29
TCGA-G3-A25V-01A-11R-A16W-07	223.624	0.275	0.086	Low	1	0.7797653	1
TCGA-DD-A4ND-01A-11R-A266-07	263.787	42.033	0.320	High	1	0.7186995	0.37
TCGA-2Y-A9GZ-01A-11R-A39D-07	276.307	48.352	0.285	High	0.8	0.9456446	0.22
TCGA-DD-A73G-01A-22R-A32O-07	305.272	19.231	0.193	Low	0.65	0.8786439	0.21
TCGA-CC-A7IE-01A-21R-A38B-07	267.851	26.374	0.249	High	1	0.8352591	0.19
TCGA-DD-AAVS-01A-11R-A41C-07	264.074	34.615	0.379	High	0.8	0.9062485	0.79
TCGA-DD-A73C-01A-12R-	265.055	7.143	0.111	Low	0.97	0.86399	0.65

A33J-07						33	
TCGA-DD-A3A2-01A-11R-A213-07	257.524	7.143	0.100	Low	0.95	0.8988632	0.34
TCGA-DD-AADK-01A-11R-A41C-07	267.315	22.802	0.363	High	0.5	0.8338731	0.41
TCGA-DD-A1EE-01A-11R-A131-07	268.539	15.934	0.262	High	1	0.9411266	0.3
TCGA-ZS-A9CF-01A-11R-A38B-07	263.806	26.099	0.306	High	0.65	0.8991396	0.28
TCGA-G3-AAV1-01A-11R-A38B-07	258.116	46.703	0.307	High	0.85	0.9161494	0.28
TCGA-XR-A8TE-01A-11R-A36F-07	270.509	21.429	0.143	Low	0.8	0.9005451	0.28
TCGA-DD-AAE1-01A-11R-A41C-07	291.615	27.747	0.326	High	0.97	0.9197139	0.78
TCGA-DD-A115-01A-11R-A131-07	257.596	26.099	0.301	High	0.9	0.8603667	0.27
TCGA-DD-A1EB-01A-11R-A131-07	247.446	20.055	0.179	Low	1	0.8932979	0.7
TCGA-ED-A627-01A-12R-A311-07	249.484	0.275	0.000	Low	0.8	0.6300389	0.2
TCGA-DD-AACF-01A-11R-A41C-07	258.093	27.198	0.290	High	0.6	0.8933625	0.31
TCGA-DD-AACX-01A-11R-A41C-07	254.264	17.033	0.218	High	1	0.9360698	0.47
TCGA-DD-AACC-01A-11R-A41C-07	255.230	21.703	0.235	High	0.78	0.5869494	0.32
TCGA-DD-AACZ-01A-11R-A41C-07	238.200	53.297	0.267	High	1	0.7317122	0.24
TCGA-CC-A9FW-01A-11R-A37K-07	258.823	73.626	0.336	High	0.89	0.9451365	0.29
TCGA-2Y-A9GV-01A-11R-A38B-07	244.556	10.165	0.234	High	1	0.8107413	0.29
TCGA-5C-A9VG-01A-11R-A37K-07	275.033	20.330	0.196	Low	0.5	0.9170889	0.24
TCGA-DD-AAVR-01A-11R-A41C-07	230.023	19.231	0.226	High	0.8	0.6880751	0.52
TCGA-BC-A8YO-01A-11R-A37K-07	244.285	16.484	0.282	High	0.8	0.8447941	0.43
TCGA-DD-AAEA-01A-11R-A41C-07	271.162	28.846	0.268	High	0.95	0.8970726	0.58
TCGA-QA-A7B7-01A-11R-A32O-07	280.574	27.747	0.305	High	0.9	0.9452644	0.21
TCGA-DD-A4NS-01A-11R-A311-07	243.184	0.000	0.155	Low	0.85	0.7195257	0.27
TCGA-HP-A5MZ-01A-21R-A27V-07	255.449	7.692	0.269	High	0.73	0.6801515	0.21
TCGA-MR-A8JO-01A-12R-A36F-07	233.384	8.516	0.175	Low	0.65	0.6708532	0.28
TCGA-2Y-A9GX-01A-11R-A38B-07	255.646	6.593	0.183	Low	0.9	0.6515372	0.25
TCGA-DD-A73E-01A-12R-A32O-07	259.853	23.077	0.258	High	0.88	0.9665733	0.39
TCGA-DD-AADQ-01A-11R-A41C-07	258.256	12.912	0.279	High	0.9	0.9152747	0.87
TCGA-DD-A1EJ-01A-11R-A155-07	269.060	44.505	0.290	High	0.85	0.9189324	0.24
TCGA-KR-A7K7-01A-11R-A33J-07	267.241	57.692	0.245	High	1	0.8718938	0.2
TCGA-DD-AACY-01A-11R-	275.482	19.780	0.308	High	1	0.89810	0.26

A41C-07						99	
TCGA-UB-A7ME-01A-11R-A33J-07	283.236	36.264	0.336	High	0.85	0.8196379	0.19
TCGA-G3-A25Y-01A-11R-A16W-07	266.442	29.670	0.312	High	1	0.8412222	0.24
TCGA-DD-A4NV-01A-11R-A311-07	244.035	12.363	0.298	High	0.9	0.8612124	0.29
TCGA-DD-A1EG-01A-11R-A213-07	236.043	32.418	0.301	High	0.85	0.76099	0.38
TCGA-ED-A7PX-01A-51R-A352-07	254.024	34.341	0.231	High	1	0.8432449	0.25
TCGA-CC-A7IF-01A-11R-A33J-07	276.199	34.066	0.306	High	1	0.9488624	0.67
TCGA-G3-AAV0-01A-11R-A37K-07	275.941	17.308	0.274	High	0.85	0.9064122	0.38
TCGA-G3-A5SM-01A-12R-A28V-07	231.490	26.099	0.270	High	0.7	0.7861815	0.22
TCGA-DD-AAED-01A-12R-A41C-07	283.887	40.385	0.308	High	1	0.9511561	0.26
TCGA-5R-AA1D-01A-11R-A38B-07	217.686	5.769	0.081	Low	0.8	0.6642629	0.61
TCGA-DD-AACV-01A-11R-A41C-07	311.248	34.341	0.305	High	1	0.9724159	0.28
TCGA-CC-A5UD-01A-11R-A28V-07	254.674	89.560	0.216	High	0.89	0.9482769	0.83
TCGA-CC-A9FS-01A-11R-A37K-07	290.146	25.549	0.369	High	0.93	0.9246406	0.23
TCGA-DD-AAVQ-01A-11R-A41C-07	276.037	17.033	0.290	High	0.75	0.7212249	0.3
TCGA-RC-A7SB-01A-21R-A352-07	257.526	20.604	0.308	High	1	0.9317978	0.21
TCGA-BW-A5NP-01A-11R-A27V-07	310.660	54.396	0.360	High	1	0.9695631	0.24
TCGA-DD-AADJ-01A-11R-A41C-07	250.854	18.132	0.245	High	0.9	0.9142232	0.7
TCGA-DD-AACN-01A-11R-A41C-07	315.793	35.165	0.372	High	0.7	0.8644622	0.22
TCGA-DD-AAD1-01A-11R-A41C-07	232.528	12.637	0.237	High	0.9	0.7417486	0.46
TCGA-BC-A69H-01A-11R-A311-07	250.696	56.044	0.257	High	0.85	0.8699143	0.21
TCGA-BC-A10R-01A-11R-A131-07	267.189	67.033	0.323	High	0.95	0.8702934	0.22
TCGA-ZS-A9CE-01A-11R-A37K-07	286.428	29.396	0.237	High	0.8	0.9569814	0.32
TCGA-DD-AAD0-01A-11R-A41C-07	241.656	17.033	0.232	High	0.75	0.9580717	0.84
TCGA-ZP-A9D4-01A-11R-A37K-07	259.397	17.033	0.225	High	1	0.9394664	0.86
TCGA-4R-AA8I-01A-11R-A38B-07	255.103	9.066	0.296	High	0.9	0.9060051	0.22
TCGA-FV-A3R3-01A-11R-A22L-07	217.858	6.868	0.125	Low	1	0.7593525	0.45
TCGA-LG-A6GG-01A-11R-A311-07	280.364	48.626	0.313	High	0.92	0.936139	0.29
TCGA-DD-AADO-01A-11R-A41C-07	292.296	43.407	0.269	High	0.85	0.8266276	0.2
TCGA-2Y-A9H1-01A-11R-A38B-07	273.908	18.132	0.218	High	0.85	0.8566748	0.79
TCGA-BC-A10Y-01A-11R-	260.666	26.923	0.247	High	0.85	0.89578	0.26

A131-07						19	
TCGA-G3-A5SI-01A-31R-A27V-07	281.584	28.846	0.293	High	0.85	0.9530955	0.89
TCGA-CC-A5UE-01A-11R-A28V-07	280.305	45.604	0.321	High	0.93	0.9599582	0.27
TCGA-DD-AADU-01A-11R-A41C-07	267.753	83.791	0.244	High	0.4	0.946841	0.29
TCGA-DD-AAW3-01A-11R-A41C-07	298.783	21.154	0.327	High	0.8	0.9377396	0.72
TCGA-G3-AAV5-01A-11R-A37K-07	269.221	31.593	0.233	High	1	0.9356219	0.3
TCGA-CC-A7IL-01A-11R-A33R-07	304.318	50.000	0.320	High	1	0.9714695	0.2
TCGA-DD-AAE4-01A-11R-A41C-07	262.484	15.934	0.167	Low	1	0.7071768	0.21
TCGA-2Y-A9GT-01A-11R-A38B-07	220.526	1.923	0.172	Low	1	0.7505614	0.49
TCGA-DD-AACH-01A-11R-A41C-07	243.067	34.341	0.326	High	0.8	0.8958302	0.79
TCGA-EP-A2KA-01A-11R-A180-07	236.229	23.901	0.222	High	0.95	0.8272054	0.27
TCGA-DD-AADI-01A-11R-A41C-07	271.844	26.099	0.273	High	0.9	0.7628809	0.57
TCGA-RC-A6M4-01A-11R-A32O-07	265.721	39.560	0.273	High	0.8	0.9658891	0.25
TCGA-RC-A6M3-01A-11R-A32O-07	270.870	48.626	0.203	High	0.9	0.9417551	0.21
TCGA-DD-A3A9-01A-11R-A266-07	283.756	65.385	0.266	High	0.7	0.8103622	0.19
TCGA-G3-A25T-01A-11R-A16W-07	285.980	14.286	0.195	Low	1	0.8851941	0.25
TCGA-ES-A2HT-01A-12R-A180-07	261.613	11.538	0.186	Low	0.9	0.832763	0.59
TCGA-ES-A2HS-01A-11R-A180-07	311.565	32.143	0.251	High	0.9	0.9053098	0.28
TCGA-KR-A7K0-01A-12R-A33R-07	251.401	5.220	0.190	Low	1	0.8834465	0.23
TCGA-K7-AAU7-01A-11R-A38B-07	222.761	14.560	0.252	High	1	0.8524845	0.53
TCGA-DD-A73F-01A-11R-A32O-07	240.798	10.165	0.208	High	0.73	0.7263722	0.25
TCGA-G3-AAV6-01A-21R-A37K-07	287.045	70.330	0.293	High	1	0.9580632	0.26
TCGA-DD-AACL-01A-11R-A41C-07	252.838	29.396	0.244	High	0.65	0.7682261	0.27
TCGA-XR-A8TD-01A-12R-A39D-07	249.095	27.747	0.245	High	0.75	0.6555336	0.44
TCGA-DD-A11C-01A-11R-A131-07	244.326	51.923	0.264	High	0.8	0.8347737	0.44
TCGA-MR-A520-01A-11R-A266-07	250.506	0.000	-0.016	Low	0.7	0.9073543	0.7
TCGA-DD-AAVU-01A-11R-A41C-07	282.965	87.637	0.298	High	0.8	0.9410124	0.67
TCGA-2Y-A9GY-01A-11R-A38B-07	262.723	42.308	0.279	High	1	0.8161634	0.21
TCGA-DD-A39V-01A-11R-A213-07	241.350	25.549	0.233	High	0.8	0.7975145	0.25
TCGA-BC-A112-01A-11R-A131-07	221.344	28.571	0.272	High	0.8	0.8601886	0.18
TCGA-ZP-A9CY-01A-11R-	249.760	3.022	0.142	Low	1	0.82915	0.22

A38B-07						68	
TCGA-CC-A3M9-01A-11R-A213-07	284.039	28.022	0.115	Low	0.7	0.44901	0.25
TCGA-2Y-A9HB-01A-11R-A39D-07	254.293	17.308	0.232	High	0.9	0.8174379	0.61
TCGA-DD-AADR-01A-11R-A41C-07	241.027	31.319	0.287	High	0.9	0.8957376	0.24
TCGA-G3-AAUZ-01A-11R-A38B-07	271.209	23.626	0.221	High	0.85	0.8274767	0.25
TCGA-MI-A75C-01A-11R-A32O-07	282.129	16.484	0.220	High	0.93	0.9286836	0.64

Table S1c. Table for PCC of LCI cohort

Sample ID	PCC	Group
LCS_004A	-0.0168	Low
LCS_007A	0.0477	Low
LCS_023A	0.1302	High
LCS_067A	0.1584	High
LCS_065A	0.1331	High
LCS_073A	0.0779	Low
LCS_024A	0.1620	High
LCS_042A	0.2200	High
LCS_062A	0.1259	High
LCS_015A	0.1077	High
LCS_069A	0.1808	High
LCS_033A	0.0652	Low
LCS_061A	0.1397	High
LCS_020A	0.0613	Low
LCS_045A	0.1535	High
LCS_057A	0.1688	High
LCS_009A	0.2959	High
LCS_049A	0.0640	Low
LCS_047A	0.2006	High
LCS_075A	0.0594	Low
LCS_034A	0.0456	Low
LCS_041A	0.2258	High
LCS_044A	-0.0030	Low
LCS_071A	0.1441	High
LCS_036A	0.1864	High
LCS_064A	0.2237	High
LCS_038A	0.1564	High
LCS_002A	0.0885	Low
LCS_054A	0.1344	High
LCS_063A	0.1109	High
LCS_022A	0.1863	High
LCS_025A	0.0539	Low
LCS_066A	0.1591	High
LCS_027A	0.1703	High
LCS_048A	0.0992	Low
LCS_012A	0.1333	High
LCS_051A	0.1051	High

LCS_056A	0.1909	High
LCS_028A	0.1985	High
LCS_016A	0.1540	High
LCS_196A	0.1571	High
LCS_072A	0.1593	High
LCS_357A	0.1484	High
LCS_005A	0.1554	High
LCS_019A	0.1922	High
LCS_029A	0.1017	High
LCS_039A	0.0971	Low
LCS_254A	0.0431	Low
LCS_068A	0.0990	Low
LCS_076A	0.3269	High
LCS_333A	0.2193	High
LCS_339A	0.0606	Low
LCS_341A	0.0750	Low
LCS_343A	0.1621	High
LCS_344A	0.1981	High
LCS_346A	0.1155	High
LCS_347A	0.2424	High
LCS_393A	0.1158	High
LCS_400A	0.2249	High
LCS_401A	0.1066	High
LCS_403A	0.1890	High
LCS_406A	0.1228	High
LCS_415A	0.1272	High
LCS_424A	0.2341	High
LCS_426A	0.1391	High

Table S2. Overlapped Differentially Expressed Genes among correlated genes between Thai HCC and Thai iCCA

	Probe ID	Official Symbol	HCC						iCCA					
			LFGC (Mean)	HFGC (Mean)	perm.p	Fold Change	cor.r	cor.p	LFGC (Mean)	HFGC (Mean)	perm.p	Fold Change	cor.r	cor.p
HFGC_UP	TC17001769.hg	BRIP1	0.0142	1.1001	0.0017	1.0860	0.5851	0.0000	0.2369	0.9013	0.0024	0.6645	0.4350	0.0000
	TC05000134.hg	C5orf22	0.0416	0.6175	0.0018	0.5759	0.5762	0.0000	-0.0638	0.6110	0.0000	0.6748	0.5907	0.0000
	TC03001882.hg	HLTF	0.1843	1.0937	0.0028	0.9094	0.5510	0.0000	-0.2542	0.5102	0.0015	0.7644	0.4663	0.0000
	TC08000701.hg	MTBP	0.0354	0.8016	0.0004	0.7662	0.5097	0.0000	0.1454	0.8641	0.0001	0.7187	0.6017	0.0000
	TC01001781.hg	FLVCR1	0.4805	1.4848	0.0008	1.0043	0.5002	0.0000	0.3281	1.0638	0.0002	0.7357	0.4285	0.0000
	TC10001914.hg	-	0.1514	0.7716	0.0039	0.6202	0.4810	0.0001	-0.0738	0.5058	0.0001	0.5796	0.4807	0.0000
	TC19001630.hg	-	-0.2373	0.6659	0.0039	0.9032	0.4580	0.0002	-0.1117	0.6015	0.0003	0.7133	0.3103	0.0029
	TC01005322.hg	-	-0.2565	0.3482	0.0031	0.6046	0.4102	0.0009	-0.1403	0.5285	0.0014	0.6688	0.6045	0.0000
	TC20000063.hg	MCM8	0.0263	0.9355	0.0043	0.9093	0.3707	0.0030	0.4325	1.5114	0.0000	1.0789	0.5561	0.0000
	TC07001654.hg	MCM7	-0.0801	0.6606	0.0006	0.7408	0.3695	0.0031	0.1244	0.9751	0.0000	0.8507	0.5547	0.0000
	TC05000161.hg	SKP2	-0.0199	1.0414	0.0009	1.0614	0.3537	0.0048	-0.8532	0.1413	0.0000	0.9945	0.5339	0.0000
	TC09002185.hg	-	-0.1209	0.7151	0.0039	0.8360	0.3309	0.0086	-0.5622	-0.0146	0.0003	0.5477	0.3671	0.0004
	TC01002916.hg	SASS6	-0.0896	0.6298	0.0030	0.7194	0.3192	0.0114	0.3593	1.0462	0.0001	0.6868	0.3587	0.0005
	TC20000784.hg	PIGU	0.2316	1.0044	0.0015	0.7728	0.2949	0.0200	0.1652	0.8425	0.0000	0.6773	0.6154	0.0000
	TC08000695.hg	MAL2	-0.9544	0.2122	0.0025	1.1665	0.2941	0.0203	0.6231	1.6283	0.0001	1.0052	0.5509	0.0000
	TC17002618.hg	-	0.1968	1.9895	0.0049	1.7927	0.2895	0.0225	1.4700	3.0272	0.0000	1.5572	0.3490	0.0007
	TC12001605.hg	TIMELESS	0.0507	0.8189	0.0017	0.7682	0.2763	0.0297	-0.3542	0.3319	0.0002	0.6860	0.5740	0.0000
HFGC_DN	TC16000443.hg	CYLD	0.1088	-0.4176	0.0008	-0.5264	0.6379	0.0000	0.1743	-0.5150	0.0000	-0.6893	0.3223	0.0019
	TC04001323.hg	CNOT6L	0.3452	-0.3297	0.0003	-0.6749	0.6173	0.0000	0.1911	-0.3290	0.0002	-0.5201	0.4735	0.0000
	TC05001570.hg	CCNH	0.0704	-0.5432	0.0003	-0.6136	0.4298	0.0005	0.0399	-0.5382	0.0000	-0.5782	0.6222	0.0000
	TC09000035.hg	JAK2	0.1345	-0.5793	0.0010	-0.7138	0.4244	0.0006	0.9534	0.2215	0.0012	-0.7319	0.3768	0.0003
	TC05001367.hg	LOC100652914	0.2876	-0.5078	0.0045	-0.7954	0.3987	0.0013	-0.8985	-1.5924	0.0001	-0.6939	0.2842	0.0066
	TC09000037.hg	CD274	0.3362	-0.3846	0.0012	-0.7208	0.3534	0.0048	0.3626	-0.3030	0.0011	-0.6655	0.3748	0.0003
	TC09001325.hg	NFIL3	-0.0454	-0.8257	0.0034	-0.7803	0.3426	0.0064	-0.6668	-1.3746	0.0003	-0.7078	0.2388	0.0234
	TC11002246.hg	CASP4	0.1596	-0.5252	0.0007	-0.6848	0.3013	0.0173	0.3340	-0.1859	0.0008	-0.5199	0.4825	0.0000
	TC17002849.hg	PER1	0.0407	-0.6408	0.0010	-0.6814	0.2963	0.0194	-0.3776	-0.9433	0.0001	-0.5657	0.2691	0.0103
	TC17001053.hg	SCIMP	0.2858	-0.6687	0.0002	-0.9545	0.2812	0.0269	-0.0821	-0.6832	0.0009	-0.6011	0.3166	0.0024
	TC08000310.hg	C8orf4	-0.4859	-1.8605	0.0033	-1.3745	0.2598	0.0414	-0.0326	-1.2503	0.0001	-1.2176	0.2699	0.0101
	TC07001295.hg	TRGV10	0.3831	-0.3456	0.0012	-0.7288	-0.2387	0.0617	0.2736	-0.5858	0.0000	-0.8594	-0.2417	0.0217

Table S3. Comparison table of overlapped differentially expressed genes among correlated genes.

Comparison	Overlapped gene	
	HFGC_UP	HFGC_DN
**Thai HCC vs. Thai iCCA**	FLVCR1, SASS6, HLTF, C5orf22, SKP2, MCM7, MAL2, MTBP, TIMELESS, BRIP1, PIGU	CNOT6L, C8orf4, JAK2, NFIL3, CASP4, CYLD, SCIMP
**Thai HCC vs. TCGA_HCC**	ANXA9, ASPM, BRIP1, BRPF3, CAP2, CENPQ, FDPS, FLVCR1, INTS7, MAL2, MCM3, MCM7, MOGAT3, MTBP, PRIM2, SASS6, SQLE, STXBP6, TIMELESS, VPS72, ZNF572	ALPK1, AOAH, SCIMP, CHD9, COTL1, EPSTI1, FYB, HLA-DOA, IRF8, NDEL1, NFIL3, PPM1K, RGS2, SLAMF6, ZC3H13
**Thai iCCA vs. TCGA_HCC**	ACTL6A, ANKRD27, ATAD2, ATAD5, AURKA, BRCA1, BRIP1, PARPBP, BORA, CASC5, CEP85, CCT3, CDC20, CDC45, CDC6, CDCA8, CENPA, CENPO, CKS2, DNA2, DSCC1, DSN1, E2F7, ECT2, EFNA4, EZH2, FAM83D, FANCD2, FANCI, FLVCR1, FOXM1, GDPD1, GINS1, GPSM2, IGF2BP3, INTS8, IQGAP3, KIF15, KIF20B, KIF2C, KIFC1, KNTC1, KPNA2, LAPTM4B, LLGL2, LSR, MAL2, MCM4, MCM7, MELK, MSH2, MTBP, MYBL2, NCAPG2, NDC80, NEK2, NUP155, PBK, PCNA, POLQ, PRIM1, PSPH, RACGAP1, RAD51AP1, RBL1, RFC3, RFC4, RRM2, SASS6, SHCBP1, SKA1, TIMELESS, TKT, TOP2A, TRIP13, TUBA1B, TUBG1, TYMS, UBE2C, UBE2T, XPO5, XRCC2, ZNF623	AIF1, SCIMP, CDC37L1, CLEC10A, CSGALNACT1, CTSO, DOCK8, ETS1, GAS7, IL7R, JUNB, NAT2, NFIL3, PDCD1LG2, PECAM1, PMP22, RCAN1
**Thai HCC vs. Thai iCCA vs. TCGA HCC**	BRIP1, FLVCR1, MAL2, MCM7, MTBP, SASS6, TIMELESS	SCIMP, NFIL3

Table S4. GSEA result compared HFGC with LFGC of Thai iCCA

Biological Process (HFGC)								
NAME	SIZE	ES	NES	NOM p-val	FDR q-val	FWER p-val	RANK AT MAX	LEADING EDGE
DNA_DEPENDENT_DNA_REPLICATION	43	-0.674	-1.907	0.002	0.2221	0.122	2601	tags=60%, list=19%, signal=75%
MITOSIS	50	-0.689	-1.882	0.002	0.1576	0.166	1564	tags=52%, list=12%, signal=59%
M_PHASE_OF_MITOTIC_CELL_CYCLE	53	-0.685	-1.861	0.002	0.1475	0.204	1564	tags=51%, list=12%, signal=57%
M_PHASE	70	-0.664	-1.855	0.004	0.1176	0.21	1855	tags=53%, list=14%, signal=61%
CELL_CYCLE_PHASE	107	-0.593	-1.837	0.0079	0.1166	0.242	1564	tags=42%, list=12%, signal=47%
CELL_CYCLE_PROCESS	124	-0.601	-1.834	0.0041	0.1008	0.249	1957	tags=47%, list=15%, signal=54%
MITOTIC_CELL_CYCLE	99	-0.604	-1.82	0.0081	0.1015	0.277	1766	tags=43%, list=13%, signal=50%
DNA_REPLICATION	75	-0.599	-1.804	0.008	0.1073	0.307	2620	tags=52%, list=19%, signal=64%
CHROMOSOME_SEGREGATION	21	-0.702	-1.801	0.0039	0.0981	0.309	1024	tags=48%, list=8%, signal=51%
CELL_CYCLE_GO_0007049	215	-0.505	-1.799	0.0101	0.0907	0.313	2042	tags=38%, list=15%, signal=44%
DNA_INTEGRITY_CHECKPOINT	16	-0.719	-1.785	0.012	0.0955	0.34	2579	tags=63%, list=19%, signal=77%
REGULATION_OF_MITOSIS	26	-0.682	-1.784	0.008	0.0885	0.342	1564	tags=54%, list=12%, signal=61%
MICROTUBULE_CYTOSKELETON_ORGANIZATION_AND_BIOGENESIS	28	-0.632	-1.747	0.0251	0.1188	0.431	3152	tags=61%, list=23%, signal=79%
MICROTUBULE_BASED_PROCESS	60	-0.518	-1.744	0.008	0.1132	0.437	3152	tags=47%, list=23%, signal=61%
CHROMATIN_MODIFICATION	39	-0.571	-1.731	0.0078	0.1195	0.469	3230	tags=56%, list=24%, signal=74%
CHROMOSOME_ORGANIZATION_AND_BIOGENESIS	82	-0.54	-1.73	0.0138	0.1138	0.471	3230	tags=54%, list=24%, signal=70%
CELL_CYCLE_CHECKPOINT_GO_0000075	34	-0.614	-1.705	0.0356	0.1347	0.524	3112	tags=59%, list=23%, signal=76%
MEIOTIC_CELL_CYCLE	21	-0.619	-1.702	0.0149	0.1315	0.535	1855	tags=52%, list=14%, signal=61%

INTERPHASE_OF_MITOTIC_CELL_CYCLE	37	-0.564	-1.686	0.0163	0.1442	0.573	4206	tags=62%, list=31%, signal=90%
PHOSPHOINOSITIDE_BIOSYNTHETIC_PROCESS	20	-0.642	-1.685	0.0187	0.1384	0.579	2275	tags=50%, list=17%, signal=60%
**Biological Process (LFGC)**								
NAME	SIZE	ES	NES	NOM p-val	FDR q-val	FWER p-val	RANK AT MAX	LEADING EDGE
DEFENSE_RESPONSE	189	0.6299	1.8836	0.0162	0.4864	0.174	2508	tags=59%, list=19%, signal=71%
RESPONSE_TO_WOUNDING	142	0.6262	1.8659	0.0081	0.2967	0.204	2839	tags=61%, list=21%, signal=77%
INFLAMMATORY_RESPONSE	100	0.6446	1.8588	0.0142	0.2127	0.211	2508	tags=61%, list=19%, signal=74%
IMMUNE_RESPONSE	152	0.6352	1.8528	0.0233	0.1687	0.221	2832	tags=64%, list=21%, signal=80%
IMMUNE_SYSTEM_PROCESS	219	0.5803	1.8454	0.0307	0.147	0.231	2848	tags=58%, list=21%, signal=72%
POSITIVE_REGULATION_OF_MULTICELLULAR_ORGANISMAL_PROCESS	42	0.6503	1.8285	0.0101	0.1507	0.263	2949	tags=60%, list=22%, signal=76%
RESPONSE_TO_OTHER_ORGANISM	48	0.6462	1.8282	0.0161	0.1298	0.263	2547	tags=63%, list=19%, signal=77%
CYTOKINE_PRODUCTION	41	0.6209	1.7841	0.0219	0.1792	0.362	2949	tags=61%, list=22%, signal=78%
POSITIVE_REGULATION_OF_TRANSLATION	21	0.6338	1.7774	0.014	0.1697	0.376	2190	tags=62%, list=16%, signal=74%
LOCOMOTORY_BEHAVIOR	74	0.6033	1.7728	0.0164	0.1596	0.386	2820	tags=64%, list=21%, signal=80%
RESPONSE_TO_EXTERNAL_STIMULUS	213	0.5415	1.7694	0.0164	0.1505	0.394	2848	tags=55%, list=21%, signal=69%
MULTI_ORGANISM_PROCESS	97	0.5187	1.7675	0.0182	0.1408	0.394	3121	tags=55%, list=23%, signal=71%
POSITIVE_REGULATION_OF_IMMUNE_SYSTEM_PROCESS	32	0.652	1.7663	0.0217	0.1321	0.397	1006	tags=41%, list=7%, signal=44%
POSITIVE_REGULATION_OF_CYTOKINE_BIOSYNTHETIC_PROCESS	15	0.7064	1.7575	0.0164	0.1328	0.423	2190	tags=67%, list=16%, signal=80%
CELLULAR_DEFENSE_RESPONSE	40	0.6893	1.7565	0.0291	0.1252	0.427	1874	tags=63%, list=14%, signal=72%
RESPONSE_TO_BACTERIUM	15	0.7077	1.7227	0.0163	0.1574	0.505	2488	tags=67%, list=18%, signal=82%

REGULATION_OF_IMMUNE_SYSTEM_PROCESS	39	0.6153	1.7226	0.0339	0.1484	0.505	2848	tags=56%, list=21%, signal=71%
POSITIVE_REGULATION_OF_IMMUNE_RESPONSE	18	0.7265	1.7208	0.0142	0.1422	0.511	1987	tags=61%, list=15%, signal=72%
REGULATION_OF_CYTOKINE_BIOSYNTHETIC_PROCESS	21	0.6296	1.7202	0.0198	0.1351	0.513	2190	tags=57%, list=16%, signal=68%
RESPONSE_TO_VIRUS	31	0.6363	1.7147	0.0257	0.134	0.525	3695	tags=81%, list=27%, signal=111%
**KEGG Pathway (HFGC)**								
NAME	SIZE	ES	NES	NOM p-val	FDR q-val	FWER p-val	RANK AT MAX	LEADING EDGE
KEGG_DNA_REPLICATION	29	-0.812	-1.86	0.0019	0.1886	0.125	969	tags=72%, list=7%, signal=78%
KEGG_MISMATCH_REPAIR	19	-0.742	-1.776	0.0059	0.2655	0.267	1688	tags=63%, list=13%, signal=72%
KEGG_BASE_EXCISION_REPAIR	21	-0.676	-1.739	0.0077	0.2569	0.338	3359	tags=71%, list=25%, signal=95%
KEGG_CELL_CYCLE	90	-0.61	-1.721	0.0211	0.2324	0.388	2306	tags=52%, list=17%, signal=63%
KEGG_HOMOLOGOUS_RECOMBINATION	16	-0.71	-1.689	0.0134	0.2545	0.464	1855	tags=63%, list=14%, signal=72%
KEGG_RNA_POLYMERASE	23	-0.654	-1.645	0.0059	0.3047	0.555	2754	tags=65%, list=20%, signal=82%
KEGG_OOCYTE_MEIOSIS	77	-0.488	-1.616	0.0206	0.3265	0.618	2892	tags=44%, list=21%, signal=56%
KEGG_AMINOACYL_TRNA_BIOSYNTHESIS	30	-0.625	-1.601	0.0329	0.3195	0.648	3080	tags=67%, list=23%, signal=86%
KEGG_PYRIMIDINE_METABOLISM	62	-0.506	-1.591	0.0476	0.308	0.675	3502	tags=56%, list=26%, signal=76%
KEGG_NUCLEOTIDE_EXCISION_REPAIR	34	-0.571	-1.588	0.0355	0.2814	0.678	3395	tags=50%, list=25%, signal=67%
KEGG_N_GLYCAN_BIOSYNTHESIS	37	-0.502	-1.562	0.0462	0.3011	0.729	3637	tags=62%, list=27%, signal=85%
KEGG_SPLICEOSOME	96	-0.531	-1.552	0.0825	0.2975	0.747	4748	tags=68%, list=35%, signal=104%
KEGG_BASAL_TRANSCRIPTION_FACTORS	24	-0.564	-1.548	0.0511	0.2811	0.754	933	tags=33%, list=7%, signal=36%
KEGG_UBIQUITIN_MEDIATED_PROTEOLYSIS	105	-0.455	-1.542	0.0405	0.2726	0.765	3469	tags=46%, list=26%, signal=61%
KEGG_RNA_DEGRADATION	44	-0.524	-1.508	0.0807	0.312	0.815	3259	tags=48%, list=24%,

								signal=63%
KEGG_OXIDATIVE_PHOSPHORYLATION	89	-0.523	-1.498	0.1	0.3092	0.826	4764	tags=67%, list=35%, signal=104%
KEGG_GLYCOSYLPHOSPHATIDYLINOSITOL_GPI_ANCHOR_BIOSYNTHESIS	18	-0.582	-1.495	0.0538	0.2962	0.829	3900	tags=67%, list=29%, signal=94%
KEGG_HUNTINGTONS_DISEASE	120	-0.462	-1.469	0.0904	0.3207	0.854	4145	tags=52%, list=31%, signal=75%
KEGG_PROGESTERONE_MEDIATED_OOCYTE_MATURATION	54	-0.441	-1.447	0.0696	0.3396	0.876	1134	tags=28%, list=8%, signal=30%
**KEGG Pathway (LFGC)**								
NAME	SIZE	ES	NES	NOM p-val	FDR q-val	FWER p-val	RANK AT MAX	LEADING EDGE
KEGG_HEMATOPOIETIC_CELL_LINEAGE	49	0.7264	1.8663	0.002	0.2115	0.134	1945	tags=63%, list=14%, signal=74%
KEGG_CYTOKINE_CYTOKINE_RECEPTOR_INTERACTION	156	0.5901	1.8433	0.002	0.1416	0.167	3003	tags=60%, list=22%, signal=77%
KEGG_NATURAL_KILLER_CELL_MEDIATED_CYTOTOXICITY	80	0.5364	1.6603	0.0455	0.5888	0.516	1202	tags=40%, list=9%, signal=44%
KEGG_NEUROACTIVE_LIGAND_RECEPTOR_INTERACTION	108	0.4393	1.6404	0.0165	0.5135	0.557	2646	tags=40%, list=20%, signal=49%
KEGG_CELL_ADHESION_MOLECULES_CAMS	93	0.5438	1.6371	0.052	0.4226	0.56	2500	tags=49%, list=19%, signal=60%
KEGG_CHEMOKINE_SIGNALING_PATHWAY	142	0.458	1.5851	0.0538	0.5215	0.668	1088	tags=31%, list=8%, signal=33%
KEGG_B_CELL_RECEPTOR_SIGNALING_PATHWAY	58	0.4919	1.5708	0.0593	0.4922	0.693	1362	tags=40%, list=10%, signal=44%
KEGG_LEUKOCYTE_TRANSENDOTHELIAL_MIGRATION	76	0.4963	1.5683	0.0641	0.4378	0.695	1611	tags=38%, list=12%, signal=43%
KEGG_T_CELL_RECEPTOR_SIGNALING_PATHWAY	77	0.4747	1.567	0.0559	0.3925	0.7	1517	tags=38%, list=11%, signal=42%
KEGG_COMPLEMENT_AND_COAGULATION_CASCADES	59	0.734	1.5668	0.0539	0.3536	0.701	2577	tags=80%, list=19%, signal=98%
KEGG_JAK_STAT_SIGNALING_PATHWAY	84	0.4794	1.5541	0.0713	0.3496	0.724	2208	tags=44%, list=16%, signal=52%
KEGG_NOD_LIKE_RECEPTOR_SIGNALING_PATHWAY	48	0.5046	1.5262	0.082	0.3787	0.756	1717	tags=42%, list=13%, signal=48%
KEGG_INTESTINAL_IMMUNE_NETWORK_FOR_IG	29	0.6359	1.4996	0.1064	0.4074	0.795	4060	tags=86%, list=30%,

A_PRODUCTION								signal=123%
KEGG_GRAFT_VERSUS_HOST_DISEASE	25	0.7296	1.4963	0.0682	0.3859	0.799	2435	tags=76%, list=18%, signal=93%
KEGG_PRIMARY_IMMUNODEFICIENCY	26	0.6269	1.4957	0.1086	0.361	0.8	2588	tags=73%, list=19%, signal=90%
KEGG_AUTOIMMUNE_THYROID_DISEASE	23	0.6945	1.4832	0.0969	0.3616	0.817	2877	tags=74%, list=21%, signal=94%
KEGG_ARACHIDONIC_ACID_METABOLISM	32	0.5242	1.4747	0.1006	0.3558	0.829	3686	tags=69%, list=27%, signal=94%
KEGG_TYPE_II_DIABETES_MELLITUS	31	0.4034	1.4685	0.0393	0.3455	0.833	585	tags=23%, list=4%, signal=24%
KEGG_PRION_DISEASES	29	0.5504	1.4596	0.092	0.3433	0.842	2577	tags=59%, list=19%, signal=72%
KEGG_NICOTINATE_AND_NICOTINAMIDE_METABOLISM	17	0.4906	1.4434	0.0658	0.3543	0.856	2290	tags=35%, list=17%, signal=42%
**Oncogenic (HFGC)**								
NAME	SIZE	ES	NES	NOM p-val	FDR q-val	FWER p-val	RANK AT MAX	LEADING EDGE
RB_DN.V1_UP	89	-0.564	-1.931	0.0039	0.0198	0.038	2798	tags=52%, list=21%, signal=65%
RB_P107_DN.V1_UP	88	-0.606	-1.923	0.0059	0.0108	0.041	1688	tags=51%, list=13%, signal=58%
PRC2_EZH2_UP.V1_UP	117	-0.475	-1.778	0.0115	0.0513	0.181	1547	tags=35%, list=11%, signal=39%
RPS14_DN.V1_DN	132	-0.515	-1.764	0.0213	0.0462	0.205	4011	tags=59%, list=30%, signal=83%
E2F1_UP.V1_UP	123	-0.517	-1.757	0.0179	0.04	0.219	3182	tags=51%, list=24%, signal=66%
GCNP_SHH_UP_EARLY.V1_UP	115	-0.447	-1.653	0.0189	0.0852	0.406	2257	tags=37%, list=17%, signal=45%
CSR_LATE_UP.V1_UP	125	-0.503	-1.616	0.0247	0.1003	0.478	2489	tags=46%, list=18%, signal=56%
RB_P130_DN.V1_UP	78	-0.418	-1.616	0.0203	0.0879	0.478	2950	tags=38%, list=22%, signal=49%
PRC2_EDD_UP.V1_UP	110	-0.443	-1.564	0.0506	0.1202	0.581	2160	tags=34%, list=16%, signal=40%
HOXA9_DN.V1_DN	135	-0.406	-1.535	0.0503	0.1341	0.629	2943	tags=38%, list=22%, signal=48%
VEGF_A_UP.V1_DN	147	-0.399	-1.447	0.1016	0.2237	0.764	2360	tags=33%, list=18%, signal=39%

E2F3_UP.V1_UP	105	-0.355	-1.407	0.0911	0.258	0.81	1986	tags=28%, list=15%, signal=32%
SIRNA_EIF4GI_UP	73	-0.403	-1.405	0.1023	0.2412	0.812	3063	tags=45%, list=23%, signal=58%
GCNP_SHH_UP_LATE.V1_UP	128	-0.371	-1.398	0.1212	0.234	0.822	3118	tags=39%, list=23%, signal=50%
EGFR_UP.V1_DN	135	-0.323	-1.391	0.0588	0.2267	0.826	3102	tags=33%, list=23%, signal=43%
TBK1.DN.48HRS_UP	39	-0.403	-1.341	0.1044	0.2821	0.891	2892	tags=38%, list=21%, signal=49%
TBK1.DF_DN	228	-0.337	-1.34	0.1445	0.2661	0.891	4018	tags=41%, list=30%, signal=57%
MYC_UP.V1_UP	105	-0.403	-1.321	0.1899	0.2775	0.91	2375	tags=33%, list=18%, signal=40%
EIF4E_UP	54	-0.391	-1.288	0.1908	0.3111	0.933	3506	tags=46%, list=26%, signal=62%
RB_P107_DN.V1_DN	94	-0.339	-1.243	0.1879	0.3658	0.955	3218	tags=36%, list=24%, signal=47%
**Oncogenic (LFGC)**								
NAME	SIZE	ES	NES	NOM p-val	FDR q-val	FWER p-val	RANK AT MAX	LEADING EDGE
P53_DN.V2_UP	79	0.5704	1.9195	0	0.102	0.048	2412	tags=44%, list=18%, signal=54%
RPS14_DN.V1_UP	142	0.6025	1.8577	0.0081	0.1009	0.09	2491	tags=54%, list=18%, signal=66%
EGFR_UP.V1_UP	152	0.5307	1.8076	0.014	0.1238	0.143	2685	tags=45%, list=20%, signal=55%
P53_DN.V1_DN	133	0.5355	1.7705	0.0101	0.1346	0.192	2926	tags=52%, list=22%, signal=66%
RELA_DN.V1_UP	91	0.4621	1.7161	0.0141	0.1838	0.29	2402	tags=38%, list=18%, signal=46%
KRAS.PROSTATE_UP.V1_UP	71	0.5174	1.6938	0.0145	0.1842	0.334	3189	tags=58%, list=24%, signal=75%
HOXA9_DN.V1_UP	147	0.5021	1.6755	0.039	0.1848	0.369	1942	tags=37%, list=14%, signal=42%
KRAS.LUNG.BREAST_UP.V1_UP	88	0.4688	1.6688	0.012	0.1709	0.379	2890	tags=43%, list=21%, signal=55%
PTEN_DN.V2_UP	94	0.4766	1.6472	0.0261	0.1827	0.416	2132	tags=40%, list=16%, signal=48%
PTEN_DN.V1_DN	96	0.4537	1.6429	0.020	0.171	0.424	3254	tags=49%,

				4	4			list=24%, signal=64%
ATM_DN.V1_UP	79	0.4708	1.6424	0.020 4	0.156 3	0.425	3473	tags=51%, list=26%, signal=68%
STK33_NOMO_UP	227	0.4533	1.6374	0.045 6	0.148 7	0.435	2294	tags=39%, list=17%, signal=46%
STK33_UP	225	0.4324	1.6319	0.050 4	0.144	0.445	2417	tags=41%, list=18%, signal=49%
KRAS.BREAST_UP.V1_UP	71	0.4614	1.6316	0.032 8	0.134 3	0.447	3579	tags=48%, list=27%, signal=65%
KRAS.LUNG_UP.V1_UP	81	0.4791	1.6239	0.020 7	0.131 8	0.459	2890	tags=47%, list=21%, signal=59%
CRX_DN.V1_DN	86	0.4552	1.623	0.023 5	0.124	0.46	1958	tags=34%, list=15%, signal=39%
HINATA_NFKB_IMMU_IN F	16	0.7641	1.6149	0.029 6	0.124 9	0.475	2133	tags=81%, list=16%, signal=96%
IL2_UP.V1_DN	114	0.3958	1.6109	0.006 5	0.122	0.486	2989	tags=38%, list=22%, signal=48%
STK33_SKM_UP	214	0.4085	1.6005	0.059 3	0.125 6	0.507	2417	tags=40%, list=18%, signal=48%
KRAS.BREAST_UP.V1_DN	74	0.452	1.5994	0.016 6	0.120 3	0.508	2493	tags=38%, list=18%, signal=46%

Table S5. GSEA result compared HFGC with LFGC of Thai HCC

**Biological Process (HFGC)**								
NAME	SIZE	ES	NES	NOM p-val	FDR q-val	FWER p-val	RANK AT MAX	LEADING EDGE
DNA_DEPENDENT_DNA_REPLICATION	40	-0.62918	-1.76602	0.022133	1	0.379	2617	tags=52%, list=19%, signal=65%
GOLGI_VESICLE_TRANSPORT	33	-0.56013	-1.71775	0.008621	0.912368	0.504	2368	tags=39%, list=17%, signal=48%
CHROMOSOME_SEGREGATION	21	-0.63975	-1.63112	0.059305	1	0.709	2362	tags=62%, list=17%, signal=75%
DNA_REPLICATION	70	-0.53649	-1.61436	0.056112	1	0.744	2617	tags=46%, list=19%, signal=56%
CHROMATIN_REMODELING	18	-0.63872	-1.61236	0.034205	0.932113	0.745	2705	tags=56%, list=20%, signal=69%
REGULATION_OF_DNA_REPLICATION	15	-0.61605	-1.54532	0.056112	1	0.839	1571	tags=47%, list=12%, signal=53%
DNA_PACKAGING	23	-0.55972	-1.52803	0.079051	1	0.86	2852	tags=52%, list=21%, signal=66%
LIPOPROTEIN_BIOSYNTHETIC_PROCESS	20	-0.54358	-1.52769	0.065764	1	0.861	2499	tags=40%, list=18%, signal=49%
PHOSPHOINOSITIDE_METABOLIC_PROCESS	25	-0.49369	-1.47285	0.089796	1	0.905	2499	tags=40%, list=18%, signal=49%
SECRETORY_PATHWAY	53	-0.42492	-1.4727	0.048583	1	0.905	2368	tags=36%, list=17%, signal=43%
LIPID_BIOSYNTHETIC_PROCESS	68	-0.38891	-1.45206	0.069364	1	0.919	2499	tags=32%, list=18%, signal=39%
GLYCEROPHOSPHOLIPID_METABOLIC_PROCESS	34	-0.45017	-1.44896	0.086242	1	0.921	2499	tags=38%, list=18%, signal=47%
DNA_METABOLIC_PROCESS	169	-0.42659	-1.44562	0.125514	1	0.924	3189	tags=44%, list=23%, signal=56%
DNA_REPAIR	83	-0.44888	-1.43731	0.122407	1	0.93	3085	tags=46%, list=23%, signal=59%
PROTEIN_DNA_COMPLEX_ASSEMBLY	35	-0.47914	-1.42807	0.124744	1	0.938	2625	tags=43%, list=19%, signal=53%
REGULATION_OF_MITOSIS	26	-0.53891	-1.42042	0.149901	1	0.943	2596	tags=54%, list=19%, signal=66%
M_PHASE	71	-0.49874	-1.41633	0.174699	0.979201	0.947	3072	tags=54%, list=23%, signal=69%
TRANSCRIPTION_INITIATION	23	-0.5176	-1.41132	0.108932	0.94973	0.95	1757	tags=39%, list=13%, signal=45%
DOUBLE_STRAND_BREAK_REPAIR	18	-0.50342	-1.39604	0.114345	0.978372	0.956	1558	tags=39%, list=11%, signal=44%
MRNA_PROCESSING_GO_0006397	50	-0.47793	-1.37971	0.153684	1	0.966	2536	tags=48%, list=19%, signal=59%
**Biological Process (LFGC)**								
NAME	SIZE	ES	NES	NOM p-val	FDR q-val	FWER p-val	RANK AT MAX	LEADING EDGE
IMMUNE_RESPONSE	145	0.61873	1.97102	0.004	0.09	0.063	2401	tags=56%, list=18%,

			4	283	9877			signal=67%
DEFENSE_RESPONSE	173	0.571424	1.936295	0.008869	0.082701	0.088	2461	tags=51%, list=18%, signal=61%
IMMUNE_SYSTEM_PROCESS	207	0.576359	1.8863	0.008811	0.11421	0.152	2401	tags=52%, list=18%, signal=62%
CYCLIC_NUCLEOTIDE_MEDIATED_SIGNALING	43	0.538298	1.886154	0	0.08635	0.154	1277	tags=40%, list=9%, signal=43%
G_PROTEIN_SIGNALING_COUPLED_TO_CYCLIC_NUCLEOTIDE_SECOND_MESSENGER	42	0.540901	1.846746	0.007444	0.112183	0.217	1277	tags=40%, list=9%, signal=45%
REGULATION_OF_PROTEIN_AMINO_ACID_PHOSPHORYLATION	18	0.65502	1.839378	0	0.10229	0.23	1422	tags=44%, list=10%, signal=50%
RESPONSE_TO_OTHER_ORGANISM	37	0.633649	1.831636	0.006637	0.09651	0.243	2458	tags=57%, list=18%, signal=69%
MULTI_ORGANISM_PROCESS	77	0.505827	1.830873	0.006494	0.085133	0.246	2699	tags=47%, list=20%, signal=58%
CELLULAR_DEFENSE_RESPONSE	38	0.667433	1.82391	0.013667	0.083166	0.265	1947	tags=55%, list=14%, signal=64%
CAMP_MEDIATED_SIGNALING	29	0.567475	1.821902	0	0.076653	0.268	2709	tags=55%, list=20%, signal=69%
BEHAVIOR	84	0.527808	1.820751	0.013699	0.070419	0.268	2395	tags=46%, list=18%, signal=56%
LOCOMOTORY_BEHAVIOR	64	0.595132	1.808114	0.013393	0.073622	0.298	2395	tags=56%, list=18%, signal=68%
G_PROTEIN_SIGNALING_COUPLED_TO_CAMP_NUCLEOTIDE_SECOND_MESSENGER	28	0.572484	1.791727	0.002353	0.081384	0.33	2709	tags=57%, list=20%, signal=71%
JAK_STAT_CASCADE	22	0.614424	1.790257	0.007737	0.077138	0.332	2370	tags=55%, list=17%, signal=66%
POSITIVE_REGULATION_OF_MULTICELLULAR_ORGANISMAL_PROCESS	39	0.55981	1.78999	0.01073	0.072088	0.332	2401	tags=49%, list=18%, signal=59%
IMMUNE_EFFECTOR_PROCESS	23	0.648251	1.78198	0.008	0.073971	0.348	1376	tags=57%, list=10%, signal=63%
SECOND_MESSENGER_MEDIATED_SIGNALING	74	0.478572	1.759456	0.007177	0.088508	0.403	1277	tags=31%, list=9%, signal=34%
INFLAMMATORY_RESPONSE	90	0.536287	1.749392	0.027837	0.09205	0.418	1608	tags=39%, list=12%, signal=44%
PEPTIDYL_TYROSINE_PHOSPHORYLATION	19	0.662132	1.74779	0.01227	0.088396	0.42	2370	tags=47%, list=17%, signal=57%
REGULATION_OF_IMMUNE_SYSTEM_PROCESS	39	0.561961	1.72683	0.02766	0.10236	0.472	2401	tags=51%, list=18%, signal=62%
**KEGG Pathway (HFGC)**								
NAME	SIZE	ES	NES	NOM p-val	FDR q-	FWER p-	RANK	LEADING EDGE

					val	val	AT MAX	
KEGG_GLYCOSYLPHOSPHATIDYLINOSITOL_GPI_ANCHOR_BIOSYNTHESIS	17	-0.6601	-1.68827	0.013699	0.913268	0.488	2499	tags=53%, list=18%, signal=65%
KEGG_MISMATCH_REPAIR	20	-0.67839	-1.6523	0.030675	0.623505	0.557	3127	tags=65%, list=23%, signal=84%
KEGG_DNA_REPLICATION	30	-0.69259	-1.58928	0.061475	0.683482	0.697	1140	tags=53%, list=8%, signal=58%
KEGG_N_GLYCAN_BIOSYNTHESIS	35	-0.478	-1.51036	0.0625	0.854995	0.814	3252	tags=51%, list=24%, signal=67%
KEGG_PROTEIN_EXPORT	21	-0.54417	-1.4696	0.092	0.860041	0.86	3078	tags=52%, list=23%, signal=68%
KEGG_NUCLEOTIDE_EXCISION_REPAIR	36	-0.49862	-1.43636	0.128846	0.861759	0.895	3127	tags=47%, list=23%, signal=61%
KEGG_BASE_EXCISION_REPAIR	23	-0.53646	-1.41653	0.124498	0.817245	0.904	3127	tags=61%, list=23%, signal=79%
KEGG_THYROID_CANCER	21	-0.44684	-1.35522	0.112936	0.94311	0.941	4477	tags=67%, list=33%, signal=99%
KEGG_CELL_CYCLE	92	-0.46083	-1.35385	0.196787	0.843535	0.941	1726	tags=41%, list=13%, signal=47%
KEGG_HOMOLOGOUS_RECOMBINATION	17	-0.56311	-1.32721	0.182	0.848409	0.96	3127	tags=47%, list=23%, signal=61%
KEGG_SPLICEOSOME	95	-0.45813	-1.31074	0.216216	0.825949	0.968	3377	tags=45%, list=25%, signal=60%
KEGG_PYRIMIDINE_METABOLISM	65	-0.3794	-1.22892	0.249012	1	0.994	3208	tags=38%, list=24%, signal=50%
KEGG_AMINOACYL_TRNA_BIOSYNTHESIS	32	-0.44189	-1.2235	0.2818	0.959943	0.995	4371	tags=56%, list=32%, signal=83%
KEGG_PROGESTERONE_MEDIATED_OOCYTE_MATURATION	54	-0.3868	-1.2147	0.276423	0.916797	0.995	2871	tags=46%, list=21%, signal=58%
KEGG_BIOSYNTHESIS_OF_UNSATURATED_FATTY_ACIDS	17	-0.4515	-1.20649	0.27821	0.878629	0.995	4881	tags=71%, list=36%, signal=110%
KEGG_BASAL_TRANSCRIPTION_FACTORS	25	-0.40496	-1.17575	0.26839	0.912466	0.998	2431	tags=44%, list=18%, signal=53%
KEGG_LYSINE_DEGRADATION	30	-0.40296	-1.1726	0.278	0.866963	0.998	3032	tags=40%, list=22%, signal=51%
KEGG_RNA_POLYMERASE	23	-0.44177	-1.16567	0.305785	0.836076	0.998	3208	tags=43%, list=24%, signal=57%
KEGG_ADHERENS_JUNCTION	55	-0.33143	-1.10704	0.324268	0.939998	1	1869	tags=27%, list=14%, signal=31%
KEGG_OOCYTE_MEIOSIS	75	-0.33657	-1.09607	0.376426	0.91905	1	2886	tags=35%, list=21%, signal=44%

					3			
**KEGG Pathway (LFGC)**								
NAME	SIZE	ES	NES	NOM p-val	FDR q-val	FWER p-val	RANK AT MAX	LEADING EDGE
KEGG_HEMATOPOIETIC_CELL_LINEAGE	47	0.704545	2.007128	0.00655	0.054984	0.027	1584	tags=53%, list=12%, signal=60%
KEGG_CYTOKINE_CYTOKINE_RECEPTOR_INTERACTION	139	0.561605	1.909341	0.009302	0.103436	0.104	2512	tags=50%, list=18%, signal=60%
KEGG_JAK_STAT_SIGNALING_PATHWAY	81	0.548258	1.882923	0.010438	0.094065	0.136	1689	tags=42%, list=12%, signal=48%
KEGG_INTESTINAL_IMMUNE_NETWORK_FOR_IGA_PRODUCTION	27	0.734203	1.830915	0.00655	0.132395	0.201	2957	tags=78%, list=22%, signal=99%
KEGG_PRION_DISEASES	30	0.602044	1.797722	0.008421	0.145171	0.259	2395	tags=50%, list=18%, signal=61%
KEGG_CHEMOKINE_SIGNALING_PATHWAY	132	0.509207	1.776465	0.029787	0.148752	0.3	1422	tags=36%, list=10%, signal=39%
KEGG_LEISHMANIA_INFECTION	47	0.652137	1.775539	0.02079	0.129828	0.305	2981	tags=68%, list=22%, signal=87%
KEGG_NATURAL_KILLER_CELL_MEDIATED_CYTOTOXICITY	75	0.567174	1.768625	0.039014	0.12248	0.318	2461	tags=49%, list=18%, signal=60%
KEGG_CALCIUM_SIGNALING_PATHWAY	93	0.457141	1.748602	0.009153	0.132013	0.367	3143	tags=44%, list=23%, signal=57%
KEGG_TOLL_LIKE_RECEPTOR_SIGNALING_PATHWAY	63	0.525927	1.713769	0.030675	0.161496	0.423	1631	tags=40%, list=12%, signal=45%
KEGG_CELL_ADHESION_MOLECULES_CAMS	94	0.515669	1.71064	0.037946	0.151088	0.435	2493	tags=48%, list=18%, signal=58%
KEGG_TYPE_I_DIABETES_MELLITUS	25	0.675559	1.680333	0.036797	0.176451	0.488	3079	tags=72%, list=23%, signal=93%
KEGG_GRAFT_VERSUS_HOST_DISEASE	24	0.746227	1.672133	0.044681	0.173156	0.501	2493	tags=79%, list=18%, signal=97%
KEGG_NEUROACTIVE_LIGAND_RECEPTOR_INTERACTION	107	0.434694	1.658422	0.005115	0.177866	0.53	2111	tags=36%, list=15%, signal=43%
KEGG_PRIMARY_IMMUNODEFICIENCY	24	0.675547	1.627136	0.048523	0.211331	0.59	1938	tags=63%, list=14%, signal=73%
KEGG_B_CELL_RECEPTOR_SIGNALING_PATHWAY	55	0.519313	1.609815	0.067762	0.225019	0.629	1631	tags=40%, list=12%, signal=45%
KEGG_ALLOGRAFT_REJECTION	20	0.745882	1.606325	0.046512	0.217333	0.633	3079	tags=90%, list=23%, signal=116%
KEGG_AUTOIMMUNE_THYROID_DISEASE	22	0.70187	1.59605	0.060606	0.220174	0.654	3079	tags=82%, list=23%, signal=106%

KEGG_LEUKOCYTE_TRANSENDOTHELIAL_MIGRATION	77	0.45788	1.586102	0.072581	0.223359	0.68	2254	tags=40%, list=17%, signal=48%
KEGG_APOPTOSIS	62	0.467115	1.554045	0.05157	0.261476	0.728	3053	tags=45%, list=22%, signal=58%
**Oncogenic (HFGC)**								
NAME	SIZE	ES	NES	NOM p-val	FDR q-val	FWER p-val	RANK AT MAX	LEADING EDGE
GCNP_SHH_UP_EARLY.V1_UP	112	-0.44558	-1.61302	0.022945	0.570273	0.455	2893	tags=47%, list=21%, signal=60%
RB_P107_DN.V1_UP	94	-0.52063	-1.57264	0.081439	0.395183	0.518	2187	tags=47%, list=16%, signal=55%
RB_DN.V1_UP	91	-0.46223	-1.53291	0.055985	0.351861	0.583	3121	tags=45%, list=23%, signal=58%
RPS14_DN.V1_DN	128	-0.43877	-1.51859	0.066288	0.2904	0.609	2817	tags=41%, list=21%, signal=52%
HOXA9_DN.V1_DN	131	-0.39457	-1.49063	0.052632	0.280516	0.67	1376	tags=24%, list=10%, signal=27%
RB_P130_DN.V1_UP	73	-0.40246	-1.44796	0.06203	0.305194	0.726	3345	tags=42%, list=25%, signal=56%
GCNP_SHH_UP_LATE.V1_UP	128	-0.38965	-1.42891	0.093458	0.294296	0.747	2842	tags=43%, list=21%, signal=54%
YAP1_DN	24	-0.43614	-1.38845	0.108987	0.325464	0.791	1761	tags=33%, list=13%, signal=38%
SRC_UP.V1_DN	105	-0.34118	-1.31503	0.109185	0.428184	0.869	3483	tags=41%, list=26%, signal=55%
MTOR_UP.V1_UP	107	-0.33723	-1.30871	0.155009	0.399462	0.885	2733	tags=34%, list=20%, signal=42%
E2F3_UP.V1_UP	102	-0.3626	-1.30344	0.166963	0.373698	0.888	3872	tags=45%, list=28%, signal=63%
PRC2_EZH2_UP.V1_UP	109	-0.36104	-1.29241	0.179487	0.362678	0.9	2825	tags=38%, list=21%, signal=47%
MYC_UP.V1_UP	106	-0.34623	-1.28213	0.174147	0.352583	0.908	3044	tags=35%, list=22%, signal=45%
E2F1_UP.V1_UP	119	-0.37075	-1.21113	0.30916	0.458367	0.953	3075	tags=41%, list=23%, signal=53%
CSR_LATE_UP.V1_UP	125	-0.37744	-1.20792	0.306569	0.433931	0.954	3417	tags=42%, list=25%, signal=56%
EGFR_UP.V1_DN	136	-0.29014	-1.19597	0.196	0.430325	0.959	2466	tags=26%, list=18%, signal=31%
AKT_UP_MTOR_DN.V1_DN	117	-0.2773	-1.11006	0.307692	0.57449	0.979	2660	tags=27%, list=20%, signal=34%

					9			
GLI1_UP.V1_DN	23	-0.38021	-1.07906	0.381481	0.613293	0.983	2637	tags=35%, list=19%, signal=43%
VEGF_A_UP.V1_DN	145	-0.29105	-1.05405	0.385338	0.637762	0.987	3072	tags=34%, list=23%, signal=43%
TBK1.DN.48HRS_UP	37	-0.32568	-1.04908	0.412863	0.617005	0.988	4923	tags=57%, list=36%, signal=89%
**Oncogenic (LFGC)**								
NAME	SIZE	ES	NES	NOM p-val	FDR q-val	FWER p-val	RANK AT MAX	LEADING EDGE
P53_DN.V1_DN	132	0.498719	1.865869	0	0.146619	0.072	2879	tags=48%, list=21%, signal=60%
PTEN_DN.V2_UP	94	0.478087	1.825921	0	0.122728	0.104	2384	tags=40%, list=17%, signal=49%
HOXA9_DN.V1_UP	145	0.532434	1.818311	0.004107	0.086655	0.11	3358	tags=52%, list=25%, signal=69%
STK33_NOMO_UP	225	0.491031	1.809651	0.010395	0.073094	0.118	3463	tags=52%, list=25%, signal=68%
RPS14_DN.V1_UP	136	0.581069	1.798718	0.014675	0.06671	0.13	2856	tags=57%, list=21%, signal=71%
RELA_DN.V1_UP	86	0.457427	1.758811	0.002141	0.088525	0.176	2304	tags=41%, list=17%, signal=49%
STK33_SKM_UP	211	0.475827	1.739224	0.012793	0.091894	0.205	2843	tags=47%, list=21%, signal=58%
EGFR_UP.V1_UP	149	0.473234	1.737659	0.010417	0.082116	0.208	2460	tags=41%, list=18%, signal=49%
STK33_UP	221	0.473561	1.731673	0.02079	0.0773	0.216	2577	tags=42%, list=19%, signal=51%
KRAS.LUNG_UP.V1_UP	74	0.461387	1.71326	0.006682	0.083486	0.241	3118	tags=42%, list=23%, signal=54%
VEGF_A_UP.V1_UP	99	0.445195	1.704662	0.008403	0.082356	0.26	2205	tags=34%, list=16%, signal=41%
P53_DN.V2_UP	73	0.501725	1.699962	0.025229	0.079479	0.272	2736	tags=45%, list=20%, signal=56%
LEF1_UP.V1_UP	109	0.475131	1.698026	0.008949	0.075302	0.275	2520	tags=41%, list=18%, signal=50%
MEL18_DN.V1_DN	99	0.454059	1.687313	0.008333	0.078173	0.289	2586	tags=41%, list=19%, signal=51%
IL2_UP.V1_UP	118	0.43754	1.622774	0.025114	0.130928	0.403	2605	tags=37%, list=19%, signal=46%
BCAT_GDS748_UP	35	0.46845	1.60452	0.020	0.14	0.442	1610	tags=29%, list=12%,

				325	197 4			signal=32%
KRAS.BREAST_UP.V1_ UP	68	0.45164	1.60253 1	0.043 373	0.13 565 1	0.443	2676	tags=41%, list=20%, signal=51%
PTEN_DN.V1_UP	103	0.39776 4	1.60025 6	0.019 093	0.12 977 9	0.445	2812	tags=38%, list=21%, signal=47%
BMI1_DN_MEL18_DN.V 1_DN	98	0.41994 3	1.58354 6	0.024 59	0.13 773 4	0.471	2432	tags=37%, list=18%, signal=44%
BMI1_DN.V1_DN	87	0.38760 4	1.55729	0.018 908	0.15 855 6	0.526	2472	tags=33%, list=18%, signal=40%

Table S6. GSEA result compared HFGC with LFGC of TCGA HCC

Biological Process (HFGC)								
NAME	SIZE	ES	NES	NOM p-val	FDR q-val	FWER p-val	RANK AT MAX	LEADING EDGE
CHROMOSOME_SEGREGATION	29	-0.7256807	-2.1769378	0.0021142	0.0108505	0.005	509	tags=45%, list=3%, signal=46%
M_PHASE	101	-0.6577637	-2.1104443	0.0039841	0.0159817	0.015	2517	tags=49%, list=15%, signal=57%
DNA_DEPENDENT_DNA_REPLICATION	51	-0.6878951	-2.1082685	0	0.011113	0.016	1911	tags=47%, list=12%, signal=53%
REGULATION_OF_MITOSIS	36	-0.7227784	-2.103995	0.0020325	0.0094778	0.018	441	tags=39%, list=3%, signal=40%
CELL_CYCLE_PROCESS	175	-0.6020439	-2.098091	0.0060606	0.0081045	0.021	1896	tags=39%, list=11%, signal=44%
DNA_REPAIR	110	-0.5683734	-2.078699	0	0.0090214	0.03	1911	tags=35%, list=12%, signal=39%
CELL_CYCLE_PHASE	153	-0.5939316	-2.0732765	0.0080645	0.0082688	0.033	2517	tags=42%, list=15%, signal=49%
DNA_METABOLIC_PROCESS	229	-0.5257379	-2.0633385	0.0040404	0.0084337	0.037	1911	tags=32%, list=12%, signal=36%
DNA_REPLICATION	93	-0.5967884	-2.0442684	0.0041494	0.0088635	0.046	1911	tags=38%, list=12%, signal=42%
M_PHASE_OF_MITOTIC_CELL_CYCLE	76	-0.6681321	-2.0280175	0.0082988	0.0103759	0.057	589	tags=36%, list=4%, signal=37%
MITOSIS	73	-0.6653343	-2.0132692	0.008316	0.0105645	0.066	589	tags=36%, list=4%, signal=37%
RESPONSE_TO_DNA_DAMAGE_STIMULUS	143	-0.5180857	-2.0058506	0.0020492	0.0105438	0.072	2706	tags=37%, list=16%, signal=44%
REGULATION_OF_GENE_EXPRESSION_EPIGENETIC	28	-0.620373	-1.9912269	0	0.0115952	0.083	1359	tags=36%, list=8%, signal=39%
MEIOTIC_CELL_CYCLE	30	-0.6747697	-1.977847	0	0.01243	0.089	2517	tags=53%, list=15%,

					48			signal=63%
RESPONSE_TO_ENDOGENOUS_STIMULUS	178	-0.4732107	-1.9758451	0	0.0118177	0.089	3235	tags=38%, list=20%, signal=46%
ORGANELLE_LOCALIZATION	23	-0.6114302	-1.9455433	0	0.0161809	0.132	441	tags=30%, list=3%, signal=31%
CELL_CYCLE_CHECKPOINT_GO_0000075	42	-0.6341423	-1.9398936	0.01	0.0165203	0.144	2706	tags=52%, list=16%, signal=62%
MITOTIC_CELL_CYCLE	138	-0.5599372	-1.93874	0.0199203	0.0157299	0.145	1849	tags=34%, list=11%, signal=38%
CELL_CYCLE_GO_0007049	284	-0.4878363	-1.9308627	0.0099404	0.0165395	0.16	1956	tags=30%, list=12%, signal=33%
MEIOSIS_I	17	-0.7186291	-1.9257898	0.001996	0.0167939	0.167	1504	tags=53%, list=9%, signal=58%
**Biological Process (LFGC)**								
NAME	SIZE	ES	NES	NOM p-val	FDR q-val	FWER p-val	RANK AT MAX	LEADING EDGE
BONE_REMODELING	28	0.5837959	1.8827065	0.0022676	0.8077714	0.242	2183	tags=39%, list=13%, signal=45%
G_PROTEIN_SIGNALING_COUPLED_TO_CYCLIC_NUCLEOTIDE_SECOND_MESSENGER	94	0.4412195	1.8440132	0	0.6043246	0.326	3773	tags=40%, list=23%, signal=52%
CYCLIC_NUCLEOTIDE_MEDIATED_SIGNALING	96	0.4327054	1.8185772	0	0.5129932	0.403	3773	tags=40%, list=23%, signal=51%
SECOND_MESSENGER_MEDIATED_SIGNALING	142	0.4236116	1.7985157	0	0.4633081	0.457	3773	tags=39%, list=23%, signal=51%
TISSUE_REMODELING	29	0.547134	1.7777083	0.0022676	0.4474539	0.509	2183	tags=38%, list=13%, signal=44%
G_PROTEIN_SIGNALING_COUPLED_TO_CAMP_NUCLEOTIDE_SECOND_MESSENGER	62	0.4338301	1.7289587	0.0043764	0.5601189	0.64	3773	tags=40%, list=23%, signal=52%
CAMP_MEDIATED_SIGNALING	63	0.429736	1.7278168	0.0044346	0.4838306	0.643	3773	tags=40%, list=23%, signal=51%

REGULATION_OF_HEART_CONTRACTION	24	0.5629414	1.7137011	0.0241228	0.4789264	0.68	3060	tags=46%, list=18%, signal=56%
RESPONSE_TO_VIRUS	37	0.6080874	1.7080084	0.0308285	0.4441279	0.695	2834	tags=54%, list=17%, signal=65%
ENERGY_DERIVATION_BY_OXIDATION_OF_ORGANIC_COMPOUNDS	35	0.5616012	1.6965986	0.0219124	0.4350027	0.726	3566	tags=51%, list=22%, signal=65%
G_PROTEIN_SIGNALING_COUPLED_TO_IP3_SECOND_MESSENGERPHOSPHOLIPASE_C_ACTIVATING	43	0.4625911	1.6809778	0.0045147	0.447155	0.765	3406	tags=40%, list=21%, signal=50%
PHOSPHOINOSITIDE_MEDIATED_SIGNALING	46	0.4494167	1.6508359	0.0067416	0.5164834	0.818	3406	tags=39%, list=21%, signal=49%
CELL_SUBSTRATE_ADHESION	38	0.4938582	1.6485391	0.0216963	0.4853369	0.823	3722	tags=47%, list=22%, signal=61%
RESPONSE_TO_OTHER_ORGANISM	65	0.5113334	1.607881	0.0425963	0.5987135	0.888	4209	tags=54%, list=25%, signal=72%
AXON_GUIDANCE	20	0.546113	1.5928898	0.0367171	0.616441	0.914	1687	tags=40%, list=10%, signal=44%
CELL_MATRIX_ADHESION	37	0.4754125	1.5913014	0.0357143	0.5845706	0.915	3722	tags=46%, list=22%, signal=59%
INSULIN_RECEPTOR_SIGNALING_PATHWAY	17	0.5940896	1.5898261	0.0471311	0.5567232	0.916	4604	tags=65%, list=28%, signal=89%
RESPONSE_TO_BIOTIC_STIMULUS	101	0.4569852	1.5837369	0.0359408	0.5467475	0.922	3423	tags=43%, list=21%, signal=53%
SKELETAL_DEVELOPMENT	95	0.4345817	1.5740607	0.0569476	0.5514554	0.931	3553	tags=44%, list=21%, signal=56%
CALCIUM_ION_TRANSPORT	25	0.5010728	1.5728935	0.0242291	0.5276492	0.932	3870	tags=44%, list=23%, signal=57%
**KEGG Pathway (HFGC)**								
NAME	SIZE	ES	NES	NOM p-val	FDR q-val	FWER p-val	RANK AT MAX	LEADING EDGE
KEGG_HOMOLOGOUS_RECOMBINATION	27	-0.7821631	-2.0921094	0	0.00834	0.013	1419	tags=52%, list=9%,

					65			signal=57%
KEGG_MISMATCH_REPAIR	22	-0.7855697	-1.9719963	0.0020833	0.0225981	0.059	2093	tags=55%, list=13%, signal=62%
KEGG_CELL_CYCLE	115	-0.5960533	-1.9206722	0.006237	0.0272204	0.09	2339	tags=47%, list=14%, signal=54%
KEGG_DNA_REPLICATION	33	-0.8120576	-1.9124084	0	0.0227869	0.096	570	tags=58%, list=3%, signal=60%
KEGG_BASE_EXCISION_REPAIR	30	-0.6350449	-1.8123506	0.0081301	0.0599964	0.239	2604	tags=53%, list=16%, signal=63%
KEGG_PYRIMIDINE_METABOLISM	84	-0.457888	-1.648735	0.0167364	0.2059161	0.615	1291	tags=25%, list=8%, signal=27%
KEGG_SPLICEOSOME	116	-0.5450181	-1.6371747	0.0433925	0.1944765	0.642	3614	tags=52%, list=22%, signal=66%
KEGG_OOCYTE_MEIOSIS	101	-0.4245597	-1.6295837	0.0168224	0.1786417	0.66	976	tags=22%, list=6%, signal=23%
KEGG_NUCLEOTIDE_EXCISION_REPAIR	39	-0.5257825	-1.6200255	0.0351563	0.1698185	0.684	2143	tags=33%, list=13%, signal=38%
KEGG_N_GLYCAN_BIOSYNTHESIS	42	-0.4592321	-1.5965395	0.0368664	0.181347	0.733	2905	tags=38%, list=18%, signal=46%
KEGG_MATURITY_ONSET_DIABETES_OF_THE_YOUNG	25	-0.5502435	-1.5653161	0.0581162	0.2020812	0.794	3270	tags=44%, list=20%, signal=55%
KEGG_BASAL_TRANSCRIPTION_FACTORS	31	-0.4453705	-1.4513489	0.082996	0.3563504	0.934	2762	tags=32%, list=17%, signal=39%
KEGG_UBIQUITIN_MEDIATED_PROTEOLYSIS	119	-0.3430879	-1.4071141	0.0550847	0.4076045	0.969	3056	tags=29%, list=18%, signal=35%
KEGG_PROGESTERONE_MEDIATED_OOCYTE_MATURATION	77	-0.3630972	-1.3535997	0.1175337	0.4821322	0.987	2171	tags=27%, list=13%, signal=31%
KEGG_P53_SIGNALING_PATHWAY	60	-0.3579201	-1.3314016	0.1078838	0.4952371	0.99	2250	tags=28%, list=14%, signal=33%
KEGG_PENTOSE_AND_GLUCURONATE_INTERCONVERSIONS	26	-0.520304	-1.2967172	0.2302632	0.5338726	0.993	4224	tags=46%, list=25%, signal=62

								%
KEGG_BLADDER_CANCER	37	-0.3534546	-1.1797932	0.244898	0.7698757	1	4124	tags=43%, list=25%, signal=57%
KEGG_THYROID_CANCER	28	-0.351849	-1.1598387	0.2665289	0.7766116	1	2043	tags=21%, list=12%, signal=24%
KEGG_TERPENOID_BACKBONE_BIOSYNTHESIS	15	-0.4792478	-1.1281612	0.3621053	0.8139191	1	1813	tags=40%, list=11%, signal=45%
KEGG_RIBOSOME	80	-0.5202168	-1.118066	0.4309278	0.7972894	1	5320	tags=66%, list=32%, signal=97%
**KEGG Pathway (LFGC)**								
NAME	SIZE	ES	NES	NOM p-val	FDR q-val	FWER p-val	RANK AT MAX	LEADING EDGE
KEGG_CALCIUM_SIGNALING_PATHWAY	155	0.4612967	1.9231007	0	0.2147519	0.101	3474	tags=39%, list=21%, signal=49%
KEGG_REGULATION_OF_AUTOPHAGY	19	0.5696816	1.750542	0.0102041	0.7194228	0.397	1729	tags=32%, list=10%, signal=35%
KEGG_VASCULAR_SMOOTH_MUSCLE_CONTRACTION	106	0.4589802	1.7177393	0.0108225	0.6369793	0.471	2705	tags=37%, list=16%, signal=44%
KEGG_VALINE_LEUCINE_AND_ISOLEUCINE_DEGRADATION	42	0.6580745	1.6309192	0.0534653	0.9256749	0.673	2805	tags=60%, list=17%, signal=71%
KEGG_HEMATOPOIETIC_CELL_LINEAGE	80	0.4910937	1.6131573	0.0688259	0.8442543	0.715	4248	tags=52%, list=26%, signal=70%
KEGG_GLYCOSAMINOGLYCAN_BIOSYNTHESIS_CHONDROITIN_SULFATE	21	0.5366713	1.5693448	0.0434783	0.927301	0.803	1176	tags=38%, list=7%, signal=41%
KEGG_CYTOKINE_CYTOKINE_RECEPTOR_INTERACTION	226	0.4284237	1.5615697	0.0597015	0.8342742	0.815	4104	tags=43%, list=25%, signal=56%
KEGG_APOPTOSIS	78	0.4165952	1.5483484	0.0372549	0.790282	0.836	3311	tags=37%, list=20%, signal=46%
KEGG_DILATED_CARDIOMYOPATHY	82	0.4527537	1.5069128	0.0651163	0.8841164	0.891	3267	tags=41%, list=20%, signal=51%

KEGG_NICOTINATE_AND_NICOTINAMIDE_METABOLISM	22	0.4871063	1.4966484	0.05665657	0.8410843	0.896	5949	tags=64%, list=36%, signal=99%
KEGG_NEUROACTIVE_LIGAND_RECEPTOR_INTERACTION	229	0.3238937	1.4924084	0.0221607	0.7820019	0.901	3755	tags=31%, list=23%, signal=40%
KEGG_B_CELL_RECEPTOR_SIGNALING_PATHWAY	71	0.4492866	1.4923524	0.0848485	0.7168351	0.901	2989	tags=41%, list=18%, signal=50%
KEGG_CITRATE_CYCLE_TCA_CYCLE	28	0.573049	1.485822	0.0989899	0.6881833	0.909	3566	tags=54%, list=22%, signal=68%
KEGG_CHEMOKINE_SIGNALING_PATHWAY	179	0.403439	1.4697447	0.0907173	0.6943442	0.925	4240	tags=45%, list=26%, signal=60%
KEGG_ETHER_LIPID_METABOLISM	30	0.4054771	1.4601877	0.0378947	0.6810028	0.938	1951	tags=30%, list=12%, signal=34%
KEGG_NATURAL_KILLER_CELL_MEDIATED_CYTOTOXICITY	111	0.4265771	1.4512093	0.1234568	0.6678835	0.944	3561	tags=43%, list=21%, signal=55%
KEGG_JAK_STAT_SIGNALING_PATHWAY	122	0.3924675	1.4459618	0.0817204	0.6446661	0.949	3064	tags=32%, list=18%, signal=39%
KEGG_CELL_ADHESION_MOLECULES_CAMS	115	0.4258497	1.4085304	0.1283644	0.7238066	0.968	3303	tags=41%, list=20%, signal=51%
KEGG_ECM_RECEPTOR_INTERACTION	79	0.4428824	1.4052496	0.169697	0.6949843	0.97	3400	tags=46%, list=21%, signal=57%
KEGG_LEISHMANIA_INFECTION	63	0.4759485	1.3842937	0.1816367	0.7285357	0.976	3303	tags=43%, list=20%, signal=53%
**Oncogenic (HFGC)**								
NAME	SIZE	ES	NES	NOM p-val	FDR q-val	FWER p-val	RANK AT MAX	LEADING EDGE
RPS14_DN.V1_DN	163	-0.5448914	-2.1645298	0	0	0	1825	tags=33%, list=11%, signal=37%
PRC2_EZH2_UP.V1_UP	165	-0.4864141	-2.0560021	0	8.97E-04	0.005	886	tags=27%, list=5%, signal=28%
E2F1_UP.V1_UP	173	-0.4767531	-1.8829107	0.0037951	0.0119927	0.072	2545	tags=35%, list=15%, signal=41

								%
RB_P107_DN.V1_UP	125	-0.5648047	-1.833929	0.0201613	0.0170637	0.114	1595	tags=42%, list=10%, signal=47%
RB_DN.V1_UP	119	-0.4355741	-1.7348772	0.0061856	0.0374932	0.227	2465	tags=33%, list=15%, signal=38%
GCNP_SHH_UP_EARLY.V1_UP	152	-0.4522155	-1.7185619	0.0174757	0.0363483	0.251	3025	tags=36%, list=18%, signal=44%
CSR_LATE_UP.V1_UP	152	-0.4781577	-1.688554	0.0246212	0.0403553	0.31	2151	tags=38%, list=13%, signal=43%
E2F3_UP.V1_UP	162	-0.3926694	-1.6751021	0.0058366	0.0394561	0.326	2796	tags=31%, list=17%, signal=37%
HOXA9_DN.V1_DN	164	-0.4006853	-1.6392138	0.0080321	0.0488003	0.412	3375	tags=37%, list=20%, signal=46%
GCNP_SHH_UP_LATE.V1_UP	160	-0.4417787	-1.6136187	0.0329457	0.0539783	0.465	1563	tags=25%, list=9%, signal=27%
PRC2_EDD_UP.V1_UP	171	-0.3744294	-1.5758784	0.0288066	0.0666854	0.558	2143	tags=26%, list=13%, signal=30%
MTOR_UP.V1_UP	153	-0.344041	-1.5182328	0.0316832	0.0948124	0.684	2817	tags=28%, list=17%, signal=34%
RB_P130_DN.V1_UP	116	-0.3776458	-1.5029774	0.0246407	0.0959503	0.711	2850	tags=32%, list=17%, signal=38%
SRC_UP.V1_DN	145	-0.3309808	-1.4914079	0.0149533	0.0968481	0.73	1739	tags=21%, list=10%, signal=24%
MYC_UP.V1_UP	146	-0.3925988	-1.4314686	0.1064718	0.1335323	0.838	4985	tags=49%, list=30%, signal=69%
VEGF_A_UP.V1_DN	176	-0.3885405	-1.419865	0.0994264	0.1346864	0.855	1489	tags=21%, list=9%, signal=23%
TBK1.DN.48HRS_UP	46	-0.397412	-1.419133	0.0611814	0.1273293	0.857	3288	tags=35%, list=20%, signal=43%
BCAT_GDS748_DN	40	-0.3437548	-1.3097826	0.1162325	0.2279908	0.96	3751	tags=45%, list=23%, signal=58

								%
NFE2L2.V2	381	-0.2793905	-1.2796575	0.1237525	0.2551636	0.976	3066	tags=24%, list=18%, signal=28%
DCA_UP.V1_DN	150	-0.2611077	-1.2338156	0.0963855	0.3085801	0.991	2724	tags=23%, list=16%, signal=27%
**Oncogenic (LFGC)**								
NAME	SIZE	ES	NES	NOM p-val	FDR q-val	FWER p-val	RANK AT MAX	LEADING EDGE
HOXA9_DN.V1_UP	171	0.519038	1.9048997	0.0039448	0.1010086	0.054	4414	tags=56%, list=27%, signal=75%
PTEN_DN.V1_UP	160	0.4476807	1.8342359	0.0022272	0.1234086	0.112	3479	tags=41%, list=21%, signal=52%
LEF1_UP.V1_UP	163	0.511918	1.8233509	0.0147059	0.0920985	0.12	3526	tags=47%, list=21%, signal=59%
RPS14_DN.V1_UP	179	0.5407417	1.773334	0.0308008	0.1211903	0.176	3525	tags=48%, list=21%, signal=60%
BMI1_DN_MEL18_DN.V1_DN	131	0.4469608	1.7732651	0	0.0969523	0.176	3682	tags=37%, list=22%, signal=48%
P53_DN.V1_DN	175	0.4529273	1.7417918	0.0100604	0.1107258	0.224	3867	tags=45%, list=23%, signal=58%
CAHOY_ASTROGLIAL	86	0.5048821	1.7266909	0.0229885	0.111695	0.252	2976	tags=44%, list=18%, signal=54%
ATF2_UP.V1_DN	171	0.4762719	1.7226397	0.014	0.1000324	0.254	3770	tags=49%, list=23%, signal=62%
KRAS.600_UP.V1_UP	236	0.3785581	1.7125484	0.0042373	0.0980176	0.268	3257	tags=33%, list=20%, signal=41%
MEL18_DN.V1_DN	132	0.4435261	1.708167	0.0039841	0.0913319	0.273	4001	tags=43%, list=24%, signal=56%
PRC2_EZH2_UP.V1_DN	158	0.4094486	1.6979431	0.0021097	0.09284	0.295	2820	tags=30%, list=17%, signal=36%

ATF2_S_UP.V1_DN	165	0.4447916	1.678042	0.02182 54	0.10172 7	0.328	368 2	tags=44%, list=22%, signal=56 %
SNF5_DN.V1_DN	144	0.398627	1.6590326	0.00199 2	0.11278 94	0.372	360 7	tags=38%, list=22%, signal=48 %
PTEN_DN.V2_UP	121	0.4029441	1.6550689	0.00801 6	0.10770 28	0.382	378 6	tags=40%, list=23%, signal=51 %
CYCLIN_D1_KE_.V1_DN	171	0.3794514	1.6550121	0.01054 85	0.10052 26	0.382	409 3	tags=40%, list=25%, signal=53 %
STK33_SKM_UP	245	0.4252608	1.6459278	0.02024 29	0.10044 87	0.398	284 5	tags=35%, list=17%, signal=41 %
BMI1_DN.V1_UP	131	0.4983971	1.6403983	0.03925 62	0.09909 74	0.409	281 7	tags=40%, list=17%, signal=48 %
AKT_UP.V1_DN	171	0.4042722	1.6097466	0.01500 94	0.11658 51	0.46	383 8	tags=43%, list=23%, signal=56 %
STK33_UP	256	0.4384923	1.6074026	0.02811 25	0.11168 33	0.464	433 5	tags=45%, list=26%, signal=59 %
KRAS.KIDNEY_UP.V1_UP	130	0.4079987	1.6069605	0.02469 14	0.10655 67	0.465	508 4	tags=53%, list=31%, signal=76 %

Table S7. Overlapped GSEA result HFGC and LFGC of Thai HCC and Thai iCCA

Name	Size	ES	NES	NOM.p.val	Size	ES	NES	NOM.p.val
	HCC_HFGC				iCCA_HFGC			
**Biological Process**								
DNA_DEPENDENT_DNA_REPLICATION	40	-0.6292	-1.76602	0.0221	43	-0.6745	-1.90739	0.002
CHROMOSOME_SEGREGATION	21	-0.6397	-1.63112	0.0593	21	-0.7025	-1.8006	0.0039
DNA_REPLICATION	70	-0.5365	-1.61436	0.0561	75	-0.5991	-1.80371	0.008
REGULATION_OF_MITOSIS	26	-0.5389	-1.42042	0.1499	26	-0.6822	-1.78362	0.008
M_PHASE	71	-0.4987	-1.41633	0.1747	70	-0.6641	-1.85491	0.004
**KEGG pathway**								
KEGG_GLYCOSYLPHOSPHATIDYLINOSITOL_GPI_ANCHOR_BIOSYNTHESIS	17	-0.6601	-1.68827	0.0137	18	-0.5825	-1.49526	0.0538
KEGG_MISMATCH_REPAIR	20	-0.6784	-1.6523	0.0307	19	-0.7417	-1.77585	0.0059
KEGG_DNA_REPLICATION	30	-0.6926	-1.58928	0.0615	29	-0.8124	-1.86008	0.0019
KEGG_N_GLYCAN_BIOSYNTHESIS	35	-0.478	-1.51036	0.0625	37	-0.5023	-1.56237	0.0462
KEGG_NUCLEOTIDE_EXCISION_REPAIR	36	-0.4986	-1.43636	0.1288	34	-0.5713	-1.58788	0.0355
KEGG_BASE_EXCISION_REPAIR	23	-0.5365	-1.41653	0.1245	21	-0.6762	-1.7393	0.0077
KEGG_CELL_CYCLE	92	-0.4608	-1.35385	0.1968	90	-0.6096	-1.72089	0.0211
KEGG_HOMOLOGOUS_RECOMBINATION	17	-0.5631	-1.32721	0.182	16	-0.7101	-1.68874	0.0134
KEGG_SPLICEOSOME	95	-0.4581	-1.31074	0.2162	96	-0.5311	-1.552	0.0825
KEGG_PYRIMIDINE_METABOLISM	65	-0.3794	-1.22892	0.249	62	-0.5061	-1.59065	0.0476
KEGG_AMINOACYL_TRNA_BIOSYNTHESIS	32	-0.4419	-1.2235	0.2818	30	-0.6253	-1.60137	0.0329
KEGG_PROGESTERONE_MEDIATED_OOCYTE_MATURATION	54	-0.3868	-1.2147	0.2764	54	-0.4414	-1.44729	0.0696

KEGG_BASAL_TRANSCRIPTION_FACTORS	25	-0.405	-1.17575	0.2684	24	-0.5638	-1.54818	0.0511
KEGG_RNA_POLYMERASE	23	-0.4418	-1.16567	0.3058	23	-0.6545	-1.64476	0.0059
KEGG_OOCYTE_MEIOSIS	75	-0.3366	-1.09607	0.3764	77	-0.4877	-1.61648	0.0206
**Oncogenic pathway**								
GCNP_SHH_UP_EARLY.V1_UP	112	-0.4456	-1.61302	0.0229	115	-0.4474	-1.65347	0.0189
RB_P107_DN.V1_UP	94	-0.5206	-1.57264	0.0814	88	-0.6059	-1.923	0.0059
RB_DN.V1_UP	91	-0.4622	-1.53291	0.056	89	-0.5635	-1.93142	0.0039
RPS14_DN.V1_DN	128	-0.4388	-1.51859	0.0663	132	-0.5152	-1.76369	0.0213
HOXA9_DN.V1_DN	131	-0.3946	-1.49063	0.0526	135	-0.4055	-1.53515	0.0503
RB_P130_DN.V1_UP	73	-0.4025	-1.44796	0.062	78	-0.4179	-1.61577	0.0203
GCNP_SHH_UP_LATE.V1_UP	128	-0.3897	-1.42891	0.0935	128	-0.3713	-1.39788	0.1212
E2F3_UP.V1_UP	102	-0.3626	-1.30344	0.167	105	-0.3547	-1.40734	0.0911
PRC2_EZH2_UP.V1_UP	109	-0.361	-1.29241	0.1795	117	-0.4752	-1.77755	0.0115
MYC_UP.V1_UP	106	-0.3462	-1.28213	0.1741	105	-0.4026	-1.32091	0.1899
E2F1_UP.V1_UP	119	-0.3708	-1.21113	0.3092	123	-0.5175	-1.75653	0.0179
CSR_LATE_UP.V1_UP	125	-0.3774	-1.20792	0.3066	125	-0.5026	-1.61629	0.0247
EGFR_UP.V1_DN	136	-0.2901	-1.19597	0.196	135	-0.3226	-1.39144	0.0588
VEGF_A_UP.V1_DN	145	-0.2911	-1.05405	0.3853	147	-0.3992	-1.44666	0.1016
TBK1.DN.48HRS_UP	37	-0.3257	-1.04908	0.4129	39	-0.4025	-1.34081	0.1044
	**HCC_LFGC**				**iCCA_LFGC**			
**Biological Process**								

IMMUNE_RESPONSE	145	0.6187	1.971024	0.0043	152	0.6352	1.852819	0.0233
DEFENSE_RESPONSE	173	0.5714	1.936295	0.0089	189	0.6299	1.883574	0.0162
IMMUNE_SYSTEM_PROCESS	207	0.5764	1.8863	0.0088	219	0.5803	1.845352	0.0307
RESPONSE_TO_OTHER_ORGANISM	37	0.6336	1.831636	0.0066	48	0.6462	1.828193	0.0161
MULTI_ORGANISM_PROCESS	77	0.5058	1.830873	0.0065	97	0.5187	1.767454	0.0182
CELLULAR_DEFENSE_RESPONSE	38	0.6674	1.82391	0.0137	40	0.6893	1.756464	0.0291
LOCOMOTORY_BEHAVIOR	64	0.5951	1.808114	0.0134	74	0.6033	1.772803	0.0164
POSITIVE_REGULATION_OF_MULTICELLULAR_ORGANISMAL_PROCESS	39	0.5598	1.78999	0.0107	42	0.6503	1.828494	0.0101
INFLAMMATORY_RESPONSE	90	0.5363	1.749392	0.0278	100	0.6446	1.858764	0.0142
REGULATION_OF_IMMUNE_SYSTEM_PROCESS	39	0.562	1.72683	0.0277	39	0.6153	1.72256	0.0339
**KEGG pathway**								
KEGG_HEMATOPOIETIC_CELL_LINEAGE	47	0.7045	2.007128	0.0066	49	0.7264	1.866346	0.002
KEGG_CYTOKINE_CYTOKINE_RECEPTOR_INTERACTION	139	0.5616	1.909341	0.0093	156	0.5901	1.843299	0.002
KEGG_JAK_STAT_SIGNALING_PATHWAY	81	0.5483	1.882923	0.0104	84	0.4794	1.554089	0.0713
KEGG_INTESTINAL_IMMUNE_NETWORK_FOR_IGA_PRODUCTION	27	0.7342	1.830915	0.0066	29	0.6359	1.499596	0.1064
KEGG_PRION_DISEASES	30	0.602	1.797722	0.0084	29	0.5504	1.459598	0.092
KEGG_CHEMOKINE_SIGNALING_PATHWAY	132	0.5092	1.776465	0.0298	142	0.458	1.585065	0.0538
KEGG_NATURAL_KILLER_CELL_MEDIATED_CYTOTOXICITY	75	0.5672	1.768625	0.039	80	0.5364	1.660275	0.0455
KEGG_CELL_ADHESION_MOLECULES_CAMS	94	0.5157	1.71064	0.0379	93	0.5438	1.637101	0.052
KEGG_GRAFT_VERSUS_HOST_DISEASE	24	0.7462	1.672133	0.0447	25	0.7296	1.496257	0.0682
KEGG_NEUROACTIVE_LIGAND_RECEPTOR_INTERACTION	107	0.4347	1.658422	0.0051	108	0.4393	1.640409	0.0165
KEGG_PRIMARY_IMMUNODEFICIENCY	24	0.6755	1.627136	0.0485	26	0.6269	1.495657	0.1086
KEGG_B_CELL_RECEPTOR_SIGNALING_PATHWAY	55	0.5193	1.609815	0.0678	58	0.4919	1.570837	0.0593
KEGG_AUTOIMMUNE_THYROID_DISEASE	22	0.7019	1.59605	0.0606	23	0.6945	1.483169	0.0969
KEGG_LEUKOCYTE_TRANSENDOTHELIAL_MIGRATION	77	0.4579	1.586102	0.0726	76	0.4963	1.568311	0.0641

**Oncogenic pathway**								
P53_DN.V1_DN	132	0.4987	1.865869	0	133	0.5355	1.770526	0.0101
PTEN_DN.V2_UP	94	0.4781	1.825921	0	94	0.4766	1.647227	0.0261
HOXA9_DN.V1_UP	145	0.5324	1.818311	0.0041	147	0.5021	1.675491	0.039
STK33_NOMO_UP	225	0.491	1.809651	0.0104	227	0.4533	1.637368	0.0456
RPS14_DN.V1_UP	136	0.5811	1.798718	0.0147	142	0.6025	1.857724	0.0081
RELA_DN.V1_UP	86	0.4574	1.758811	0.0021	91	0.4621	1.716071	0.0141
STK33_SKM_UP	211	0.4758	1.739224	0.0128	214	0.4085	1.600525	0.0593
EGFR_UP.V1_UP	149	0.4732	1.737659	0.0104	152	0.5307	1.807555	0.014
STK33_UP	221	0.4736	1.731673	0.0208	225	0.4324	1.631893	0.0504
KRAS.LUNG_UP.V1_UP	74	0.4614	1.71326	0.0067	81	0.4791	1.6239	0.0207
P53_DN.V2_UP	73	0.5017	1.699962	0.0252	79	0.5704	1.919475	0
KRAS.BREAST_UP.V1_UP	68	0.4516	1.602531	0.0434	71	0.4614	1.631583	0.0328

Table S8. Overlapped recurrent mutation genes between Thai HCC and Thai iCCA.

Gene	mut.No.HCC	mut.freq.HCC	mut.No.iCCA	mut.freq.iCCA
TP53	22	0.355	37	0.411
ARID2	9	0.145	6	0.067
ARID1A	9	0.145	17	0.189
CTNNB1	8	0.129	2	0.022
APOB	7	0.113	4	0.044
CSMD3	6	0.097	9	0.100
AXIN1	5	0.081	1	0.011
RYR2	4	0.065	7	0.078
RB1	3	0.048	1	0.011
ASXL1	3	0.048	1	0.011
PTPRD	3	0.048	3	0.033
PKHD1	3	0.048	1	0.011
PIK3CA	3	0.048	4	0.044
PDE4DIP	3	0.048	2	0.022
SMARCA4	3	0.048	2	0.022
PRKDC	3	0.048	6	0.067
SCN5A	3	0.048	4	0.044
NF1	3	0.048	6	0.067
PBRM1	3	0.048	4	0.044
RYR1	3	0.048	4	0.044
KEAP1	3	0.048	2	0.022
MBD1	2	0.032	1	0.011
EPHA5	2	0.032	1	0.011
NCOA3	2	0.032	1	0.011
GRM8	2	0.032	1	0.011
IGF2R	2	0.032	1	0.011
KIT	2	0.032	1	0.011
COL1A1	2	0.032	2	0.022
LRP1B	2	0.032	10	0.111
EPHA7	2	0.032	2	0.022
ERBB4	2	0.032	4	0.044
KMT2C	2	0.032	11	0.122
EML4	2	0.032	1	0.011
SAMD9	2	0.032	1	0.011
EPHB1	2	0.032	1	0.011
PSIP1	2	0.032	5	0.056
UBR5	2	0.032	2	0.022
WHSC1	2	0.032	1	0.011
ACVR2A	2	0.032	3	0.033
SF3B1	2	0.032	1	0.011
DCC	2	0.032	1	0.011
ADAMTS20	2	0.032	7	0.078
NF2	2	0.032	1	0.011
DNMT3A	1	0.016	2	0.022
PTEN	1	0.016	1	0.011
MYH11	1	0.016	3	0.033

JAK1	1	0.016	2	0.022
RICTOR	1	0.016	1	0.011
TSHR	1	0.016	1	0.011
NLRP1	1	0.016	1	0.011
MED12	1	0.016	1	0.011
FBXO11	1	0.016	2	0.022
RNF213	1	0.016	1	0.011
POT1	1	0.016	1	0.011
ACVR1	1	0.016	1	0.011
CHEK2	1	0.016	1	0.011
MYBPC3	1	0.016	1	0.011
BRCA2	1	0.016	3	0.033
TSC2	1	0.016	2	0.022
KMT2A	1	0.016	1	0.011
NSD1	1	0.016	1	0.011
BCORL1	1	0.016	3	0.033
ABL1	1	0.016	2	0.022
EPHA3	1	0.016	1	0.011
NTRK3	1	0.016	1	0.011
AKAP9	1	0.016	3	0.033
FN1	1	0.016	4	0.044
KDM6A	1	0.016	1	0.011
ITGA9	1	0.016	1	0.011
TGFBR2	1	0.016	1	0.011
SYNE1	1	0.016	7	0.078
RNASEL	1	0.016	1	0.011
PTPRT	1	0.016	2	0.022
POLH	1	0.016	2	0.022
CDH5	1	0.016	1	0.011
GUCY1A2	1	0.016	1	0.011
BAI3	1	0.016	4	0.044
LPHN3	1	0.016	1	0.011
COL3A1	1	0.016	3	0.033
AFF3	1	0.016	1	0.011
CACNA1S	1	0.016	1	0.011
ARID1B	1	0.016	1	0.011
HGF	1	0.016	2	0.022
TBX22	1	0.016	1	0.011
IGF1R	1	0.016	1	0.011
EP400	1	0.016	3	0.033
DST	1	0.016	2	0.022
GNAS	1	0.016	3	0.033
ATM	1	0.016	5	0.056
ERBB3	1	0.016	6	0.067
NBN	1	0.016	1	0.011
BAP1	1	0.016	4	0.044
TRRAP	1	0.016	1	0.011
PIK3R1	1	0.016	1	0.011

KRAS	1	0.016	9	0.100
FGFR2	1	0.016	3	0.033
CTNNA1	1	0.016	2	0.022
CDH11	1	0.016	2	0.022
MYLK	1	0.016	1	0.011
MAP3K1	1	0.016	1	0.011

Table S9. Recurrently mutated genes in Thai HCC

Gene	Chr.	POS	REF	ALT	AA Change	Muta tion. Type	Effect	var.frq	snp.alt
TP53	chr17	7578257	C	A	E198*	SNV	STOP_GAINED	1	C>A
TP53	chr17	7577509	C	T	E258K	SNV	NON_SYNONYMOUS_CODING	1	C>T
TP53	chr17	7577535	C	A	R249M	SNV	NON_SYNONYMOUS_CODING	1	C>A
TP53	chr17	7577534	C	A	R249S	SNV	NON_SYNONYMOUS_CODING	7	C>A
TP53	chr17	7578397	TG	T	-178	Indel	FRAME_SHIFT	1	Indel
TP53	chr17	7577114	C	T	C275Y	SNV	NON_SYNONYMOUS_CODING	1	C>T
TP53	chr17	7577132	CT	C	-269	Indel	FRAME_SHIFT	1	Indel
TP53	chr17	7578196	A	T	V218E	SNV	NON_SYNONYMOUS_CODING	1	A>T
TP53	chr17	7577610	T	A		Indel	SPLICE_SITE_ACCEPTOR	1	Indel
TP53	chr17	7577120	C	T	R273H	SNV	NON_SYNONYMOUS_CODING	3	C>T
TP53	chr17	7578515	T	C	K139E	SNV	NON_SYNONYMOUS_CODING	1	T>C
TP53	chr17	7578526	C	G	C135S	SNV	NON_SYNONYMOUS_CODING	1	C>G
TP53	chr17	7577550	C	A	G244V	SNV	NON_SYNONYMOUS_CODING	1	C>A
TP53	chr17	7578271	T	A	H193L	SNV	NON_SYNONYMOUS_CODING	1	T>A
TP53	chr17	7577559	G	T	S241Y	SNV	NON_SYNONYMOUS_CODING	1	G>T
TP53	chr17	7579350	A	C	F113V	SNV	NON_SYNONYMOUS_CODING	1	A>C
TP53	chr17	7578554	A	G	Y126H	SNV	NON_SYNONYMOUS_CODING	1	A>G
TP53	chr17	7579528	C	CCAT	-53Q?	Indel	FRAME_SHIFT	1	Indel

				TG					
TP53	chr17	7577085	C	CT	-284?	Indel	FRAME_SHIFT	1	Indel
TP53	chr17	7578535	T	A	K132M	SNV	NON_SYNONYMOUS_CODING	1	T>A
TP53	chr17	7577501	GGA	G	-260	Indel	FRAME_SHIFT	1	Indel
TP53	chr17	7577550	C	T	G244D	SNV	NON_SYNONYMOUS_CODING	1	C>T
SETD2	chr3	47162374	G	A	S1251L	SNV	NON_SYNONYMOUS_CODING	1	G>A
SETD2	chr3	47125383	T	A	K1963*	SNV	STOP_GAINED	1	T>A
SETD2	chr3	47147605	G	T	T1574N	SNV	NON_SYNONYMOUS_CODING	1	G>T
RYR2	chr1	237880547	G	A	R3456Q	SNV	NON_SYNONYMOUS_CODING	1	G>A
RYR2	chr1	237494269	A	T	R85*	SNV	STOP_GAINED	1	A>T
RYR2	chr1	237777775	C	T	P1781S	SNV	NON_SYNONYMOUS_CODING	1	C>T
RYR2	chr1	237841410	A	G	K2963E	SNV	NON_SYNONYMOUS_CODING	1	A>G
RYR1	chr19	39001168	A	C	K2988T	SNV	NON_SYNONYMOUS_CODING	1	A>C
RYR1	chr19	39034492	C	T	H3997Y	SNV	NON_SYNONYMOUS_CODING	1	C>T
RYR1	chr19	39052033	G	T	R4188L	SNV	NON_SYNONYMOUS_CODING	1	G>T
RPTOR	chr17	78796030	C	T	T307M	SNV	NON_SYNONYMOUS_CODING	1	C>T
RPTOR	chr17	78897465	C	A	P934T	SNV	NON_SYNONYMOUS_CODING	1	C>A
RPTOR	chr17	78921076	A	T	N1064Y	SNV	NON_SYNONYMOUS_CODING	1	A>T
RB1	chr13	48947539	A	G		Indel	SPLICE_SITE_ACCEPTOR	1	Indel
RB1	chr13	4894169	AT	A	-336	Indel	FRAME_SHIFT	1	Indel

		5							
RB1	chr13	48955572	G	C	W563S	SNV	NON_SYNONYMOUS_CODING	1	G>C
PSIP1	chr9	15490113	T	TA	-53?	Indel	FRAME_SHIFT	2	Indel
PSIP1	chr9	15490045	CCTTTT	C	-74	Indel	FRAME_SHIFT	3	Indel
PRKDC	chr8	48765266	G	C	L2324V	SNV	NON_SYNONYMOUS_CODING	1	G>C
PRKDC	chr8	48766699	C	G	V2279L	SNV	NON_SYNONYMOUS_CODING	1	C>G
PRKDC	chr8	48805763	C	T	A1261T	SNV	NON_SYNONYMOUS_CODING	1	C>T
PML	chr15	74315195	C	A	P210Q	SNV	NON_SYNONYMOUS_CODING	1	C>A
PML	chr15	74337311	G	T	G871C	SNV	NON_SYNONYMOUS_CODING	1	G>T
PML	chr15	74315374	C	T	R270C	SNV	NON_SYNONYMOUS_CODING	1	C>T
PKHD1	chr6	51732731	T	A	S2555C	SNV	NON_SYNONYMOUS_CODING	1	T>A
PKHD1	chr6	51771140	T	C		Indel	SPLICE_SITE_ACCEPTOR	1	Indel
PKHD1	chr6	51930833	C	A	R274I	SNV	NON_SYNONYMOUS_CODING	1	C>A
PIK3CA	chr3	178916936	G	A	R108H	SNV	NON_SYNONYMOUS_CODING	1	G>A
PIK3CA	chr3	178952150	TGAAAA	T	-1069	Indel	FRAME_SHIFT	1	Indel
PIK3CA	chr3	178916713	T	G	L34V	SNV	NON_SYNONYMOUS_CODING	1	T>G
PDE4DIP	chr1	144866678	C	A	R1991L	SNV	NON_SYNONYMOUS_CODING	1	C>A
PDE4DIP	chr1	144916577	T	A	E730V	SNV	NON_SYNONYMOUS_CODING	2	T>A

PDE4DIP	chr1	145021150	T	C	D84G	SNV	NON_SYNONYMOUS_CODING	2	T>C
NFE2L2	chr2	178098816	C	G	D77H	SNV	NON_SYNONYMOUS_CODING	1	C>G
NFE2L2	chr2	178098954	C	T	G31R	SNV	NON_SYNONYMOUS_CODING	1	C>T
NFE2L2	chr2	178098953	C	A	G31V	SNV	NON_SYNONYMOUS_CODING	1	C>A
NFE2L2	chr2	178098799	T	A	E82D	SNV	NON_SYNONYMOUS_CODING	1	T>A
NFE2L2	chr2	178098959	T	A	D29V	SNV	NON_SYNONYMOUS_CODING	1	T>A
KIF1B	chr1	10397592	G	A		Indel	SPLICE_SITE_DONOR	1	Indel
KIF1B	chr1	10431274	A	G	T1634A	SNV	NON_SYNONYMOUS_CODING	1	A>G
KIF1B	chr1	10292411	G	A	A9T	SNV	NON_SYNONYMOUS_CODING	1	G>A
KEAP1	chr19	10602866	T	G	N238H	SNV	NON_SYNONYMOUS_CODING	1	T>G
KEAP1	chr19	10602391	T	TA	-396?	Indel	FRAME_SHIFT	1	Indel
KEAP1	chr19	10600446	C	T	R470H	SNV	NON_SYNONYMOUS_CODING	1	C>T
KDR	chr4	55984852	C	A	V93L	SNV	NON_SYNONYMOUS_CODING	1	C>A
KDR	chr4	55979551	CG	C	-299	Indel	FRAME_SHIFT	1	Indel
KDR	chr4	55971152	C	T		Indel	SPLICE_SITE_ACCEPTOR	1	Indel
GRM8	chr7	126883137	C	T	R41Q	SNV	NON_SYNONYMOUS_CODING	1	C>T
GRM8	chr7	126173088	A	G	I783T	SNV	NON_SYNONYMOUS_CODING	3	A>G
CUL2	chr10	35300859	C	T	D694N	SNV	NON_SYNONYMOUS_CODING	1	C>T

CUL2	chr10	35314123	C	A	E560*	SNV	STOP_GAINED	4	C>A
CTNNB1	chr3	41266124	A	G	T41A	SNV	NON_SYNONYMOUS_CODING	3	A>G
CTNNB1	chr3	41266100	T	C	S33P	SNV	NON_SYNONYMOUS_CODING	2	T>C
CTNNB1	chr3	41266113	C	G	S37C	SNV	NON_SYNONYMOUS_CODING	1	C>G
CTNNB1	chr3	41266104	GAATCCATTCTGGTGCCACTACCAC	G	GIHSGATTT34G	Indel	CODON_DELETION	1	Indel
CTNNB1	chr3	41266137	C	T	S45F	SNV	NON_SYNONYMOUS_CODING	2	C>T
CTNNB1	chr3	41266097	G	C	D32H	SNV	NON_SYNONYMOUS_CODING	3	G>C
CTNNB1	chr3	41266097	G	T	D32Y	SNV	NON_SYNONYMOUS_CODING	1	G>T
CTNNB1	chr3	41266098	A	G	D32G	SNV	NON_SYNONYMOUS_CODING	1	A>G
CSMD3	chr8	113504810	G	T	A1729D	SNV	NON_SYNONYMOUS_CODING	1	G>T
CSMD3	chr8	114448950	G	C	T45R	SNV	NON_SYNONYMOUS_CODING	1	G>C
CSMD3	chr8	113702205	G	A	R683C	SNV	NON_SYNONYMOUS_CODING	1	G>A
CSMD3	chr8	113933964	G	A	Q509*	SNV	STOP_GAINED	1	G>A
CSMD3	chr8	113871409	A	G	C574R	SNV	NON_SYNONYMOUS_CODING	2	A>G

CSMD3	chr8	113395877	A	T	F1984I	SNV	NON_SYNONYMOUS_CODING	1	A>T
AXIN1	chr16	396446	C	A	E194*	SNV	STOP_GAINED	1	C>A
AXIN1	chr16	343603	G	A	Q691*	SNV	STOP_GAINED	1	G>A
AXIN1	chr16	347107	C	T	W635*	SNV	STOP_GAINED	1	C>T
AXIN1	chr16	359972	C	T		Indel	SPLICE_SITE_DONOR	1	Indel
AXIN1	chr16	354428	AC	A	-377	Indel	FRAME_SHIFT	1	Indel
ASXL1	chr20	31020700	A	T	M333L	SNV	NON_SYNONYMOUS_CODING	1	A>T
ASXL1	chr20	31024764	C	A	P1417T	SNV	NON_SYNONYMOUS_CODING	1	C>A
ASXL1	chr20	31021502	T	A	S501T	SNV	NON_SYNONYMOUS_CODING	1	T>A
ARID2	chr12	46230748	G	T	D333Y	SNV	NON_SYNONYMOUS_CODING	1	G>T
ARID2	chr12	46245837	A	T	K1311*	SNV	STOP_GAINED	1	A>T
ARID2	chr12	46244453	A	AC	-850?	Indel	FRAME_SHIFT	1	Indel
ARID2	chr12	46233207	C	CT	-476?	Indel	FRAME_SHIFT	1	Indel
ARID2	chr12	46244262	G	T	G786C	SNV	NON_SYNONYMOUS_CODING	1	G>T
ARID2	chr12	46245288	C	T	Q1128*	SNV	STOP_GAINED	1	C>T
ARID2	chr12	46231135	C	CTCAA	-352S?	Indel	FRAME_SHIFT	1	Indel
ARID2	chr12	46246299	CCAAGTGTA	C	-1465	Indel	FRAME_SHIFT	1	Indel
ARID2	chr12	46233219	G	T	E480*	SNV	STOP_GAINED	1	G>T
ARID1A	chr1	27097658	G	T	G1083C	SNV	NON_SYNONYMOUS_CODING	1	G>T
ARID1A	chr1	27107059	G	T	E2224*	SNV	STOP_GAINED	1	G>T
ARID1A	chr1	27094279	A	T		Indel	SPLICE_SITE_ACCEPTOR	1	Indel
ARID1A	chr1	27107023	C	T	Q2212*	SNV	STOP_GAINED	1	C>T
ARID1A	chr1	2709994	C	T	R1276*	SNV	STOP_GAINED	2	C>T

		7							
ARID1A	chr1	27106803	AC	A	-2139	Indel	FRAME_SHIFT	2	Indel
ARID1A	chr1	27105841	C	T	Q1818*	SNV	STOP_GAINED	1	C>T
ARID1A	chr1	27105756	C	CT	-1790?	Indel	FRAME_SHIFT	1	Indel
ARID1A	chr1	27105774	GC	G	-1796	Indel	FRAME_SHIFT	1	Indel
APOB	chr2	21232728	T	A	R2338*	SNV	STOP_GAINED	1	T>A
APOB	chr2	21258549	C	A	S242I	SNV	NON_SYNONYMOUS_CODING	1	C>A
APOB	chr2	21233884	ATG	A	-1952	Indel	FRAME_SHIFT	1	Indel
APOB	chr2	21224995	AGTAAAGTTAGAGGCACTGAC	A	-4427	Indel	FRAME_SHIFT	1	Indel
APOB	chr2	21234944	C	T	R1599H	SNV	NON_SYNONYMOUS_CODING	1	C>T
APOB	chr2	21235067	G	C	T1558S	SNV	NON_SYNONYMOUS_CODING	1	G>C
APOB	chr2	21232800	GAATGTCATTTATTCTTTCAAATGAAAT	G	ISFERINDI2305-	Indel	CODON_DELETION	1	Indel
ACVR2A	chr2	148683685	TA	T	-435	Indel	FRAME_SHIFT	3	Indel
ACVR2A	chr2	148680560	A	G	M366V	SNV	NON_SYNONYMOUS_CODING	1	A>G

Table S10. Recurrently mutated genes in Thai iCCA

Gene	Chr.	POS	REF	ALT	AA Change	Mutation.Type	Effect	var.frq	snp.alt
USP9X	chrX	41075689	G	A	D1957N	SNV	NON_SYNONYMOUS_CODING	1	G>A
USP9X	chrX	41010177	C	T	R544C	SNV	NON_SYNONYMOUS_CODING	1	C>T
USP9X	chrX	41057909	ATTAC	A	-1504	Indel	FRAME_SHIFT	1	Indel
USP9X	chrX	41073850	T	G	L1740R	SNV	NON_SYNONYMOUS_CODING	1	T>G
TP53	chr17	7590693	A	T		Indel	SPLICE_SITE_DONOR	1	Indel
TP53	chr17	7577524	T	C	T253A	SNV	NON_SYNONYMOUS_CODING	1	T>C
TP53	chr17	7577022	G	A	R306*	SNV	STOP_GAINED	2	G>A
TP53	chr17	7577534	C	A	R249S	SNV	NON_SYNONYMOUS_CODING	7	C>A
TP53	chr17	7577566	T	C	N239D	SNV	NON_SYNONYMOUS_CODING	1	T>C
TP53	chr17	7577548	C	T	G245S	SNV	NON_SYNONYMOUS_CODING	1	C>T
TP53	chr17	7578406	C	T	R175H	SNV	NON_SYNONYMOUS_CODING	2	C>T
TP53	chr17	7590692	T	TA		Indel	SPLICE_SITE_DONOR	1	Indel
TP53	chr17	7577118	C	A	V274F	SNV	NON_SYNONYMOUS_CODING	1	C>A
TP53	chr17	7577094	G	A	R282W	SNV	NON_SYNONYMOUS_CODING	2	G>A
TP53	chr17	7577112	C	G	A276P	SNV	NON_SYNONYMOUS_CODING	1	C>G
TP53	chr17	7577120	C	T	R273H	SNV	NON_SYNONYMOUS_CODING	3	C>T
TP53	chr17	7579554	GCATCAAATCATCCA	G	-40	Indel	FRAME_SHIFT	1	Indel
TP53	chr17	7578496	A	T	L145Q	SNV	NON_SYNONYMOUS_CODING	1	A>T
TP53	chr17	7578518	C	G	A138P	SNV	NON_SYNONYMOUS_CODING	1	C>G
TP53	chr17	7578520	A	T	L137Q	SNV	NON_SYNONYMOUS_CODING	1	A>T

TP53	chr17	7577547	C	A	G245V	SNV	NON_SYNONYMOUS _CODING	1	C> A
TP53	chr17	7578431	G	A	Q167*	SNV	STOP_GAINED	1	G> A
TP53	chr17	7577538	C	T	R248Q	SNV	NON_SYNONYMOUS _CODING	3	C> T
TP53	chr17	7577599	C	CA	-227?	Indel	FRAME_SHIFT	1	In del
TP53	chr17	7577115	A	C	C275G	SNV	NON_SYNONYMOUS _CODING	1	A> C
TP53	chr17	7577057	TC	T	-294	Indel	FRAME_SHIFT	1	In del
TP53	chr17	7578176	C	T		Indel	SPLICE_SITE_DONOR	1	In del
TP53	chr17	7578413	C	A	V173L	SNV	NON_SYNONYMOUS _CODING	1	C> A
TP53	chr17	7579359	G	GGA AAC CGT	- 109TV?	Indel	FRAME_SHIFT	1	In del
TP53	chr17	7576870	C	A	E326*	SNV	STOP_GAINED	1	C> A
TP53	chr17	7578212	G	A	R213*	SNV	STOP_GAINED	1	G> A
TP53	chr17	7578410	T	A	R174W	SNV	NON_SYNONYMOUS _CODING	1	T> A
TP53	chr17	7577124	C	T	V272M	SNV	NON_SYNONYMOUS _CODING	1	C> T
TP53	chr17	7578263	G	A	R196*	SNV	STOP_GAINED	1	G> A
TP53	chr17	7576896	TG	T	-317	Indel	FRAME_SHIFT	1	In del
TP53	chr17	7579343	T	C	H115R	SNV	NON_SYNONYMOUS _CODING	1	T> C
TP53	chr17	7579373	C	A	G105V	SNV	NON_SYNONYMOUS _CODING	1	C> A
TP53	chr17	7579854	GA	G	-20	Indel	FRAME_SHIFT	1	In del
TP53	chr17	7574018	G	A	R337C	SNV	NON_SYNONYMOUS _CODING	1	G> A
TP53	chr17	7578262	C	G	R196P	SNV	NON_SYNONYMOUS _CODING	1	C> G
TP53	chr17	7577099	C	G	R280T	SNV	NON_SYNONYMOUS _CODING	1	C> G
TGFB R1	chr9	101904946	G	A	G316S	SNV	NON_SYNONYMOUS _CODING	1	G> A
TGFB R1	chr9	101891287	C	T	P83L	SNV	NON_SYNONYMOUS _CODING	1	C> T
TGFB R1	chr9	101891136	GC GT TA C	G	ALQ33 E	Indel	CODON_CHANGE_PL US_CODON_DELETIO N	1	In del

SYNE1	chr6	152651967	A	C	L4618R	SNV	NON_SYNONYMOUS_CODING	1	A>C
SYNE1	chr6	152651058	T	C	Q4921R	SNV	NON_SYNONYMOUS_CODING	1	T>C
SYNE1	chr6	152527476	G	A	R7616W	SNV	NON_SYNONYMOUS_CODING	1	G>A
SYNE1	chr6	152485406	C	T	M7894I	SNV	NON_SYNONYMOUS_CODING	1	C>T
SYNE1	chr6	152555825	C	T	R6836H	SNV	NON_SYNONYMOUS_CODING	1	C>T
SYNE1	chr6	152599315	T	G	E6161A	SNV	NON_SYNONYMOUS_CODING	1	T>G
SYNE1	chr6	152765644	C	A	E1247*	SNV	STOP_GAINED	1	C>A
STK11	chr19	1207033	AAGCG	A	-41	Indel	FRAME_SHIFT	1	Indel
STK11	chr19	1221995	C	T	R304W	SNV	NON_SYNONYMOUS_CODING	1	C>T
STK11	chr19	1220487	G	A	D194N	SNV	NON_SYNONYMOUS_CODING	1	G>A
STK11	chr19	1207054	A	T	K48*	SNV	STOP_GAINED	1	A>T
STAG1	chr3	136191310	C	G	D384H	SNV	NON_SYNONYMOUS_CODING	1	C>G
STAG1	chr3	136221548	T	A	Q250H	SNV	NON_SYNONYMOUS_CODING	1	T>A
STAG1	chr3	136342005	G	A	R39C	SNV	NON_SYNONYMOUS_CODING	1	G>A
SRC	chr20	36031729	G	T	E526*	SNV	STOP_GAINED	1	G>T
SRC	chr20	36014513	G	A	E96K	SNV	NON_SYNONYMOUS_CODING	1	G>A
SRC	chr20	36031750	G	A	E533K	SNV	NON_SYNONYMOUS_CODING	1	G>A
SMAD4	chr18	48593406	G	A	G386D	SNV	NON_SYNONYMOUS_CODING	1	G>A
SMAD4	chr18	48591880	TTACTGTTGA	T	VTVD348V	Indel	CODON_DELETION	1	Indel
SMAD4	chr18	48591898	T	G	V354G	SNV	NON_SYNONYMOUS_CODING	1	T>G
SMAD4	chr18	48591918	C	T	R361C	SNV	NON_SYNONYMOUS_CODING	4	C>T
SMAD4	chr18	48604764	T	TA	-529?	Indel	FRAME_SHIFT	1	Indel
SMAD4	chr18	48591910	GAGGAG	G	GGD358G	Indel	CODON_CHANGE_PLUS_CODON_DELETION	1	Indel

			A						
SMA D4	chr18	48604750	G	T	W524C	SNV	NON_SYNONYMOUS _CODING	1	G> T
SMA D4	chr18	48584497	C	T	Q224*	SNV	STOP_GAINED	1	C> T
SMA D4	chr18	48575219	C	G	S138*	SNV	STOP_GAINED	1	C> G
SMA D4	chr18	48593531	A	T	K428*	SNV	STOP_GAINED	1	A> T
SMA D4	chr18	48584593	C	T	Q256*	SNV	STOP_GAINED	1	C> T
SMA D4	chr18	48573528	A	T	R38*	SNV	STOP_GAINED	1	A> T
SMA D4	chr18	48584513	TG	T	-229	Indel	FRAME_SHIFT	1	In del
SCN5 A	chr3	38608045	C	T	R1232Q	SNV	NON_SYNONYMOUS _CODING	1	C> T
SCN5 A	chr3	38640457	G	A	R659W	SNV	NON_SYNONYMOUS _CODING	1	G> A
SCN5 A	chr3	38603943	C	T	R1309H	SNV	NON_SYNONYMOUS _CODING	1	C> T
SCN5 A	chr3	38647567	C	T	V405M	SNV	NON_SYNONYMOUS _CODING	1	C> T
RYR2	chr1	237969498	T	C	F4744S	SNV	NON_SYNONYMOUS _CODING	1	T> C
RYR2	chr1	237804248	G	A	M2387I	SNV	NON_SYNONYMOUS _CODING	1	G> A
RYR2	chr1	237850754	GC	G	-3004	Indel	FRAME_SHIFT	1	In del
RYR2	chr1	237868562	C	T	P3165S	SNV	NON_SYNONYMOUS _CODING	3	C> T
RYR2	chr1	237670041	G	C	R880T	SNV	NON_SYNONYMOUS _CODING	1	G> C
RYR2	chr1	237580409	G	C	E276D	SNV	NON_SYNONYMOUS _CODING	1	G> C
RYR2	chr1	237755097	G	T	E1405*	SNV	STOP_GAINED	1	G> T
RYR1	chr19	38934854	G	T	V164F	SNV	NON_SYNONYMOUS _CODING	1	G> T
RYR1	chr19	38979850	CA	C	-1861	Indel	FRAME_SHIFT	1	In del
RYR1	chr19	38949797	C	T	R727C	SNV	NON_SYNONYMOUS _CODING	1	C> T
RYR1	chr19	38998374	G	A	D2947N	SNV	NON_SYNONYMOUS _CODING	1	G> A
RUN X1T1	chr8	92998513	C	T	R384Q	SNV	NON_SYNONYMOUS _CODING	1	C> T
RUN X1T1	chr8	93029565	G	A	P50S	SNV	NON_SYNONYMOUS _CODING	1	G> A
RUN	chr8	93004062	G	A	R277*	SNV	STOP_GAINED	1	G>

X1T1									A
RUN X1T1	chr8	92982983	G	A	T492I	SNV	NON_SYNONYMOUS _CODING	1	G> A
RNF4 3	chr17	56436140	G	A	Q333*	SNV	STOP_GAINED	1	G> A
RNF4 3	chr17	56492739	C	T	G67D	SNV	NON_SYNONYMOUS _CODING	1	C> T
RNF4 3	chr17	56440741	C	T	W159*	SNV	STOP_GAINED	1	C> T
RNF4 3	chr17	56438222	AC CC CG	A	-256	Indel	FRAME_SHIFT	1	In del
RNF4 3	chr17	56437534	G	T	P310T	SNV	NON_SYNONYMOUS _CODING	1	G> T
PTPR D	chr9	8636761	C	G	D50H	SNV	NON_SYNONYMOUS _CODING	1	C> G
PTPR D	chr9	8633407	G	A	R88W	SNV	NON_SYNONYMOUS _CODING	1	G> A
PTPR D	chr9	8460493	C	T	E1265K	SNV	NON_SYNONYMOUS _CODING	1	C> T
PSIP1	chr9	15490045	CC TTT T	C	-74	Indel	FRAME_SHIFT	3	In del
PSIP1	chr9	15490113	TA	T	-53	Indel	FRAME_SHIFT	1	In del
PSIP1	chr9	15469290	T	A	K360*	SNV	STOP_GAINED	1	T> A
PSIP1	chr9	15506572	C	A	G46*	SNV	STOP_GAINED	1	C> A
PSIP1	chr9	15472749	C	A		Indel	SPLICE_SITE_ACCEP TOR	1	In del
PRK DC	chr8	48830924	C	T	W813*	SNV	STOP_GAINED	1	C> T
PRK DC	chr8	48749959	C	T	W2524*	SNV	STOP_GAINED	1	C> T
PRK DC	chr8	48713478	C	T	G3330D	SNV	NON_SYNONYMOUS _CODING	1	C> T
PRK DC	chr8	48817428	C	G		Indel	SPLICE_SITE_DONOR	1	In del
PRK DC	chr8	48848395	C	A	Q448H	SNV	NON_SYNONYMOUS _CODING	1	C> A
PRK DC	chr8	48811031	G	A	R1155*	SNV	STOP_GAINED	1	G> A
PIK3 CA	chr3	178936091	G	A	E545K	SNV	NON_SYNONYMOUS _CODING	1	G> A
PIK3 CA	chr3	178921553	T	A	N345K	SNV	NON_SYNONYMOUS _CODING	2	T> A
PIK3 CA	chr3	178943780	T	G	I816S	SNV	NON_SYNONYMOUS _CODING	1	T> G
PIK3 CA	chr3	178952085	A	G	H1047R	SNV	NON_SYNONYMOUS _CODING	1	A> G

PBRM1	chr3	52584801	CTACCATA	C	-1545	Indel	FRAME_SHIFT	1	Indel
PBRM1	chr3	52682399	A	AT	-258?	Indel	FRAME_SHIFT	2	Indel
PBRM1	chr3	52643328	C	A		Indel	SPLICE_SITE_DONOR	1	Indel
PBRM1	chr3	52682407	CGAG	C	L255-	Indel	CODON_DELETION	1	Indel
NF1	chr17	29684326	C	T	R2637*	SNV	STOP_GAINED	1	C>T
NF1	chr17	29554310	G	C		Indel	SPLICE_SITE_DONOR	1	Indel
NF1	chr17	29486094	G	GA	-91?	Indel	FRAME_SHIFT	1	Indel
NF1	chr17	29652920	G	T	E1640*	SNV	STOP_GAINED	1	G>T
NF1	chr17	29657433	C	CT	-1910?	Indel	FRAME_SHIFT	1	Indel
NF1	chr17	29663350	G	A		Indel	SPLICE_SITE_ACCEPTOR	1	Indel
MYH11	chr16	15815482	C	T	E1466K	SNV	NON_SYNONYMOUS_CODING	1	C>T
MYH11	chr16	15932094	G	A	Q6*	SNV	STOP_GAINED	1	G>A
MYH11	chr16	15844174	C	T	G634S	SNV	NON_SYNONYMOUS_CODING	1	C>T
MAP2K4	chr17	12028657	G	A	R298H	SNV	NON_SYNONYMOUS_CODING	1	G>A
MAP2K4	chr17	12028638	C	T	R292*	SNV	STOP_GAINED	1	C>T
MAP2K4	chr17	12028660	C	T	S299F	SNV	NON_SYNONYMOUS_CODING	1	C>T
MAP2K4	chr17	12016634	C	G	S268C	SNV	NON_SYNONYMOUS_CODING	1	C>G
MAP2K4	chr17	11984802	CAACAAAATG	C	NKM128-	Indel	CODON_DELETION	1	Indel
LRP1B	chr2	141143498	T	A	N3499Y	SNV	NON_SYNONYMOUS_CODING	1	T>A
LRP1B	chr2	141122280	TCA	T	-3693	Indel	FRAME_SHIFT	1	Indel
LRP1B	chr2	141214083	T	C	T3302A	SNV	NON_SYNONYMOUS_CODING	1	T>C
LRP1B	chr2	140995804	G	T	P4493T	SNV	NON_SYNONYMOUS_CODING	1	G>T
LRP1B	chr2	141253256	T	C	E2971G	SNV	NON_SYNONYMOUS_CODING	1	T>C
LRP1B	chr2	141264371	G	A	Q2839*	SNV	STOP_GAINED	1	G>A

LRP1B	chr2	141643848	G	C	L1275V	SNV	NON_SYNONYMOUS_CODING	1	G>C
LRP1B	chr2	141243094	C	A		Indel	SPLICE_SITE_ACCEPTOR	1	Indel
LRP1B	chr2	141473551	G	C	P2005R	SNV	NON_SYNONYMOUS_CODING	1	G>C
LRP1B	chr2	141299411	G	T	L2442I	SNV	NON_SYNONYMOUS_CODING	1	G>T
KRAS	chr12	25398284	C	T	G12D	SNV	NON_SYNONYMOUS_CODING	6	C>T
KRAS	chr12	25398284	C	A	G12V	SNV	NON_SYNONYMOUS_CODING	3	C>A
KRAS	chr12	25398285	C	T	G12S	SNV	NON_SYNONYMOUS_CODING	2	C>T
KRAS	chr12	25378562	C	T	A146T	SNV	NON_SYNONYMOUS_CODING	1	C>T
KRAS	chr12	25380275	T	G	Q61H	SNV	NON_SYNONYMOUS_CODING	1	T>G
KRAS	chr12	25398282	C	A	G13C	SNV	NON_SYNONYMOUS_CODING	1	C>A
KRAS	chr12	25398285	C	A	G12C	SNV	NON_SYNONYMOUS_CODING	2	C>A
KRAS	chr12	25398262	C	A	L19F	SNV	NON_SYNONYMOUS_CODING	1	C>A
KRAS	chr12	25398281	C	T	G13D	SNV	NON_SYNONYMOUS_CODING	1	C>T
KMT2C	chr7	151874120	GAGAACCA	G	-2804	Indel	FRAME_SHIFT	1	Indel
KMT2C	chr7	152012245	G	A	R190*	SNV	STOP_GAINED	1	G>A
KMT2C	chr7	151949801	C	G		Indel	SPLICE_SITE_ACCEPTOR	1	Indel
KMT2C	chr7	151856055	G	A	Q3855*	SNV	STOP_GAINED	1	G>A
KMT2C	chr7	151859201	C	A		Indel	SPLICE_SITE_DONOR	1	Indel
KMT2C	chr7	151851531	C	T		Indel	SPLICE_SITE_ACCEPTOR	2	Indel
KMT2C	chr7	151947040	T	C		Indel	SPLICE_SITE_ACCEPTOR	1	Indel
KMT2C	chr7	151879136	G	A	Q1937*	SNV	STOP_GAINED	1	G>A
KMT2C	chr7	151845261	C	T	R4641Q	SNV	NON_SYNONYMOUS_CODING	1	C>T
KMT2C	chr7	151884352	A	C	F1668C	SNV	NON_SYNONYMOUS_CODING	1	A>C
KMT2C	chr7	151949138	C	A	E503*	SNV	STOP_GAINED	1	C>A

KAT6B	chr10	76739051	G	C	E729Q	SNV	NON_SYNONYMOUS_CODING	1	G>C
KAT6B	chr10	76788859	C	T	A1426V	SNV	NON_SYNONYMOUS_CODING	1	C>T
KAT6B	chr10	76789762	A	T	N1727I	SNV	NON_SYNONYMOUS_CODING	1	A>T
KAT6B	chr10	76781905	G	GGAA	-1097E	Indel	CODON_INSERTION	1	Indel
KAT6B	chr10	76781905	GGAA	G	E1097-	Indel	CODON_DELETION	1	Indel
GNAS	chr20	57484421	G	A	R844H	SNV	NON_SYNONYMOUS_CODING	6	G>A
GNAS	chr20	57484420	C	T	R844C	SNV	NON_SYNONYMOUS_CODING	9	C>T
GNAS	chr20	57428925	C	T	P202L	SNV	NON_SYNONYMOUS_CODING	1	C>T
FN1	chr2	216271877	G	C	Q896E	SNV	NON_SYNONYMOUS_CODING	1	G>C
FN1	chr2	216284088	T	C	T566A	SNV	NON_SYNONYMOUS_CODING	1	T>C
FN1	chr2	216286852	C	G	R503P	SNV	NON_SYNONYMOUS_CODING	1	C>G
FN1	chr2	216274397	C	T	E730K	SNV	NON_SYNONYMOUS_CODING	1	C>T
FGFR2	chr10	123247514	C	A	K660N	SNV	NON_SYNONYMOUS_CODING	1	C>A
FGFR2	chr10	123247516	T	C	K660E	SNV	NON_SYNONYMOUS_CODING	1	T>C
FGFR2	chr10	123258034	A	C	N550K	SNV	NON_SYNONYMOUS_CODING	1	A>C
FANCI	chr15	89816675	C	A	T317K	SNV	NON_SYNONYMOUS_CODING	4	C>A
FANCI	chr15	89816698	C	A	Q325K	SNV	NON_SYNONYMOUS_CODING	4	C>A
ERBB4	chr2	212566818	C	T	A455T	SNV	NON_SYNONYMOUS_CODING	1	C>T
ERBB4	chr2	212251768	AGCACCCTGT	A	AQGA1094A	Indel	CODON_CHANGE_PLUS_CODON_DELETION	1	Indel
ERBB4	chr2	212426794	C	A	S774I	SNV	NON_SYNONYMOUS_CODING	1	C>A
ERBB4	chr2	212570064	G	A	R393W	SNV	NON_SYNONYMOUS_CODING	1	G>A
ERBB3	chr12	56481696	T	C	F244S	SNV	NON_SYNONYMOUS_CODING	1	T>C
ERBB3	chr12	56481922	G	A	G284R	SNV	NON_SYNONYMOUS_CODING	3	G>A

ERB B3	chr12	56478854	G	A	V104M	SNV	NON_SYNONYMOUS _CODING	1	G> A
ERB B3	chr12	56482537	G	A	E332K	SNV	NON_SYNONYMOUS _CODING	1	G> A
ERB B3	chr12	56482341	G	T	D297Y	SNV	NON_SYNONYMOUS _CODING	1	G> T
ERB B3	chr12	56488249	C	T	P590S	SNV	NON_SYNONYMOUS _CODING	1	C> T
ERB B2	chr17	37868208	C	T	S310F	SNV	NON_SYNONYMOUS _CODING	6	C> T
ERB B2	chr17	37880220	T	C	L755S	SNV	NON_SYNONYMOUS _CODING	1	T> C
ERB B2	chr17	37868208	C	A	S310Y	SNV	NON_SYNONYMOUS _CODING	3	C> A
EPH A6	chr3	97124093	A	G	D569G	SNV	NON_SYNONYMOUS _CODING	1	A> G
EPH A6	chr3	97202892	T	C	V730A	SNV	NON_SYNONYMOUS _CODING	1	T> C
EPH A6	chr3	97251304	G	A	R768K	SNV	NON_SYNONYMOUS _CODING	1	G> A
EP40 0	chr12	132530375	G	A	R2413Q	SNV	NON_SYNONYMOUS _CODING	1	G> A
EP40 0	chr12	132522288	C	T	R2041*	SNV	STOP_GAINED	1	C> T
EP40 0	chr12	132512637	C	A	P1765T	SNV	NON_SYNONYMOUS _CODING	1	C> A
CSM D3	chr8	114326810	A	T	F131I	SNV	NON_SYNONYMOUS _CODING	1	A> T
CSM D3	chr8	113304844	C	T	G2904R	SNV	NON_SYNONYMOUS _CODING	1	C> T
CSM D3	chr8	113266510	C	G	G3361A	SNV	NON_SYNONYMOUS _CODING	1	C> G
CSM D3	chr8	113348949	AG	A	-2317	Indel	FRAME_SHIFT	1	In del
CSM D3	chr8	113702263	AC TTT	A	-662	Indel	FRAME_SHIFT	1	In del
CSM D3	chr8	113871376	T	G	K585Q	SNV	NON_SYNONYMOUS _CODING	1	T> G
CSM D3	chr8	113871409	A	G	C574R	SNV	NON_SYNONYMOUS _CODING	2	A> G
CSM D3	chr8	113308152	G	A	Q2842*	SNV	STOP_GAINED	1	G> A
CSM D3	chr8	113267612	C	T	E3303K	SNV	NON_SYNONYMOUS _CODING	1	C> T
COL3 A1	chr2	189861915	C	T	R596*	SNV	STOP_GAINED	1	C> T
COL3 A1	chr2	189854843	C	T	R238*	SNV	STOP_GAINED	1	C> T
COL3 A1	chr2	189870103	G	A	G987S	SNV	NON_SYNONYMOUS _CODING	1	G> A

CDK N2A	chr9	21971203	AT GA C	A	-106	Indel	FRAME_SHIFT	1	In del
CDK N2A	chr9	21971111	G	A	A138V	SNV	NON_SYNONYMOUS _CODING	2	G> A
CDK N2A	chr9	21971099	G	A	P142L	SNV	NON_SYNONYMOUS _CODING	1	G> A
BRC A2	chr13	32905055	G	T		Indel	SPLICE_SITE_ACCEP TOR	1	In del
BRC A2	chr13	32929036	T	G	F2349C	SNV	NON_SYNONYMOUS _CODING	1	T> G
BRC A2	chr13	32912621	A	C	N1377H	SNV	NON_SYNONYMOUS _CODING	1	A> C
BCO RL1	chrX	129159070	G	A	R1265H	SNV	NON_SYNONYMOUS _CODING	1	G> A
BCO RL1	chrX	129155104	C	T	R1196*	SNV	STOP_GAINED	1	C> T
BCO RL1	chrX	129149383	G	T	E879*	SNV	STOP_GAINED	1	G> T
BAP1	chr3	52441413	A	T		Indel	SPLICE_SITE_DONOR	1	In del
BAP1	chr3	52441235	G	A	R179W	SNV	NON_SYNONYMOUS _CODING	1	G> A
BAP1	chr3	52440906	C	A	E200*	SNV	STOP_GAINED	1	C> A
BAP1	chr3	52439796	C	CAA	-305?	Indel	FRAME_SHIFT	1	In del
BAI3	chr6	70037710	A	C		Indel	SPLICE_SITE_ACCEP TOR	1	In del
BAI3	chr6	70049284	C	T	A1116V	SNV	NON_SYNONYMOUS _CODING	1	C> T
BAI3	chr6	70071276	G	C	E1371Q	SNV	NON_SYNONYMOUS _CODING	1	G> C
BAI3	chr6	69348853	G	GA	-96?	Indel	FRAME_SHIFT	1	In del
ATR X	chrX	76939697	C	A	E351*	SNV	STOP_GAINED	1	C> A
ATR X	chrX	76849277	T	C	D2000G	SNV	NON_SYNONYMOUS _CODING	1	T> C
ATR X	chrX	76972716	T	G	S9R	SNV	NON_SYNONYMOUS _CODING	1	T> G
ATM	chr11	108160464	G	A	G1458R	SNV	NON_SYNONYMOUS _CODING	1	G> A
ATM	chr11	108198387	A	T	K2331*	SNV	STOP_GAINED	1	A> T
ATM	chr11	108186766	T	C	W2042 R	SNV	NON_SYNONYMOUS _CODING	1	T> C
ATM	chr11	108121684	G	T	E498*	SNV	STOP_GAINED	1	G> T
ATM	chr11	108216476	C	CA	-2809?	Indel	FRAME_SHIFT	1	In del
ARID	chr12	46244611	CT	C	-902	Indel	FRAME_SHIFT	1	In

2									del
ARID 2	chr12	46245865	C	A	S1320*	SNV	STOP_GAINED	1	C> A
ARID 2	chr12	46231491	G	A		Indel	SPLICE_SITE_DONOR	1	In del
ARID 2	chr12	46231104	CT	C	-342	Indel	FRAME_SHIFT	1	In del
ARID 2	chr12	46215255	T	TA	-231?	Indel	FRAME_SHIFT	1	In del
ARID 2	chr12	46244803	CT AA CA	C	-966	Indel	FRAME_SHIFT	1	In del
ARID 1A	chr1	27105579	T	TC	-1731?	Indel	FRAME_SHIFT	1	In del
ARID 1A	chr1	27101300	C	T	R1528*	SNV	STOP_GAINED	1	C> T
ARID 1A	chr1	27105930	T	TG	-1848?	Indel	FRAME_SHIFT	2	In del
ARID 1A	chr1	27105736	G	T	E1783*	SNV	STOP_GAINED	1	G> T
ARID 1A	chr1	27106335	CT GT G	C	-1983	Indel	FRAME_SHIFT	1	In del
ARID 1A	chr1	27106549	G	T	E2054*	SNV	STOP_GAINED	1	G> T
ARID 1A	chr1	27057835	C	T	Q515*	SNV	STOP_GAINED	2	C> T
ARID 1A	chr1	27057967	CC T	C	-559	Indel	FRAME_SHIFT	1	In del
ARID 1A	chr1	27099947	C	T	R1276*	SNV	STOP_GAINED	2	C> T
ARID 1A	chr1	27106803	AC	A	-2139	Indel	FRAME_SHIFT	2	In del
ARID 1A	chr1	27106005	AC	A	-1873	Indel	FRAME_SHIFT	1	In del
ARID 1A	chr1	27100284	CT CC TA TA GA CA TG AT	C	-1335	Indel	FRAME_SHIFT	1	In del
ARID 1A	chr1	27056282	CC CG CA GC G	C	-427	Indel	FRAME_SHIFT	1	In del
ARID 1A	chr1	27056292	T	C	Y430H	SNV	NON_SYNONYMOUS _CODING	1	T> C
ARID 1A	chr1	27099878	AA CG GC GG GA	A	-1253	Indel	FRAME_SHIFT	1	In del

			TG GG						
ARID 1A	chr1	27099023	C	A	P1147T	SNV	NON_SYNONYMOUS _CODING	1	C> A
ARID 1A	chr1	27056211	C	T	Q403*	SNV	STOP_GAINED	1	C> T
APO B	chr2	21233296	C	CT	-2148?	Indel	FRAME_SHIFT	1	In del
APO B	chr2	21230961	CT	C	-2926	Indel	FRAME_SHIFT	1	In del
APO B	chr2	21238133	C	T		Indel	SPLICE_SITE_ACCEP TOR	1	In del
APO B	chr2	21246458	A	C	I848R	SNV	NON_SYNONYMOUS _CODING	1	A> C
APC	chr5	112175951	G	GA	-1554?	Indel	FRAME_SHIFT	1	In del
APC	chr5	112175925	CA	C	-1545	Indel	FRAME_SHIFT	1	In del
APC	chr5	112177529	T	A	L2080 M	SNV	NON_SYNONYMOUS _CODING	1	T> A
APC	chr5	112154822	A	T	K365*	SNV	STOP_GAINED	1	A> T
APC	chr5	112175108	A	T	R1273*	SNV	STOP_GAINED	1	A> T
APC	chr5	112175879	G	T	E1530*	SNV	STOP_GAINED	1	G> T
APC	chr5	112175675	A	AAG	-1462?	Indel	FRAME_SHIFT	1	In del
APC	chr5	112177217	G	A	D1976N	SNV	NON_SYNONYMOUS _CODING	1	G> A
APC	chr5	112164670	G	T		Indel	SPLICE_SITE_DONOR	1	In del
APC	chr5	112175897	G	GA	-1536?	Indel	FRAME_SHIFT	1	In del
AKA P9	chr7	91714999	A	C	E3012A	SNV	NON_SYNONYMOUS _CODING	1	A> C
AKA P9	chr7	91631813	A	G	Y873C	SNV	NON_SYNONYMOUS _CODING	1	A> G
AKA P9	chr7	91691747	T	A	L1987*	SNV	STOP_GAINED	1	T> A
ADA MTS2 0	chr12	43944731	A	C	V145G	SNV	NON_SYNONYMOUS _CODING	1	A> C
ADA MTS2 0	chr12	43833726	G	C	R813G	SNV	NON_SYNONYMOUS _CODING	1	G> C
ADA MTS2 0	chr12	43858516	G	T	L463I	SNV	NON_SYNONYMOUS _CODING	1	G> T
ADA MTS2 0	chr12	43828120	G	A	S883F	SNV	NON_SYNONYMOUS _CODING	1	G> A

ADA MTS2 0	chr12	43777734	C	T	C1500Y	SNV	NON_SYNONYMOUS _CODING	1	C> T
ADA MTS2 0	chr12	43792997	C	A	V1442L	SNV	NON_SYNONYMOUS _CODING	1	C> A
ADA MTS2 0	chr12	43840436	C	T	C720Y	SNV	NON_SYNONYMOUS _CODING	1	C> T
ACV R2A	chr2	148683685	TA	T	-435	Indel	FRAME_SHIFT	3	In del
ACV R2A	chr2	148657040	G	GA	-93?	Indel	FRAME_SHIFT	1	In del
ACV R2A	chr2	148683685	T	TA	-435?	Indel	FRAME_SHIFT	2	In del
ACV R1B	chr12	52379131	C	T	R420*	SNV	STOP_GAINED	1	C> T
ACV R1B	chr12	52379123	G	A	G417E	SNV	NON_SYNONYMOUS _CODING	1	G> A
ACV R1B	chr12	52369210	CT GA GC TC GG AG	C	-85	Indel	FRAME_SHIFT	1	In del
ACV R1B	chr12	52377825	A	G	H326R	SNV	NON_SYNONYMOUS _CODING	1	A> G

Table S11. Information of TP53 mutations mapping.

Sample ID	AA change	Type	Chr	Start Pos	End Pos	Ref	Var
**Thai HCC**							
LCS_501A	E198*	Nonsense	chr17	7578257	7578257	C	A
LCS_505A	E258K	Missense	chr17	7577509	7577509	C	T
LCS_528A	R249M	Missense	chr17	7577535	7577535	C	A
LCS_512A	R249S	Missense	chr17	7577534	7577534	C	A
LCS_508A	R249S	Missense	chr17	7577534	7577534	C	A
LCS_513A	-178	FS	chr17	7578397	7578397	TG	T
LCS_541A	C275Y	Missense	chr17	7577114	7577114	C	T
LCS_542A	-269	FS	chr17	7577132	7577132	CT	C
LCS_540A	V218E	Missense	chr17	7578196	7578196	A	T
LCS_538A		Splice	chr17	7577610	7577610	T	A
LCS_562A	R273H	Missense	chr17	7577120	7577120	C	T
LCS_524A	K139E	Missense	chr17	7578515	7578515	T	C
LCS_533A	C135S	Missense	chr17	7578526	7578526	C	G
LCS_568A	G244V	Missense	chr17	7577550	7577550	C	A
LCS_584A	H193L	Missense	chr17	7578271	7578271	T	A
LCS_530A	R249S	Missense	chr17	7577534	7577534	C	A
LCS_511A	S241Y	Missense	chr17	7577559	7577559	G	T
LCS_510A	R249S	Missense	chr17	7577534	7577534	C	A
LCS_559A	F113V	Missense	chr17	7579350	7579350	A	C
LCS_566A	Y126H	Missense	chr17	7578554	7578554	A	G
LCS_536A	-53Q?	FS	chr17	7579528	7579528	C	CCATTG
LCS_532A	-284?	FS	chr17	7577085	7577085	C	CT
LCS_563A	K132M	Missense	chr17	7578535	7578535	T	A
LCS_509A	-260	FS	chr17	7577501	7577501	GGA	G
LCS_507A	G244D	Missense	chr17	7577550	7577550	C	T
LCS_519A	R249S	Missense	chr17	7577534	7577534	C	A
LCS_550A	R249S	Missense	chr17	7577534	7577534	C	A
**Thai iCCA**							
LCS_612A		Splice	chr17	7590693	7590693	A	T
LCS_578A	T253A	Missense	chr17	7577524	7577524	T	C
LCS_618A	R306*	Nonsense	chr17	7577022	7577022	G	A
LCS_573A	N239D	Missense	chr17	7577566	7577566	T	C
LCS_681A	G245S	Missense	chr17	7577548	7577548	C	T
LCS_595A	R175H	Missense	chr17	7578406	7578406	C	T
LCS_626A		Splice	chr17	7590692	7590692	T	TA
LCS_624A	V274F	Missense	chr17	7577118	7577118	C	A
LCS_577A	A276P	Missense	chr17	7577112	7577112	C	G
LCS_680A	-40	FS	chr17	7579554	7579554	GCATCAAATCATCCA	G
LCS_649A	L145Q	Missense	chr17	7578496	7578496	A	T
LCS_623A	A138P	Missense	chr17	7578518	7578518	C	G

LCS_580A	L137Q	Missense	chr17	7578520	7578520	A	T
LCS_598A	G245V	Missense	chr17	7577547	7577547	C	A
LCS_574A	Q167*	Nonsense	chr17	7578431	7578431	G	A
LCS_685A	-227?	FS	chr17	7577599	7577599	C	CA
LCS_627A	C275G	Missense	chr17	7577115	7577115	A	C
LCS_669A	-294	FS	chr17	7577057	7577057	TC	T
LCS_645A		Splice	chr17	7578176	7578176	C	T
LCS_644A	R273H	Missense	chr17	7577120	7577120	C	T
LCS_687A	R273H	Missense	chr17	7577120	7577120	C	T
LCS_688A	V173L	Missense	chr17	7578413	7578413	C	A
LCS_572A	R249S	Missense	chr17	7577534	7577534	C	A
LCS_571A	-109TV?	FS	chr17	7579359	7579359	G	GGAAA CCGT
LCS_630A	E326*	Nonsense	chr17	7576870	7576870	C	A
LCS_640A	R282W	Missense	chr17	7577094	7577094	G	A
LCS_652A	R213*	Nonsense	chr17	7578212	7578212	G	A
LCS_658A	R248Q	Missense	chr17	7577538	7577538	C	T
LCS_600A	R174W	Missense	chr17	7578410	7578410	T	A
LCS_616A	V272M	Missense	chr17	7577124	7577124	C	T
LCS_646A	R196*	Nonsense	chr17	7578263	7578263	G	A
LCS_631A	R306*	Nonsense	chr17	7577022	7577022	G	A
LCS_610A	-317	FS	chr17	7576896	7576896	TG	T
LCS_583A	R248Q	Missense	chr17	7577538	7577538	C	T
LCS_583A	H115R	Missense	chr17	7579343	7579343	T	C
LCS_670A	G105V	Missense	chr17	7579373	7579373	C	A
LCS_604A	-20	FS	chr17	7579854	7579854	GA	G
LCS_620A	R337C	Missense	chr17	7574018	7574018	G	A
LCS_679A	R196P	Missense	chr17	7578262	7578262	C	G
LCS_642A	R175H	Missense	chr17	7578406	7578406	C	T
LCS_674A	R280T	Missense	chr17	7577099	7577099	C	G
**TCGA HCC**							
TCGA-UB-A7MC-01	R248Q	Missense	chr17	7577538	7577538	C	T
TCGA-XR-A8TG-01	R248Q	Missense	chr17	7577538	7577538	C	T
TCGA-WQ-AB4B-01	R248Q	Missense	chr17	7577538	7577538	C	T
TCGA-ED-A459-01	Y220C	Missense	chr17	7578190	7578190	T	C
TCGA-DD-AADD-01	Y220C	Missense	chr17	7578190	7578190	T	C
TCGA-DD-AACL-01	C238R	Missense	chr17	7577569	7577569	A	G
TCGA-ZP-A9D2-01	R280K	Missense	chr17	7577099	7577099	C	T
TCGA-BC-A10U-01	R213Q	Missense	chr17	7578211	7578211	C	T
TCGA-BC-A216-01	R248W	Missense	chr17	7577539	7577539	G	A
TCGA-DD-AAVU-01	L194R	Missense	chr17	7578268	7578268	A	C

TCGA-2Y-A9H8-01	G245D	Missense	chr17	7577547	7577547	C	T
TCGA-UB-A7MB-01	Y205C	Missense	chr17	7578235	7578235	T	C
TCGA-DD-AADL-01	Y205C	Missense	chr17	7578235	7578235	T	C
TCGA-FV-A4ZP-01	E286K	Missense	chr17	7577082	7577082	C	T
TCGA-BW-A5NO-01	G266R	Missense	chr17	7577142	7577142	C	T
TCGA-RG-A7D4-01	C275Y	Missense	chr17	7577114	7577114	C	T
TCGA-DD-A1EE-01	H193R	Missense	chr17	7578271	7578271	T	C
TCGA-G3-A25Z-01	H193R	Missense	chr17	7578271	7578271	T	C
TCGA-FV-A3I1-01	H193R	Missense	chr17	7578271	7578271	T	C
TCGA-MI-A75G-01	H193R	Missense	chr17	7578271	7578271	T	C
TCGA-UB-A7MF-01	S215I	Missense	chr17	7578205	7578205	C	A
TCGA-DD-A114-01	R249S	Missense	chr17	7577534	7577534	C	A
TCGA-G3-A25U-01	R249S	Missense	chr17	7577534	7577534	C	A
TCGA-CC-A3M9-01	R249S	Missense	chr17	7577534	7577534	C	A
TCGA-CC-A3MB-01	R249S	Missense	chr17	7577534	7577534	C	A
TCGA-CC-A5UE-01	R249S	Missense	chr17	7577534	7577534	C	A
TCGA-QA-A7B7-01	R249S	Missense	chr17	7577534	7577534	C	A
TCGA-CC-A7II-01	R249S	Missense	chr17	7577534	7577534	C	A
TCGA-ED-A7XP-01	R249S	Missense	chr17	7577534	7577534	C	A
TCGA-CC-A8HU-01	R249S	Missense	chr17	7577534	7577534	C	A
TCGA-CC-A8HV-01	R249S	Missense	chr17	7577534	7577534	C	A
TCGA-2Y-A9GS-01	R249S	Missense	chr17	7577534	7577534	C	A
TCGA-BC-A3KG-01	N239S	Missense	chr17	7577565	7577565	T	C
TCGA-2Y-A9GY-01	R213L	Missense	chr17	7578211	7578211	C	A
TCGA-FV-A4ZQ-01	G266V	Missense	chr17	7577141	7577141	C	A
TCGA-CC-A8HT-01	M237I	Missense	chr17	7577570	7577570	C	A
TCGA-BD-A3EP-01	P151H	Missense	chr17	7578478	7578478	G	T
TCGA-DD-	C176W	Missense	chr17	7578402	7578402	G	C

AADW-01							
TCGA-GJ-A3OU-01	C275R	Missense	chr17	7577115	7577115	A	G
TCGA-YA-A8S7-01	Y205S	Missense	chr17	7578235	7578235	T	G
TCGA-DD-AAEA-01	I195S	Missense	chr17	7578265	7578265	A	C
TCGA-G3-A7M6-01	S215N	Missense	chr17	7578205	7578205	C	T
TCGA-DD-AADB-01	R273S	Missense	chr17	7577121	7577121	G	T
TCGA-DD-AACB-01	D281E	Missense	chr17	7577095	7577095	G	C
TCGA-BC-A10R-01	Q136*	Nonsense	chr17	7578524	7578524	G	A
TCGA-G3-A25V-01	I251Sfs*94	fs del	chr17	7577531	7577531	G	-
TCGA-DD-AACU-01	M246V	Missense	chr17	7577545	7577545	T	C
TCGA-DD-AAD3-01	E271V	Missense	chr17	7577126	7577126	T	A
TCGA-RC-A7SK-01	R174W	Missense	chr17	7578410	7578410	T	A
TCGA-CC-5263-01	C277*	Nonsense	chr17	7577107	7577107	A	T
TCGA-2Y-A9HA-01	M237Cfs*10	fs del	chr17	7577572	7577572	T	-
TCGA-BC-A5W4-01	A276G	Missense	chr17	7577111	7577111	G	C
TCGA-DD-A3A7-01	C135*	Nonsense	chr17	7578525	7578525	G	T
TCGA-DD-AAD5-01	F341V	Missense	chr17	7574006	7574006	A	C
TCGA-G3-A5SJ-01	K139N	Missense	chr17	7578513	7578513	C	A
TCGA-DD-A39Y-01	R158H	Missense	chr17	7578457	7578457	C	T
TCGA-MI-A75I-01	R158H	Missense	chr17	7578457	7578457	C	T
TCGA-FV-A3R2-01	R306*	Nonsense	chr17	7577022	7577022	G	A
TCGA-4R-AA8I-01	P191del	if del	chr17	7578275	7578277	GAG	-
TCGA-T1-A6J8-01	X187_splice	Splice	chr17	7578290	7578290	C	T
TCGA-CC-A5UD-01	V157F	Missense	chr17	7578461	7578461	C	A
TCGA-CC-A7IJ-01	V157F	Missense	chr17	7578461	7578461	C	A
TCGA-DD-AAVV-01	V157F	Missense	chr17	7578461	7578461	C	A
TCGA-BW-A5NQ-01	Q192*	Nonsense	chr17	7578275	7578275	G	A
TCGA-DD-	Q192*	Nonsense	chr17	7578275	7578275	G	A

AACG-01							
TCGA-K7-AAU7-01	R156P	Missense	chr17	7578463	7578463	C	G
TCGA-WJ-A86L-01	E204*	Nonsense	chr17	7578239	7578239	C	A
TCGA-CC-A7IG-01	E221*	Nonsense	chr17	7578188	7578188	C	A
TCGA-CC-A3MA-01	F113C	Missense	chr17	7579349	7579349	A	C
TCGA-CC-A1HT-01	X126_splice	Splice	chr17	7578555	7578555	C	T
TCGA-DD-AADC-01	X126_splice	Splice	chr17	7578555	7578555	C	T
TCGA-DD-AAED-01	X126_splice	Splice	chr17	7578555	7578555	C	T
TCGA-RC-A7SH-01	V197L	Missense	chr17	7578260	7578260	C	A
TCGA-DD-AADV-01	S106R	Missense	chr17	7579369	7579369	G	C
TCGA-DD-AACS-01	Y126N	Missense	chr17	7578554	7578554	A	T
TCGA-UB-A7MD-01	X33_splice	Splice	chr17	7579591	7579591	C	T
TCGA-DD-A1EB-01	Y126D	Missense	chr17	7578554	7578554	A	C
TCGA-DD-AACE-01	F328Sfs*17	fs del	chr17	7576863	7576863	A	-
TCGA-DD-AACV-01	Q144*	Nonsense	chr17	7578500	7578500	G	A
TCGA-DD-A1EG-01	V157G	Missense	chr17	7578460	7578460	A	C
TCGA-G3-A3CJ-01	V143M	Missense	chr17	7578503	7578503	C	T
TCGA-RC-A6M6-01	K292*	Nonsense	chr17	7577064	7577064	T	A
TCGA-2Y-A9H4-01	S90Pfs*33	fs del	chr17	7579420	7579420	G	-
TCGA-5C-AAPD-01	L43*	fs del	chr17	7579559	7579559	A	-
TCGA-K7-A5RG-01	X307_splice	Splice	chr17	7576928	7576928	T	C
TCGA-WX-AA44-01	X307_splice	Splice	chr17	7576928	7576928	T	C
TCGA-G3-AAV4-01	E224E	splice_region	chr17	7578177	7578177	C	T
TCGA-G3-A5SM-01	X332_splice	Splice	chr17	7574035	7574035	T	C
TCGA-DD-A1EI-01	E171*	Nonsense	chr17	7578419	7578419	C	A
TCGA-DD-AAD8-01	X225_splice	Splice	chr17	7577610	7577610	T	A
TCGA-RC-A7SB-01	E258K	Missense	chr17	7577509	7577509	C	T
TCGA-DD-A1EL-	A161S	Missense	chr17	7578449	7578449	C	A

01							
TCGA-DD-A11A-01	V157_R158dup	if ins	chr17	7578454	7578455	NA	CGCGGA
TCGA-DD-AAE6-01	V143Afs*29	fs ins	chr17	7578502	7578503	NA	CAGGG
TCGA-NI-A8LF-01	A276Lfs*31	fs ins	chr17	7577112	7577113	NA	ACAA
TCGA-ED-A7XO-01	L257P	Missense	chr17	7577511	7577511	A	G
TCGA-BC-A69H-01	V97Efs*48	fs del	chr17	7579389	7579399	GGGAAGGGACA	-
TCGA-BC-A8YO-01	T230_T231del	if del	chr17	7577589	7577594	GTGGTA	-
TCGA-CC-5258-01	N263Ifs*82	fs del	chr17	7577150	7577150	T	-
TCGA-CC-A7IK-01	Q317Ffs*9	fs del	chr17	7576866	7576897	TATTCTCCATCCAGTGGTTTCTTCTTTGGCTG	-
TCGA-DD-AACF-01	V143Rfs*18	fs del	chr17	7578477	7578504	GGGTGTGGAATCAACCCACAGCTGCACA	-
TCGA-DD-AACQ-01	D228Vfs*18	fs del	chr17	7577595	7577598	CAGT	-
TCGA-DD-AACZ-01	N263Kfs*9	fs ins	chr17	7577149	7577150	-	C
TCGA-DD-AADN-01	E204Vfs*4	fs del	chr17	7578237	7578238	CT	-
TCGA-DD-AAE0-01	T253A	Missense	chr17	7577524	7577524	T	C
TCGA-DD-AAEI-01	E56Kfs*67	fs del	chr17	7579521	7579521	C	-
TCGA-ED-A7PZ-01	R248Hfs*13	fs del	chr17	7577531	7577538	GGGCCTCC	-
TCGA-ES-A2HT-01	S227P	Missense	chr17	7577602	7577602	A	G
TCGA-FV-A496-01	Y103Tfs*20	fs del	chr17	7579380	7579380	A	-

TCGA-G3-A7M9-01	L194Efs *51	fs del	chr17	7578264	7578270	GAT AAG A	-
TCGA-G3-AAV7-01	G262Vfs *83	fs del	chr17	7577153	7577153	C	-

Table S12. Comparison table of TP53 mutation between HFGC and LFGC among PLC cohorts

Cohort	Class	TP53 MUT	TP53 *DBD MUT	TP53 WT	Fisher's exact test (p)	
					MUT vs. WT	DBD MUT vs. WT
TIGER_LC (n=152)	HFGC	55	38	46	2.85E-04	5.21E-04
	LFGC	12	7	39		
Thai_HCC (n=62)	HFGC	23	19	25	0.24	4.20E-02
	LFGC	4	1	10		
Thai_iCCA (n=90)	HFGC	32	19	21	4.78E-04	7.00E-03
	LFGC	8	6	29		
TCGA_HCC (n=364)	HFGC	104	59	192	3.14E-05	3.16E-03
	LFGC	7	5	61		

* DBD MUT : mutation occurred within DNA binding domain (DBD)

Table S13a. Table for tFA of TCGA SKCM cohort

Sample ID	tFA	Group	subset
TCGA-ER-A1A1-06A-11R-A18U-07	1833.533	Low	subset1
TCGA-RP-A690-06A-11R-A311-07	1975.275	NA	subset1
TCGA-EE-A2GU-06A-11R-A18T-07	1861.785	Low	subset1
TCGA-EE-A3JE-06A-11R-A20F-07	1861.778	Low	subset1
TCGA-BF-AAOX-01A-11R-A39D-07	2439.111	High	subset1
TCGA-ER-A19P-06A-11R-A18S-07	2019.652	NA	subset1
TCGA-D3-A2J6-06A-11R-A18T-07	2308.362	NA	subset1
TCGA-D9-A6EG-06A-12R-A32P-07	1846.107	Low	subset1
TCGA-WE-A8ZR-06A-11R-A37K-07	1766.808	Low	subset1
TCGA-EB-A82C-01A-11R-A352-07	2376.790	High	subset1
TCGA-FS-A1YY-06A-11R-A18T-07	2048.012	NA	subset1
TCGA-ER-A19T-06A-11R-A18U-07	2747.214	High	subset1
TCGA-D3-A51T-06A-11R-A266-07	2083.324	NA	subset1
TCGA-EE-A3AF-06A-11R-A18S-07	2319.922	NA	subset1
TCGA-QB-AA9O-06A-11R-A39D-07	2551.261	High	subset2
TCGA-EE-A29R-06A-11R-A18T-07	2071.334	NA	subset1
TCGA-D3-A2J9-06A-11R-A18T-07	1963.680	NA	subset1
TCGA-FS-A4FC-06A-11R-A24X-07	1776.473	Low	subset1
TCGA-D3-A3C8-06A-12R-A18S-07	2114.548	NA	subset1
TCGA-EE-A2GJ-06A-11R-A18U-07	1769.805	Low	subset1
TCGA-EB-A5UL-06A-11R-A311-07	2132.368	NA	subset1
TCGA-D9-A1X3-06A-11R-A18S-07	1800.016	Low	subset1
TCGA-Z2-AA3V-06A-11R-A39D-07	2945.241	High	subset1
TCGA-BF-A5EP-01A-12R-A27Q-07	2282.277	NA	subset1
TCGA-FS-A1Z4-06A-11R-A18T-07	1702.189	Low	subset1
TCGA-EB-A5SG-06A-11R-A311-07	2307.041	NA	subset1
TCGA-D3-A3ML-06A-11R-A21D-07	1974.338	NA	subset1
TCGA-EE-A2M6-06A-12R-A18S-07	2023.653	NA	subset1
TCGA-D3-A2JE-06A-11R-A37K-07	2037.189	NA	subset1
TCGA-D3-A8GD-06A-11R-A37K-07	2183.112	NA	subset1
TCGA-ER-A42K-06A-11R-A24X-07	2062.055	NA	subset1
TCGA-EE-A2MQ-06A-11R-A18S-07	2026.100	NA	subset1
TCGA-DA-A3F8-06A-11R-A20F-07	1998.634	NA	subset1
TCGA-GN-A265-06A-21R-A18T-07	2176.448	NA	subset1
TCGA-EE-A3AA-06A-11R-A18S-07	1735.311	Low	subset1
TCGA-EE-A3J8-06A-11R-A20F-07	1711.218	Low	subset1
TCGA-FR-A7U9-06A-11R-A352-07	2339.388	High	subset1
TCGA-EB-A5SE-01A-11R-A311-07	2178.593	NA	subset1
TCGA-DA-A1IC-06A-11R-A18S-07	2023.989	NA	subset1

TCGA-ER-A3EV-06A-11R-A20F-07	2619.586	High	subset1
TCGA-EB-A551-01A-21R-A27Q-07	1833.502	Low	subset1
TCGA-EE-A2ME-06A-11R-A18T-07	1648.314	Low	subset1
TCGA-EE-A29S-06A-11R-A18T-07	2153.671	NA	subset1
TCGA-D3-A1QA-06A-11R-A18T-07	1841.090	Low	subset1
TCGA-EE-A2A0-06A-11R-A18T-07	1616.215	Low	subset1
TCGA-FW-A5DY-06A-11R-A311-07	1924.269	NA	subset1
TCGA-D3-A5GS-06A-11R-A27Q-07	2074.785	NA	subset1
TCGA-BF-A3DN-01A-11R-A20F-07	1723.537	Low	subset1
TCGA-FS-A1Z7-06A-11R-A18T-07	1973.111	NA	subset1
TCGA-EE-A29L-06A-12R-A18S-07	1920.932	NA	subset1
TCGA-WE-A8ZM-06A-11R-A37K-07	2074.598	NA	subset1
TCGA-BF-A5EO-01A-12R-A27Q-07	3161.755	High	subset1
TCGA-FS-A1ZU-06A-12R-A18T-07	2372.705	High	subset1
TCGA-WE-AA9Y-06A-12R-A38C-07	2221.827	NA	subset1
TCGA-D3-A3CF-06A-11R-A18T-07	1960.643	NA	subset1
TCGA-D3-A1Q1-06A-21R-A18T-07	2314.370	NA	subset1
TCGA-D3-A3C3-06A-12R-A18S-07	2487.458	High	subset1
TCGA-D9-A3Z4-01A-11R-A239-07	2181.147	NA	subset1
TCGA-ER-A2NF-01A-11R-A18T-07	2351.492	High	subset1
TCGA-FS-A1ZT-06A-11R-A18U-07	1802.765	Low	subset1
TCGA-D3-A8GB-06A-11R-A37K-07	2413.124	High	subset1
TCGA-D3-A8GP-06A-11R-A37K-07	2128.930	NA	subset1
TCGA-WE-A8ZY-06A-11R-A37K-07	2165.085	NA	subset1
TCGA-FS-A1YX-06A-11R-A18T-07	1874.948	Low	subset1
TCGA-EB-A3XB-01A-11R-A239-07	2013.839	NA	subset1
TCGA-ER-A19S-06A-11R-A18U-07	2074.735	NA	subset1
TCGA-EE-A2MR-06A-11R-A18S-07	1864.706	Low	subset1
TCGA-D3-A8GE-06A-11R-A37K-07	1713.446	Low	subset1
TCGA-FS-A1YW-06A-11R-A18T-07	1753.762	Low	subset1
TCGA-GN-A4U5-01A-11R-A32P-07	2380.036	High	subset1
TCGA-HR-A2OH-06A-11R-A18U-07	2112.931	NA	subset1
TCGA-D3-A8GS-06A-12R-A37K-07	2234.734	NA	subset1
TCGA-D3-A8GN-06A-11R-A37K-07	2336.416	High	subset1
TCGA-EE-A2MP-06A-11R-A18S-07	2230.703	NA	subset1
TCGA-EB-A5VU-01A-21R-A32P-07	1984.819	NA	subset1
TCGA-EE-A2GN-06A-11R-A18S-07	2178.228	NA	subset1
TCGA-GN-A8LN-01A-11R-A37K-07	1894.746	Low	subset2
TCGA-FR-A8YE-06A-11R-A37K-07	1907.599	Low	subset1
TCGA-EE-A185-06A-11R-A18S-07	2238.907	NA	subset1
TCGA-ER-A195-06A-11R-A18U-07	1850.852	Low	subset1

TCGA-GF-A769-01A-32R-A32P-07	2011.733	NA	subset1
TCGA-D3-A2JH-06A-11R-A18T-07	1969.564	NA	subset1
TCGA-EE-A29V-06A-12R-A18S-07	2721.253	High	subset1
TCGA-EB-A5SH-06A-11R-A311-07	3147.432	High	subset1
TCGA-D3-A1QA-07A-11R-A37K-07	1887.884	Low	subset1
TCGA-EB-A3HV-01A-11R-A21D-07	2765.014	High	subset1
TCGA-GF-A2C7-01A-11R-A18T-07	2015.868	NA	subset1
TCGA-DA-A1I4-06A-11R-A18U-07	1947.043	NA	subset1
TCGA-D3-A2JF-06A-11R-A18S-07	2355.263	High	subset1
TCGA-FR-A726-01A-11R-A32P-07	1931.381	NA	subset1
TCGA-D3-A3BZ-06A-12R-A18S-07	2145.162	NA	subset1
TCGA-DA-A1IB-06A-11R-A18S-07	1985.401	NA	subset1
TCGA-GN-A266-06A-11R-A18T-07	1911.179	Low	subset1
TCGA-EE-A2M8-06A-12R-A18S-07	1986.732	NA	subset1
TCGA-EE-A2A6-06A-11R-A18T-07	1945.138	NA	subset1
TCGA-ER-A2NF-06A-11R-A18T-07	2286.245	NA	subset1
TCGA-ER-A3ET-06A-11R-A20F-07	1875.620	Low	subset1
TCGA-FR-A3YN-06A-11R-A239-07	1987.026	NA	subset2
TCGA-EE-A3AH-06A-11R-A18S-07	2073.417	NA	subset1
TCGA-FR-A8YC-06A-11R-A37K-07	2146.297	NA	subset1
TCGA-FS-A1Z3-06A-11R-A18T-07	1643.659	Low	subset1
TCGA-D3-A2JP-06A-11R-A18S-07	2377.688	High	subset1
TCGA-RP-A695-06A-11R-A311-07	2045.651	NA	subset1
TCGA-WE-A8ZX-06A-11R-A37K-07	2318.953	NA	subset1
TCGA-DA-A95Y-06A-11R-A37K-07	2549.316	High	subset1
TCGA-ER-A19W-06A-41R-A239-07	1931.592	NA	subset1
TCGA-FS-A1ZQ-06A-11R-A18U-07	2542.477	High	subset1
TCGA-D3-A51G-06A-11R-A266-07	1840.934	Low	subset1
TCGA-GF-A4EO-06A-12R-A24X-07	1970.318	NA	subset1
TCGA-D9-A1JW-06A-11R-A18S-07	2448.345	High	subset1
TCGA-EE-A20C-06A-11R-A18S-07	2139.490	NA	subset1
TCGA-EE-A29X-06A-11R-A18T-07	2541.010	High	subset1
TCGA-EE-A2MM-06A-11R-A18S-07	2028.837	NA	subset1
TCGA-EE-A3JB-06A-11R-A21D-07	1986.424	NA	subset1
TCGA-EE-A2MK-06A-11R-A18S-07	2095.734	NA	subset1
TCGA-DA-A1HW-06A-11R-A18U-07	1973.726	NA	subset1
TCGA-FS-A1ZD-06A-11R-A18T-07	1624.258	Low	subset1
TCGA-FS-A1ZP-06A-11R-A18T-07	1777.354	Low	subset1
TCGA-D3-A2JL-06A-11R-A18S-07	2534.391	High	subset1
TCGA-BF-AAP2-01A-11R-A40A-07	2303.308	NA	subset1
TCGA-D3-A2JB-06A-11R-A18T-07	2070.220	NA	subset1

TCGA-D3-A3CE-06A-11R-A18S-07	1758.624	Low	subset1
TCGA-GF-A6C9-06A-11R-A311-07	2416.296	High	subset1
TCGA-ER-A19K-01A-21R-A18T-07	2027.554	NA	subset1
TCGA-W3-AA1V-06B-11R-A40A-07	1847.777	Low	subset1
TCGA-EB-A97M-01A-11R-A38C-07	2843.162	High	subset1
TCGA-EE-A29N-06A-12R-A18S-07	1758.215	Low	subset1
TCGA-EB-A4OZ-01A-12R-A266-07	3544.320	High	subset1
TCGA-W3-AA21-06A-11R-A38C-07	2925.908	High	subset1
TCGA-ER-A19F-06A-11R-A18S-07	2015.959	NA	subset1
TCGA-EE-A3AB-06A-11R-A18S-07	2371.241	High	subset1
TCGA-EB-A431-01A-11R-A266-07	1962.443	NA	subset1
TCGA-XV-A9W2-01A-11R-A39D-07	2268.226	NA	subset1
TCGA-BF-AAOU-01A-12R-A39D-07	2333.175	High	subset1
TCGA-EE-A2M5-06A-12R-A18S-07	1914.413	NA	subset1
TCGA-YG-AA3O-06A-11R-A38C-07	1926.656	NA	subset1
TCGA-D3-A8GJ-06A-11R-A37K-07	2290.560	NA	subset1
TCGA-EB-A550-01A-61R-A27Q-07	2053.782	NA	subset1
TCGA-FR-A729-06A-11R-A352-07	2166.960	NA	subset1
TCGA-ER-A42H-01A-11R-A24X-07	2329.346	High	subset1
TCGA-DA-A1IA-06A-11R-A18S-07	3272.298	High	subset1
TCGA-EB-A42Y-01A-12R-A24X-07	2126.079	NA	subset1
TCGA-LH-A9QB-06A-11R-A38C-07	1597.084	Low	subset1
TCGA-BF-A1Q0-01A-21R-A18S-07	2191.620	NA	subset1
TCGA-FS-A1ZB-06A-12R-A18S-07	2298.871	NA	subset1
TCGA-D3-A51R-06A-11R-A266-07	1856.005	Low	subset1
TCGA-FS-A1ZC-06A-11R-A18T-07	1928.130	NA	subset1
TCGA-EB-A41B-01A-11R-A24X-07	2602.208	High	subset1
TCGA-DA-A1HV-06A-21R-A18S-07	2240.725	NA	subset1
TCGA-FR-A44A-06A-11R-A24X-07	2060.373	NA	subset1
TCGA-EE-A2GL-06A-11R-A18S-07	2030.395	NA	subset1
TCGA-EE-A3J7-06A-11R-A20F-07	1923.413	NA	subset1
TCGA-EB-A553-01A-12R-A27Q-07	2742.727	High	subset1
TCGA-D3-A1Q4-06A-11R-A18T-07	2087.720	NA	subset1
TCGA-EE-A2MC-06A-12R-A18S-07	1912.422	Low	subset1
TCGA-EE-A2MN-06A-11R-A18S-07	2253.975	NA	subset1
TCGA-BF-A1PV-01A-11R-A18U-07	1816.889	Low	subset1
TCGA-3N-A9WD-06A-11R-A38C-07	2125.827	NA	subset1
TCGA-WE-A8ZN-06A-11R-A37K-07	3033.920	High	subset1
TCGA-BF-AAP7-01A-11R-A40A-07	2843.033	High	subset1
TCGA-EE-A2A1-06A-11R-A18T-07	2106.479	NA	subset1
TCGA-EE-A3JA-06A-11R-A20F-07	1734.669	Low	subset1

TCGA-EE-A29C-06A-21R-A18S-07	2411.624	High	subset2
TCGA-D3-A3MO-06A-11R-A21D-07	1982.460	NA	subset1
TCGA-ER-A19G-06A-11R-A18U-07	1930.281	NA	subset1
TCGA-FS-A1ZA-06A-11R-A18T-07	2030.301	NA	subset1
TCGA-EE-A181-06A-11R-A18S-07	1996.543	NA	subset1
TCGA-EE-A2GB-06A-11R-A18T-07	1719.227	Low	subset1
TCGA-EE-A2GI-06A-11R-A18T-07	2339.467	High	subset1
TCGA-D9-A6E9-06A-12R-A311-07	2542.533	High	subset1
TCGA-D3-A2JN-06A-11R-A18S-07	2213.550	NA	subset1
TCGA-DA-A1I2-06A-21R-A18U-07	1859.553	Low	subset1
TCGA-FW-A3R5-06A-11R-A239-07	1734.250	Low	subset1
TCGA-BF-A5EQ-01A-21R-A27Q-07	2156.379	NA	subset1
TCGA-EB-A3Y7-01A-11R-A239-07	2995.436	High	subset1
TCGA-WE-AAA3-06A-11R-A38C-07	2409.066	High	subset1
TCGA-BF-A3DM-01A-11R-A20F-07	1867.547	Low	subset1
TCGA-GF-A6C8-06A-12R-A311-07	2117.417	NA	subset1
TCGA-EB-A3Y6-01A-21R-A239-07	2084.139	NA	subset1
TCGA-FS-A1ZS-06A-12R-A18T-07	1727.620	Low	subset1
TCGA-EB-A5VV-06A-11R-A32P-07	2086.798	NA	subset1
TCGA-FS-A1ZM-06A-12R-A18S-07	2005.679	NA	subset1
TCGA-D3-A5GU-06A-11R-A27Q-07	2030.325	NA	subset1
TCGA-GN-A267-06A-21R-A18T-07	2433.771	High	subset1
TCGA-XV-A9VZ-01A-11R-A38C-07	2080.786	NA	subset1
TCGA-FW-A5DX-01A-11R-A27Q-07	1873.973	Low	subset1
TCGA-YD-A9TA-06A-11R-A39D-07	1855.413	Low	subset1
TCGA-GN-A262-06A-11R-A18T-07	2511.234	High	subset1
TCGA-WE-A8K6-06A-11R-A37K-07	2211.806	NA	subset1
TCGA-D3-A3CC-06A-11R-A18S-07	1986.728	NA	subset1
TCGA-FS-A4F5-06A-11R-A266-07	1949.295	NA	subset1
TCGA-FS-A1ZK-06A-11R-A18T-07	1966.155	NA	subset1
TCGA-EB-A85J-01A-12R-A352-07	2465.963	High	subset1
TCGA-EB-A6QZ-01A-12R-A32P-07	2285.247	NA	subset1
TCGA-DA-A3F2-06A-11R-A20F-07	1916.093	NA	subset1
TCGA-D9-A149-06A-11R-A18S-07	2179.247	NA	subset1
TCGA-EE-A180-06A-11R-A21D-07	1962.510	NA	subset1
TCGA-D9-A3Z3-06A-11R-A239-07	2329.837	High	subset1
TCGA-GN-A9SD-06A-11R-A40A-07	2121.817	NA	subset1
TCGA-EE-A2MI-06A-11R-A18U-07	1770.823	Low	subset1
TCGA-DA-A1I7-06A-22R-A18S-07	2155.671	NA	subset1
TCGA-W3-AA1Q-06A-11R-A38C-07	2080.683	NA	subset1
TCGA-BF-A9VF-01A-11R-A37K-07	2950.453	High	subset1

TCGA-W3-AA1R-06A-11R-A39D-07	2231.668	NA	subset1
TCGA-GN-A4U3-06A-11R-A32P-07	2117.681	NA	subset1
TCGA-RP-A693-06A-13R-A311-07	1956.734	NA	subset1
TCGA-D3-A5GO-06A-12R-A27Q-07	2364.763	High	subset1
TCGA-XV-AAZV-01A-11R-A40A-07	2662.743	High	subset1
TCGA-ER-A193-06A-12R-A18S-07	1985.362	NA	subset1
TCGA-D3-A5GL-06A-11R-A27Q-07	2007.073	NA	subset1
TCGA-D3-A3MU-06A-11R-A21D-07	2541.853	High	subset1
TCGA-EE-A29P-06A-11R-A18T-07	2028.607	NA	subset1
TCGA-BF-AAP6-01A-11R-A40A-07	2012.012	NA	subset1
TCGA-EB-A3XD-01A-22R-A239-07	2593.070	High	subset1
TCGA-XV-AAZY-01A-12R-A40A-07	3290.632	High	subset1
TCGA-EE-A2GE-06A-11R-A18T-07	2048.518	NA	subset1
TCGA-EB-A5KH-06A-11R-A27Q-07	2802.093	High	subset1
TCGA-D3-A8GQ-06A-11R-A37K-07	2308.990	NA	subset1
TCGA-D3-A8GL-06A-11R-A37K-07	1959.640	NA	subset1
TCGA-GN-A268-06A-11R-A18T-07	1933.873	NA	subset1
TCGA-FS-A1ZR-06A-21R-A18U-07	1835.580	Low	subset1
TCGA-D3-A1Q6-06A-11R-A18T-07	1544.720	Low	subset1
TCGA-EE-A3AC-06A-11R-A18S-07	1901.392	Low	subset1
TCGA-EE-A29H-06A-12R-A18S-07	2223.850	NA	subset1
TCGA-D3-A1Q3-06A-11R-A18T-07	1764.291	Low	subset1
TCGA-GN-A26C-01A-11R-A18T-07	2226.242	NA	subset1
TCGA-EE-A2GM-06B-11R-A18S-07	1931.751	NA	subset1
TCGA-D3-A1Q8-06A-11R-A18T-07	2000.435	NA	subset1
TCGA-BF-AAP0-06A-11R-A39D-07	2329.012	NA	subset1
TCGA-EB-A3XE-01A-12R-A239-07	2935.296	High	subset1
TCGA-ER-A2NB-01A-12R-A18S-07	2398.038	High	subset1
TCGA-HR-A2OG-06A-21R-A18U-07	1885.062	Low	subset1
TCGA-GN-A26A-06A-11R-A18T-07	2213.402	NA	subset1
TCGA-D9-A4Z2-01A-11R-A24X-07	1956.883	NA	subset1
TCGA-ER-A2NG-06A-11R-A18T-07	2086.277	NA	subset1
TCGA-XV-AAZW-01A-12R-A40A-07	2405.637	High	subset1
TCGA-D3-A51N-06A-11R-A266-07	2375.036	High	subset1
TCGA-ER-A42L-06A-11R-A24X-07	1884.028	Low	subset1
TCGA-W3-A828-06A-11R-A352-07	1728.455	Low	subset1
TCGA-D3-A51H-06A-12R-A266-07	2163.153	NA	subset1
TCGA-D3-A8GK-06A-11R-A37K-07	2323.482	NA	subset1
TCGA-YD-A89C-06A-11R-A37K-07	2257.450	NA	subset1
TCGA-EB-A3XF-01A-31R-A239-07	2003.544	NA	subset1
TCGA-WE-A8K4-01A-12R-A37K-07	2094.691	NA	subset1

TCGA-EE-A29E-06A-11R-A18T-07	1956.071	NA	subset1
TCGA-FS-A1ZJ-06A-12R-A18S-07	2172.719	NA	subset1
TCGA-ER-A19A-06A-21R-A18U-07	1892.173	Low	subset1
TCGA-DA-A1I0-06A-11R-A20F-07	3273.873	High	subset1
TCGA-EE-A182-06A-11R-A18T-07	3470.002	High	subset1
TCGA-DA-A95V-06A-11R-A37K-07	3114.335	High	subset1
TCGA-EE-A2MD-06A-11R-A18T-07	1836.676	Low	subset1
TCGA-FS-A1ZZ-06A-11R-A18S-07	1858.423	Low	subset1
TCGA-FS-A4F4-06A-12R-A266-07	2834.993	High	subset1
TCGA-GN-A263-01A-11R-A18T-07	1761.940	Low	subset1
TCGA-RP-A694-06A-11R-A311-07	1899.378	Low	subset1
TCGA-ER-A194-01A-11R-A18U-07	3309.356	High	subset1
TCGA-EE-A29B-06A-11R-A18U-07	2639.422	High	subset1
TCGA-GN-A4U7-06A-21R-A32P-07	2605.683	High	subset1
TCGA-EE-A3J3-06A-11R-A20F-07	2069.494	NA	subset1
TCGA-D3-A3CB-06A-11R-A18S-07	1964.008	NA	subset1
TCGA-FR-A2OS-01A-11R-A21D-07	1640.405	Low	subset1
TCGA-GN-A264-06A-11R-A18U-07	1909.148	Low	subset1
TCGA-EB-A85I-01A-11R-A352-07	2645.651	High	subset1
TCGA-D9-A148-06A-11R-A18S-07	2325.850	NA	subset1
TCGA-ER-A19T-01A-11R-A18T-07	2445.725	High	subset1
TCGA-D3-A51F-06A-11R-A266-07	2176.655	NA	subset1
TCGA-D9-A4Z6-06A-12R-A266-07	2008.184	NA	subset1
TCGA-D3-A8GI-06A-11R-A37K-07	3280.004	High	subset1
TCGA-WE-A8ZQ-06A-41R-A37K-07	2016.299	NA	subset1
TCGA-EE-A29A-06A-12R-A18U-07	1970.783	NA	subset1
TCGA-GN-A4U4-06A-11R-A32P-07	1906.754	Low	subset2
TCGA-EB-A41A-01A-11R-A24X-07	2100.956	NA	subset1
TCGA-FW-A3TV-06A-11R-A239-07	1771.361	Low	subset1
TCGA-BF-A5ES-01A-11R-A27Q-07	2474.821	High	subset1
TCGA-EB-A24D-01A-11R-A18T-07	2042.970	NA	subset1
TCGA-D3-A1Q7-06A-11R-A18T-07	1796.908	Low	subset1
TCGA-EE-A184-06A-11R-A18S-07	2126.913	NA	subset1
TCGA-D3-A1Q5-06A-11R-A18T-07	1751.546	Low	subset1
TCGA-D9-A4Z3-01A-11R-A266-07	2142.430	NA	subset1
TCGA-FS-A4F2-06A-11R-A24X-07	2296.874	NA	subset1
TCGA-ER-A2NH-06A-11R-A18S-07	2074.206	NA	subset1
TCGA-D3-A8GR-06A-11R-A37K-07	2804.760	High	subset1
TCGA-W3-AA1O-06A-11R-A38C-07	1843.408	Low	subset1
TCGA-WE-A8ZO-06A-11R-A37K-07	1859.589	Low	subset1
TCGA-EE-A2GR-06A-11R-A18S-07	1757.063	Low	subset1

TCGA-ER-A19O-06A-11R-A18S-07	2365.430	High	subset1
TCGA-D3-A3C7-06A-11R-A18U-07	2233.500	NA	subset1
TCGA-EB-A82B-01A-11R-A352-07	2657.561	High	subset1
TCGA-EB-A6L9-06A-11R-A32P-07	2948.606	High	subset1
TCGA-DA-A1I8-06A-11R-A18T-07	2050.480	NA	subset1
TCGA-ER-A2NC-06A-11R-A18T-07	2002.037	NA	subset1
TCGA-EB-A3XC-01A-11R-A239-07	2406.407	High	subset1
TCGA-EE-A29G-06A-12R-A18T-07	1986.555	NA	subset1
TCGA-BF-A3DL-01A-11R-A20F-07	2798.171	High	subset1
TCGA-EE-A2GD-06A-11R-A18T-07	1819.240	Low	subset1
TCGA-EE-A2MU-06A-21R-A18S-07	2107.591	NA	subset1
TCGA-EE-A2GT-06A-12R-A18S-07	2370.242	High	subset1
TCGA-ER-A19J-06A-11R-A18S-07	2056.954	NA	subset1
TCGA-FS-A4F8-06A-11R-A266-07	1910.427	Low	subset1
TCGA-EB-A44R-06A-41R-A266-07	2303.148	NA	subset1
TCGA-EB-A4IQ-01A-12R-A266-07	2373.122	High	subset1
TCGA-Z2-A8RT-06A-11R-A37K-07	2126.651	NA	subset1
TCGA-GN-A4U9-06A-11R-A32P-07	2266.429	NA	subset2
TCGA-GN-A8LK-06A-11R-A37K-07	2929.237	High	subset2
TCGA-D3-A5GT-01A-12R-A311-07	2591.782	High	subset1
TCGA-D3-A2JG-06A-11R-A18T-07	2215.628	NA	subset1
TCGA-YG-AA3N-01A-11R-A38C-07	2798.735	High	subset1
TCGA-EE-A2A2-06A-11R-A18T-07	1915.004	NA	subset1
TCGA-EE-A29Q-06A-11R-A18T-07	1741.164	Low	subset1
TCGA-EB-A51B-01A-11R-A27Q-07	2039.143	NA	subset1
TCGA-Z2-AA3S-06A-11R-A39D-07	1943.714	NA	subset1
TCGA-D3-A1Q9-06A-11R-A18T-07	1840.123	Low	subset1
TCGA-FS-A1ZH-06A-11R-A18T-07	1976.220	NA	subset1
TCGA-EB-A5UN-06A-11R-A311-07	1735.959	Low	subset1
TCGA-D3-A51E-06A-11R-A266-07	2178.013	NA	subset1
TCGA-D3-A8GM-06A-11R-A37K-07	1750.134	Low	subset1
TCGA-D3-A8GO-06A-11R-A37K-07	2687.796	High	subset1
TCGA-WE-A8JZ-06A-11R-A37K-07	2113.723	NA	subset1
TCGA-EE-A183-06A-11R-A18S-07	2910.901	High	subset1
TCGA-EE-A17Z-06A-11R-A18S-07	2333.957	High	subset1
TCGA-EE-A20F-06A-21R-A18S-07	2113.717	NA	subset1
TCGA-EB-A44P-01A-11R-A266-07	1889.396	Low	subset1
TCGA-DA-A95W-06A-11R-A37K-07	2365.247	High	subset1
TCGA-EB-A42Z-01A-12R-A24X-07	2199.235	NA	subset1
TCGA-DA-A95Z-06A-11R-A37K-07	2345.368	High	subset1
TCGA-WE-A8K5-06A-11R-A37K-07	2233.435	NA	subset1

TCGA-ER-A197-06A-32R-A18S-07	2352.582	High	subset1
TCGA-FS-A1ZE-06A-11R-A18T-07	1833.423	Low	subset1
TCGA-EB-A4XL-01A-11R-A27Q-07	1943.764	NA	subset1
TCGA-XV-A9W5-01A-11R-A38C-07	2341.378	High	subset1
TCGA-EE-A3J4-06A-11R-A20F-07	2020.619	NA	subset1
TCGA-WE-A8K1-06A-21R-A37K-07	2234.531	NA	subset1
TCGA-EE-A2MJ-06A-11R-A18S-07	1899.382	Low	subset1
TCGA-DA-A1I5-06A-11R-A18T-07	2159.440	NA	subset1
TCGA-D3-A8GV-06A-11R-A37K-07	2415.200	High	subset1
TCGA-EB-A1NK-01A-11R-A18T-07	2610.533	High	subset1
TCGA-EE-A17Y-06A-11R-A18T-07	2010.815	NA	subset1
TCGA-D3-A2J7-06A-11R-A18T-07	1852.938	Low	subset1
TCGA-EE-A2GH-06A-11R-A18T-07	2353.014	High	subset1
TCGA-RP-A6K9-06A-41R-A352-07	2191.958	NA	subset1
TCGA-EE-A2GS-06A-12R-A18S-07	2147.036	NA	subset2
TCGA-EE-A2MH-06A-11R-A18S-07	2276.324	NA	subset1
TCGA-EB-A430-01A-11R-A24X-07	2618.281	High	subset1
TCGA-EE-A3AG-06A-31R-A18S-07	2084.292	NA	subset1
TCGA-D3-A51K-06A-11R-A266-07	3450.853	High	subset1
TCGA-EE-A17X-06A-11R-A18S-07	2312.433	NA	subset1
TCGA-ER-A196-01A-11R-A18T-07	2102.420	NA	subset1
TCGA-BF-A1PX-01A-12R-A18T-07	2431.812	High	subset1
TCGA-FR-A728-01A-11R-A32P-07	2239.871	NA	subset1
TCGA-FR-A7U8-06A-21R-A352-07	2662.407	High	subset1
TCGA-EE-A3J5-06A-11R-A20F-07	2054.339	NA	subset1
TCGA-HR-A5NC-01A-11R-A27Q-07	1966.957	NA	subset1
TCGA-3N-A9WC-06A-11R-A38C-07	2032.608	NA	subset1
TCGA-FS-A1ZN-01A-11R-A18T-07	1860.979	Low	subset1
TCGA-EE-A29W-06A-11R-A18U-07	1871.479	Low	subset1
TCGA-D9-A6EA-06A-11R-A311-07	2393.943	High	subset1
TCGA-W3-A824-06A-21R-A352-07	2300.339	NA	subset1
TCGA-EE-A2MF-06A-11R-A21D-07	1771.066	Low	subset1
TCGA-YG-AA3P-06A-11R-A38C-07	2343.788	High	subset1
TCGA-DA-A3F3-06A-11R-A20F-07	1871.956	Low	subset1
TCGA-FR-A8YD-06A-11R-A37K-07	2969.640	High	subset2
TCGA-QB-A6FS-06A-11R-A311-07	3457.167	High	subset1
TCGA-FS-A1ZY-06A-11R-A18S-07	2479.247	High	subset1
TCGA-EB-A44Q-06A-11R-A266-07	1920.895	NA	subset1
TCGA-WE-AAA0-06A-11R-A38C-07	2132.450	NA	subset2
TCGA-D3-A2JD-06A-11R-A18T-07	2929.576	High	subset1
TCGA-FR-A3R1-01A-11R-A239-07	1710.592	Low	subset1

TCGA-BF-A3DJ-01A-11R-A20F-07	2116.079	NA	subset1
TCGA-EE-A2GK-06A-11R-A18S-07	2087.017	NA	subset1
TCGA-EE-A29M-06A-11R-A18T-07	1895.099	Low	subset1
TCGA-FR-A3YO-06A-11R-A239-07	2886.871	High	subset2
TCGA-BF-A1PU-01A-11R-A18S-07	2413.662	High	subset1
TCGA-D3-A2JC-06A-11R-A18T-07	2294.340	NA	subset1
TCGA-D3-A1QB-06A-11R-A18T-07	2234.045	NA	subset1
TCGA-D3-A3C6-06A-12R-A18U-07	1970.091	NA	subset1
TCGA-ER-A199-06A-11R-A18T-07	2123.379	NA	subset1
TCGA-GN-A8LL-06A-21R-A37K-07	3005.469	High	subset1
TCGA-XV-AB01-06A-12R-A40A-07	3383.119	High	subset1
TCGA-EE-A2GP-06A-11R-A18S-07	1634.481	Low	subset1
TCGA-EE-A2MG-06A-11R-A18T-07	1850.515	Low	subset1
TCGA-BF-A5ER-01A-12R-A27Q-07	3188.303	High	subset1
TCGA-D3-A2J8-06A-11R-A18T-07	2195.415	NA	subset1
TCGA-DA-A1HY-06A-11R-A18T-07	1835.734	Low	subset1
TCGA-DA-A95X-06A-11R-A37K-07	1876.848	Low	subset1
TCGA-D3-A3C1-06A-12R-A18S-07	2675.555	High	subset1
TCGA-EB-A24C-01A-11R-A18T-07	1955.662	NA	subset1
TCGA-FS-A4F0-06A-11R-A24X-07	1692.776	Low	subset1
TCGA-EE-A3JD-06A-11R-A20F-07	1698.645	Low	subset1
TCGA-D9-A6EC-06A-11R-A311-07	2210.169	NA	subset1
TCGA-EE-A2MS-06A-11R-A18S-07	2148.495	NA	subset1
TCGA-FS-A4F9-06A-11R-A24X-07	1902.278	Low	subset1
TCGA-BF-A1PZ-01A-11R-A18S-07	2393.962	High	subset1
TCGA-EE-A20H-06A-11R-A18S-07	1814.914	Low	subset1
TCGA-FS-A4FD-06A-11R-A266-07	2136.244	NA	subset1
TCGA-D3-A5GR-06A-11R-A27Q-07	1829.309	Low	subset1
TCGA-EB-A57M-01A-51R-A311-07	2890.017	High	subset1
TCGA-EB-A299-01A-21R-A18U-07	2382.678	High	subset1
TCGA-EE-A20B-06A-11R-A18U-07	2121.893	NA	subset1
TCGA-EE-A2A5-06A-11R-A18T-07	2049.528	NA	subset1
TCGA-EB-A4IS-01A-21R-A266-07	2130.696	NA	subset1
TCGA-D3-A3MV-06A-11R-A21D-07	1986.410	NA	subset1
TCGA-EB-A6QY-01A-12R-A32P-07	1909.892	Low	subset1
TCGA-DA-A1I1-06A-12R-A18U-07	1948.713	NA	subset1
TCGA-ER-A19D-06A-11R-A18S-07	1773.560	Low	subset1
TCGA-3N-A9WB-06A-11R-A38C-07	2184.664	NA	subset1
TCGA-ER-A19B-06A-11R-A18S-07	2173.003	NA	subset1
TCGA-D3-A3MR-06A-11R-A21D-07	2390.621	High	subset1
TCGA-FW-A3I3-06A-11R-A21D-07	1884.355	Low	subset1

TCGA-EE-A2GC-06A-11R-A18T-07	1644.236	Low	subset1
TCGA-EB-A4OY-01A-11R-A266-07	3620.139	High	subset1
TCGA-ER-A3PL-06A-11R-A239-07	1991.540	NA	subset1
TCGA-EE-A2M7-06A-11R-A18U-07	1919.662	NA	subset1
TCGA-W3-A825-06A-11R-A352-07	2208.690	NA	subset1
TCGA-EB-A44O-01A-11R-A266-07	1785.798	Low	subset1
TCGA-D3-A8GC-06A-11R-A37K-07	1959.354	NA	subset1
TCGA-EB-A44N-01A-11R-A266-07	1988.082	NA	subset1
TCGA-DA-A960-01A-11R-A37K-07	3006.129	High	subset1
TCGA-FR-A7UA-06A-32R-A352-07	1883.542	Low	subset1
TCGA-ER-A19Q-06A-11R-A18U-07	2150.867	NA	subset1
TCGA-BF-AAP8-01A-11R-A40A-07	2284.551	NA	subset1
TCGA-D9-A4Z5-01A-11R-A266-07	2118.556	NA	subset1
TCGA-BF-AAP1-01A-11R-A39D-07	2271.943	NA	subset1
TCGA-OD-A75X-06A-12R-A32P-07	1690.400	Low	subset1
TCGA-EB-A6R0-01A-12R-A32P-07	1875.735	Low	subset1
TCGA-ER-A198-06A-11R-A18T-07	1774.436	Low	subset1
TCGA-WE-A8ZT-06A-11R-A37K-07	1534.970	Low	subset1
TCGA-EE-A20I-06A-11R-A18U-07	2230.602	NA	subset1
TCGA-FS-A1Z0-06A-11R-A18T-07	1778.477	Low	subset1
TCGA-ER-A19L-06A-12R-A18S-07	1909.123	Low	subset1
TCGA-FS-A1ZF-06A-12R-A18S-07	2206.751	NA	subset1
TCGA-DA-A3F5-06A-11R-A20F-07	2173.165	NA	subset1
TCGA-BF-AAP4-01A-11R-A40A-07	2253.955	NA	subset1
TCGA-FR-A69P-06A-21R-A311-07	1825.268	Low	subset1
TCGA-W3-AA1W-06A-11R-A38C-07	2113.790	NA	subset1
TCGA-D3-A51J-06A-11R-A266-07	2105.771	NA	subset1
TCGA-EB-A4P0-01A-41R-A266-07	2607.393	High	subset1
TCGA-D3-A5GN-06A-11R-A27Q-07	1867.903	Low	subset1
TCGA-ER-A2NE-06A-21R-A18T-07	2045.589	NA	subset1
TCGA-EE-A29T-06A-11R-A18T-07	1897.981	Low	subset1
TCGA-EE-A2GO-06A-11R-A18S-07	2001.053	NA	subset1
TCGA-EE-A3AD-06A-11R-A18S-07	1939.183	NA	subset1
TCGA-D9-A1JX-06A-11R-A18S-07	2034.000	NA	subset1
TCGA-EE-A3JH-06A-11R-A21D-07	1983.903	NA	subset1
TCGA-EE-A3JI-06A-11R-A21D-07	1692.108	Low	subset2
TCGA-EE-A29D-06A-11R-A18T-07	1953.450	NA	subset1
TCGA-GN-A26D-06A-11R-A18T-07	2011.655	NA	subset1
TCGA-GN-A4U8-06A-11R-A32P-07	2123.931	NA	subset1
TCGA-ER-A19H-06A-12R-A18S-07	1714.553	Low	subset1
TCGA-EE-A2ML-06A-11R-A18S-07	1785.597	Low	subset1

TCGA-ER-A3ES-06A-11R-A20F-07	1965.942	NA	subset1
TCGA-IH-A3EA-01A-11R-A20F-07	3069.875	High	subset1
TCGA-FS-A1ZG-06A-11R-A18T-07	1905.587	Low	subset1
TCGA-D9-A3Z1-06A-11R-A239-07	1895.923	Low	subset1
TCGA-YD-A9TB-06A-12R-A40A-07	1846.249	Low	subset1
TCGA-ER-A2ND-06A-11R-A18T-07	2667.364	High	subset1
TCGA-EB-A5SF-01A-11R-A311-07	2844.826	High	subset1
TCGA-D3-A2JK-06A-11R-A18S-07	2894.867	High	subset1
TCGA-FW-A3TU-06A-11R-A239-07	3110.687	High	subset1
TCGA-EB-A5FP-01A-11R-A27Q-07	1781.826	Low	subset1
TCGA-ER-A19E-06A-11R-A18S-07	2002.127	NA	subset1
TCGA-ER-A19M-06A-61R-A239-07	1969.996	NA	subset1
TCGA-D3-A2JO-06A-11R-A18S-07	1919.488	NA	subset1
TCGA-D3-A2JA-06A-11R-A18T-07	1929.326	NA	subset1
TCGA-GF-A3OT-06A-23R-A239-07	1986.806	NA	subset2
TCGA-ER-A19N-06A-11R-A18S-07	1962.540	NA	subset1
TCGA-EE-A2MT-06A-11R-A18S-07	1947.686	NA	subset1
TCGA-FS-A4FB-06A-11R-A266-07	1895.969	Low	subset1
TCGA-GN-A4U8-11A-11R-A32P-07	2167.931	NA	subset1
TCGA-EB-A5UM-01A-11R-A311-07	2956.515	High	subset1
TCGA-FS-A1ZW-06A-12R-A18T-07	1665.910	Low	subset1
TCGA-WE-AAA4-06A-12R-A38C-07	2905.925	High	subset1
TCGA-ER-A19C-06A-11R-A18S-07	1911.208	Low	subset1

Table S13b. Table for tFA of Hugo Melanoma cohort

Sample ID	tFA	Group
Pt1	2232.637	Low
Pt2	2731.533	High
Pt4	2598.993	Low
Pt5	2313.247	Low
Pt6	2283.931	Low
Pt7	2854.153	High
Pt8	3014.902	High
Pt9	2445.262	Low
Pt10	2452.072	Low
Pt12	2362.878	Low
Pt13	2301.223	Low
Pt14	2384.946	Low
Pt15	3063.404	High
Pt16	3016.530	High
Pt19	3672.679	High
Pt20	2736.222	High
Pt22	2258.911	Low
Pt23	2706.164	High
Pt25	3924.079	High
Pt27A	2668.609	Low
Pt27B	2637.624	Low
Pt28	2037.138	Low
Pt29	2697.004	High
Pt31	3363.477	High
Pt32	2847.857	High
Pt35	2508.802	Low
Pt37	3509.842	High
Pt38	2764.535	High
